# New Isomalabaricane-Derived Metabolites from a *Stelletta* sp. Marine Sponge

**DOI:** 10.3390/molecules26030678

**Published:** 2021-01-28

**Authors:** Sophia A. Kolesnikova, Ekaterina G. Lyakhova, Anastasia B. Kozhushnaya, Anatoly I. Kalinovsky, Dmitrii V. Berdyshev, Roman S. Popov, Valentin A. Stonik

**Affiliations:** 1 G.B. Elyakov Pacific Institute of Bioorganic Chemistry, Far Eastern Branch of Russian Academy of Sciences, Pr. 100-let Vladivostoku 159, 690022 Vladivostok, Russia; elyakhova@inbox.ru (E.G.L.); kaaniw@piboc.dvo.ru (A.I.K.); berdyshev@piboc.dvo.ru (D.V.B.); prs_90@mail.ru (R.S.P.); stonik@piboc.dvo.ru (V.A.S.); 2 School of Natural Sciences, Far Eastern Federal University, Sukhanova Str. 8, 690000 Vladivostok, Russia; kozhushnaia.ab@mail.ru

**Keywords:** isomalabaricanes, *Stelletta* sp., marine sponge, terpenoid, structure elucidation

## Abstract

In continuation of our studies on a Vietnamese collection of a *Stelletta* sp., sponge we have isolated two new isomalabaricane triterpenoids, stellettins Q and R (**1** and **2**), and four new isomalabaricane-derived *nor*-terpenoids, stellettins S-V **3**–**6**, along with previously known globostelletin N. Among them, compound **3** contains an acetylenic fragment, unprecedented in the isomalabaricane family and extremely rare in other marine sponge terpenoids. The structures and absolute configurations of all new compounds were established by extensive NMR, MS, and ECD analyses together with quantum-chemical modeling. Additionally, according to obtained new data we report the correction in stereochemistry of two asymmetric centers in the structures of two known isomalabaricanes, 15*R*,23*S* for globostelletin M and 15*S*,23*R* for globostelletin N.

## 1. Introduction

Malabaricanes are rather a small group of tricarbocyclic triterpenoids found in different tropical flowering terrestrial plants [1,2,3]. Isomalabaricanes, which differ from malabaricanes in the configuration of C-8 asymmetric center and have an α-oriented CH_3_-30, are known as metabolites of four genera of marine sponges—*Stelletta*, *Jaspis*, *Geodia* and *Rhabdastrella—*belonging to the class Demospongiae. Some of them are highly cytotoxic against tumor cells [4]. Since the first isolation of three yellow highly conjugated isomalabaricane-type triterpenoids from the marine sponge *Jaspis stellifera* in 1981 [5] more than 130 isomalabaricanes and related natural products have been reported from the abovementioned sponge genera. It was noticed that *Stelletta* metabolites are quite different depending on the collection. Indeed, isomalabaricane triterpenoids were mainly found as very complex mixtures in tropical sponge samples, while boreal and cold-water sponges contain mostly alkaloids and lipids. From a chemo-ecological point of view, this indicates that studied sponges are able to produce different types of secondary metabolites in order to adapt to the various living conditions [6]. In confirmation, our attempt to find isomalabaricanes in a cold-water *Stelletta* spp., collected in 2019 in the Sea of Okhotsk, was unsuccessful, as the characteristic yellow pigments were not detected by thin layer chromatography in the extracts of these sponges.

Additionally, in result of the chemical investigation of the sponge *Stelletta tenuis*, Li et al. identified two naturally occurring α-pyrones, namely gibepyrones C and F, along with three isomalabaricane-type triterpenoids [7]. These α-pyrones were supposed to be the oxidation products of the co-occurring stellettins [6]. Gibepyrone F had previously been isolated from the fungal plant pathogen *Gibberella fujikuroi* [8], as well as from the sponge *Jaspis stellifera* [9]. These findings allow to presume that symbiotic microorganisms in the corresponding sponges are involved in the generation of some metabolites.

Diverse isomalabaricane-type *nor*-terpenoids, containing less than 30 carbon atoms in their skeleton systems, have been found together with isomalabaricanes several times [10,11,12]. Their presence could be explained either by oxidative degradation of C_30_ metabolites or by precursor role of *nor*-terpenoids in the biosynthesis of these compounds [12,13]. However, the biogenesis of isomalabaricane compounds in sponges remains to be mysterious so far.

Recently, we have reported the isolation of two isomalabaricane-type *nor*-terpenoids, cyclobutastellettolides A and B, and series of known isomalabaricanes from a *Stelletta* sp. [14] We suppose that new data on structural variety of isomalabaricane derivatives supported with strong evidence on stereochemistry could someday shed light on their origin.

In the present work, an investigation of the chemical components of a *Stelletta* sp. from Vietnamese waters was continued. Herein, we report the isolation and structural elucidation of six new compounds **1**–**6** and known globostelletin N [15].

## 2. Results and Discussion

**Figure 1:**
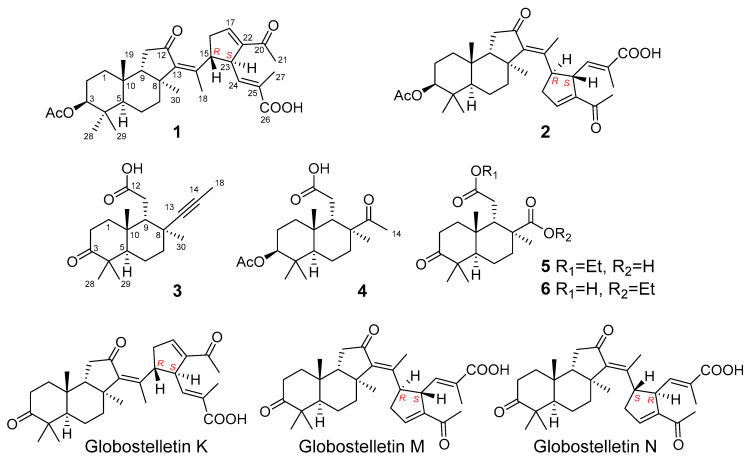
Structures of compounds **1**–**6** and globostelletins K, M, and N.

The frozen sample of a marine sponge *Stelletta* sp. was finely chopped and extracted with EtOH, then the extract was concentrated under reduced pressure and subjected to Sephadex LH-20 and silica gel column chromatography followed by normal- and reversed-phase HPLC procedures (Figure S73) to afford new stellettins Q-V **1**–**6** together with known globostelletin N [15] (Figure 1).

Stellettin Q (**1**) was isolated as a yellow oil with molecular formula C_32_H_44_O_6_ deduced by HRESIMS (Figure S3). The NMR data of **1** (Table 1; Figures S4 and S5) were closely related to the spectral characteristics of isomalabaricane globostelletin K (Figure 1, Figures S55 and S56) initially found in the marine sponge *Rabdastrella globostellata* [15] and also co-isolated from the studied *Stelletta* sp. [14].

**Figure 2:**
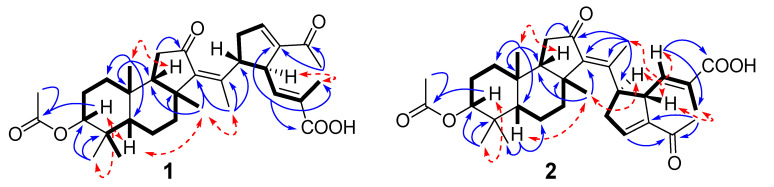
Selected COSY (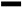), HMBC (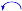) and ROESY (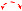) correlations of **1** and **2**.

The detailed analysis of 2D spectra (COSY, HSQC, HMBC etc.) of **1** supported the main structure (Figure 2 and Figures S6–S9). The signals of methyl group at *δ*_H_ 2.06, s; *δ*_C_ 21.2 and acetate carbonyl at *δ*_C_ 171.0, together with the HMBC correlation of axial proton H-3 at *δ*_H_ 4.54, dd (11.7, 5.2) to that carbonyl, revealed the O-acetyl substitution at C-3 in the ring A. Moreover, the signal of C-3 at *δ*_C_ 80.8 instead of ketone signal at *δ*_C_ 219.2 in ^13^C NMR spectrum of globostelletin K also demonstrated the 3-acetoxy-tricyclic core in **1**, while the 3*β*-orientation of acetoxy group was confirmed by strong correlations of H-3/H-5 and CH_3_-28 observed in the ROESY spectrum. The 13*Z* geometry in **1** was in agreement with the signal of CH_3_-18 at *δ*_H_ 1.79, s and its ROESY correlation with CH_3_-30. As well as *E* configuration of 24(25)-double bond was found from the *W*-path COSY correlation of protons H-24/CH_3_-27.

**Figure 3:**
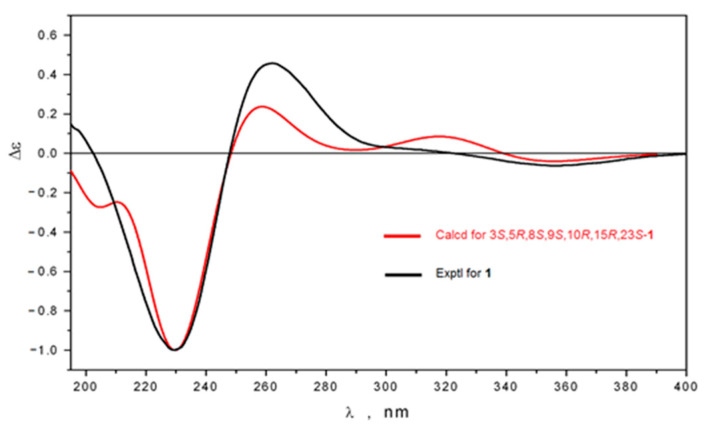
Comparison of experimental and theoretical ECD spectra of stellettin Q (**1**).

The ^1^H- and ^13^C-NMR signals of the side chain of **1** as well as the form of ECD curve (Figure S10) were analogous to those of globostelletin K [15] (Figure S57) suggesting the same stereochemistry of the side chain. This assignment was in a good agreement with the computational ECD results performed using density functional theory (DFT) with the nonlocal exchange-correlation functional B3LYP [16], the polarization continuum model (PCM) [17] and split-valence basis sets 6-31G(d), implemented in the Gaussian 16 package of programs [18] (Figure S66). The 15*R*,23*S* absolute configuration, providing 13*Z*,24*E* geometry and *trans−syn−trans*-fused tricyclic moiety with 3*β*-oriented acetoxy group fully satisfies the similarity of the experimental and theoretical ECD spectra of **1** (Figure 3). In detailes, statistically avereged curve (Figure 3) follows the shape of the experimental one, although even more close coincidence was indicated for theoretically less probable conformer (Figure S67). In addition, we could conclude that the presence of 3-acetoxy or 3-oxo functions in the structures of the corresponding compounds insignificantly affects the shape of their ECD curves. According to obtained new data we pose the same 15*R*,23*S* stereochemistry for globostelletin K (Figure S69).

Stellettin R (**2**) has a molecular formula of C_32_H_44_O_6_ as it was established on the basis of HRESIMS (Figure S11). Spectral data (Table 1, Figure 2, Figures S12 and S13) were consistent with known globostelletin M [15] (Figure 1, Figures S59 and S60) possessing an isomalabaricane core connected with 13*E* double bond (CH_3_-18: *δ*_H_ 2.06, s). However, like stellettin Q, it contains 3*β*–acetoxy group (*δ*_H_ 4.53, dd (11.6, 5.2); *δ*_C_ 80.7; *δ*_H_ 2.05, s; *δ*_C_ 170.9; 21.2). Concerning to the relative configuration of the cyclopentene unit in the side chain of **2**, the ROESY cross-peaks between H-15/H-24 and H-23/CH_3_-18 ascertained a *trans*-relationship of the vicinal protons H-15 and H-23. Careful examination of the chemical shifts for CH-15 (*δ*_H_ 3.22, dt (9.2, 6.0); *δ*_C_ 48.0) and CH-23 (*δ*_H_ 3.95, m; *δ*_C_ 48.0) showed the values similar to those of globostelletin M and differed from globostelletin N (Figure 1, Figures S63 and S64) isolated by Li et al. [15] and co-isolated by us. Moreover, the ECD spectrum of **2** (Figure S18) displayed the same curve and peaks as those published for globostelletin M (Figure S61).

**Figure 4:**
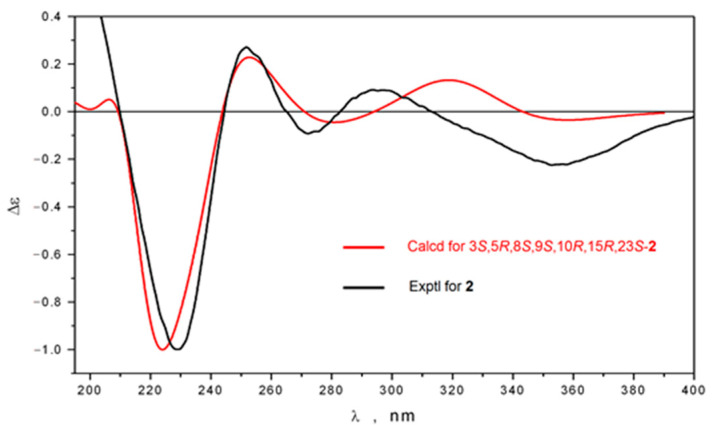
Comparison of experimental and theoretical ECD spectra of stellettin R (**2**).

However, structure modeling as well as calculation of ECD spectra for possible stereoisomers of **2** demonstrated a good agreement between experimental and theoretical spectra for 15*R*,23*S* absolute configuration (Figure 4) quite differ from 15*S*,23*S* reported for globostelletin M [15]. This inconsistence encouraged us to re-investigate the stereochemistry of co-isolated globostelletins M and N. We have obtained NMR and ECD spectra of the both compounds (Figures S59–S65) and they were identical to those provided as supplementary data by Li et al. [15]. At the same time, our computational results suggested globostelletin M to possess the same 15*R*,23*S* absolute configuration (Figure S70) of cyclopentene unit as **2**, while globostelletin N has 15*S*,23*R* stereochemistry (Figure S71). Based on the data we believe that previously published research comprises some inaccuracies and the stereochemistry of these centres in corresponding isomalabaricanes should be revised. It was noted that the isomalabaricane-type terpenoids undergo a photoisomerization of the side chain 13-double bond during the isolation and storage [19,20]. We consider compounds **1** and **2** to be the 13*Z*/*E* pair of the same 15*R*,23*S* isomer.

**Figure 5:**
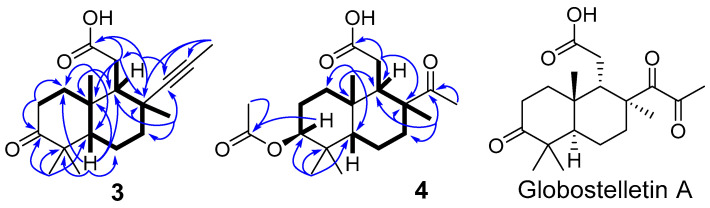
Selected COSY (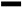) and HMBC (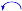) correlations of **3** and **4** and structure of known globostelletin A.

The molecular formula C_19_H_28_O_3_ of stellettin S (**3**) calculated from HRESIMS data (Figure S19) showed **3** to be a rather smaller molecule then classical C_30_-isomalabaricanes, intriguing due to the lack of a significant part in the molecule, when compared with the majority of known isomalabaricanes and their derivatives. The ^13^C- and DEPT NMR spectra (Table 2; Figures S21 and S22) exhibited 19 resonances, including those of carbonyl carbon at *δ*_C_ 216.5 (C-3) and carboxyl carbon at *δ*_C_ 178.8 (C-12) as well as two down-shifted quaternary carbons at *δ*_C_ 88.1 (C-13), 77.8 (C-14). ^1^H- and ^13^C-NMR spectra (Figures S20 and S21) revealed five methyls, two methylene, two methine groups and seven quaternary carbons, suggesting an isoprenoid nature. In the HSQC spectrum (Figure S23) four methyl singlets (*δ*_H_ 1.06, 1.08, 1.25, and 1.62) correlated with carbon signals at *δ*_C_ 21.6 (CH_3_-29), 25.9 (CH_3_-28), 30.8 (CH_3_-30) and 23.3 (CH_3_-19), respectively, while singlet of one more methyl group at *δ*_H_ 1.80 gave a cross-peak with high field signal at *δ*_C_ 3.7 (CH_3_-18). The further inspection of 2D spectra (Figure 5 and Figures S23–S26) revealed the bicyclic framework resembling the core of globostelletin A (Figure 5), isolated from the sponge *Rhabdastrella globostellata* [13].

This was confirmed by the key long-range HMBC correlations from gem-dimethyl group (CH_3_-28 and 29) to C-3, C-4 and C-5; from H-5 to C-1, C-4, C-6, C-9 and C-10; from methyl CH_3_-19 to C-1, C-9 and C-10; from methyl CH_3_-30 to C-7, C-8 and C-9 as well as from the methylene of carboxymethyl group (CH_2_-11) to C-8, C-9, C-10 and carboxyl carbon C-12 (Figure 5 and Figure S24). The empirical formula, besides bicyclic system and two carbonyls, required two additional degrees of unsaturation which were accounted for an acetylenic bond in a short side chain. The NMR signals of two quaternary carbons at *δ*_C_ 88.1 (C-13), 77.8 (C-14) and methyl (CH_3_-18) at *δ*_H_ 1.80, *δ*_C_ 3.7 were finally attributed to the methylacetylenic substituent at C-8, that was confirmed by HMBC correlations from CH_3_-30 to C-8 and C-13, along with that from CH_3_-18 to C-7, C-8, C-9, C-13, C-14 and CH_3_-30. Analogous methylacetylenic substituent was characterized previously with similar chemical shifts in a series of synthetic alkynes [21].

Interestingly, the NMR signal of CH_3_-19 (*δ*_H_ 1.62, s) was notably downfield shifted in comparison with that in a number of isomalabaricanes and their derivatives spectra. We explained it by the joint influence of the methylacetylene and carboxymethyl groups. The quantum chemical calculations (Figure S66) of the chemical shifts for structure **3** confirmed the down-shifted position of the proton signal of CH_3_-19 and afforded its theoretical chemical shift value of *δ*_H_ 1.69 ppm.

**Figure 6:**
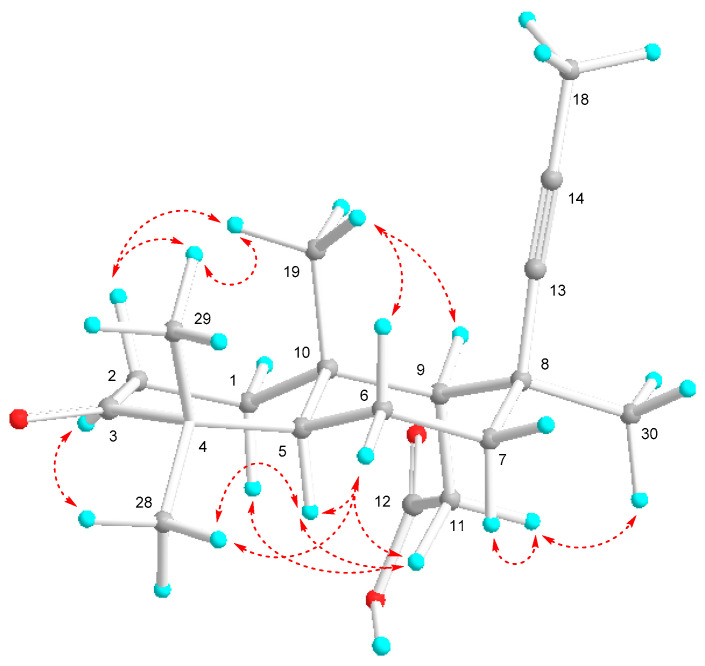
Selected ROESY (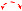) correlations of **3**.

The relative stereochemistry of **3** was determined by ROESY experiment (Figure 6 and Figure S26). A *trans*-fusion of the bicyclic system was shown by key NOE interactions. The correlations between CH_3_-19/CH_3_-29, CH_3_-28/H-5, H-5/H_a_-11, CH_3_-19/H-9, and CH_3_-30/H_b_-11 showed the *β*-orientations of CH_3_-19 and H-9, whereas H-5, CH_2_-11, and CH_3_-30 were *α*-oriented. The chair conformation of the ring B with equatorial positions of H-9 and CH_3_-30 corresponded to the long-range COSY correlation between H-9 and H*_β_*-7 (Figure 5 and Figure S25) together with ROESY correlations H-5/H_a_-11 and H_α_-7/H_b_-11. Taking into consideration the relative stereochemistry of the compound **3** along with above mentioned absolute stereochemistry of the C_30_ congeners **1** and **2** as well as the fact of co-isolation of cyclobutastellettolides A and B [14] with the same absolute configurations we suggested the 5*S,* 8*R,* 9*R,* 10*R* absolute stereochemistry of stellettin S (**3**).

Stellettin T (**4**) with the molecular formula C_20_H_32_O_5_ seemed to be another isomalabaricane-type derivative. The ispection of NMR data (Table 2; Figure 5 and Figures S29–S33) revealed the same type of 9-carboxymethyl substituted bicyclic core as was deduced for compound **3**. It contains 3*β*-acetoxy group, confirmed with the signals of CH-3 (*δ*_H_ 4.44, dd (11.2, 5.1); *δ*_C_ 80.3), methyl of acetate group (*δ*_H_ 2.05, s; *δ*_C_ 21.2) and acetate carbon (*δ*_C_ 170.9). According to the ^13^C NMR spectrum and molecular formula, compound **4** has one carbonyl less side chain then known globostelletin A [13]. Based on this data and HMBC correlations from CH_3_-14 (*δ*_H_ 2.12, s) and CH_3_-30 (*δ*_H_ 1.31, s) to C-13 (*δ*_C_ 213.0), the acetyl was connected with C-8 (*δ*_C_ 52.5). The key ROESY correlations H-3/H-5, H-5/H_a_-11, H-14/CH_3_-19, H-9/CH_3_-19 and H-9/CH_3_-29 (Figure S34) suggested configurations at C-5, C-8, C-9 and C-10 identical to those of co-isolated isomalabaricanes.

**Figure 7:**
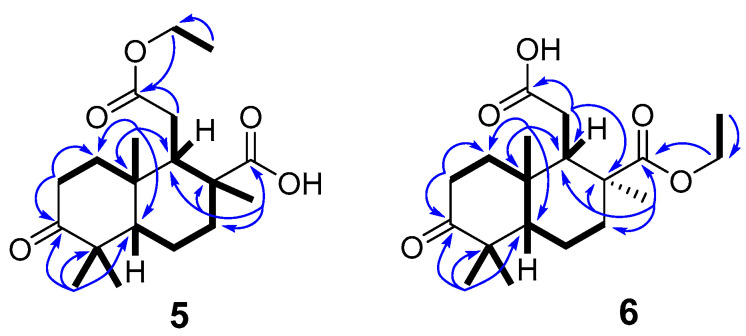
Selected COSY (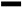) and HMBC (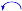) correlations of **5** and **6**.

The structures of stellettins U (**5**) and V (**6**) corresponded to the same C_19_H_30_O_5_ molecular formula deduced from HRESIMS (Figures S36 and S45). In comparison with co-isolated metabolites, the spectral data of compounds **5** and **6** revealed bicyclic core with keto group at C-3, gem-dimethyl group at C-4 and two angular methyls at C-8 and C-10 (Table 2). Additionally, ^1^H- and ^13^C-NMR spectra of compound **5** (Figures S37 and S38) demonstrated signals of two carbonyls (*δ*_C_ 173.7 and 183.8) and one ethoxy group (*δ*_H_ 4.15, q (7.1); *δ*_C_ 60.8 and *δ*_H_ 1.26, t (7.1); *δ*_C_ 14.1). HMBC experiment (Figure 7 and Figure S41) allowed to place the carboxy group at C-8 and ethyl ester at C-11 on the basis of congruous correlations from methylenes –CH_2_-CH_3_ (*δ*_H_ 4.15, q (7.1), 2H) and CH_2_-11 (*δ*_H_ 2.41, dd (17.7, 5.3) and 2.30, m) to carboxyl C-12 (*δ*_C_. 173.7) and also from methyl CH_3_-30 (*δ*_H_ 1.17, s) to carboxyl C-13 (*δ*_C_ 183.8). The relative stereochemistry of **5** was determined by ROESY spectral analysis (Figure S43). Correlation between H-9 (*δ*_H_ 2.78, br t (5.0)) and CH_3_-19 (*δ*_H_ 1.24, s) indicated their *β*-orientation. Meanwhile, a ROESY correlation between H-5 (*δ*_H_ 1.40, dd (12.6, 3.0))/H_a_-11 (*δ*_H_ 2.41, dd (17.7, 5.3)) and H_b_-11 (*δ*_H_ 2.30, m)/CH_3_-30 (*δ*_H_ 1.17, s) confirmed the *α*-orientation of H-5, -CH_2_-COOEt and CH_3_-30. The above-mentioned results were in agreement with the spatial structure of isomalabaricane derivatives.

Compound **6** was an isomer of compound **5**, differed by the NMR signals (Figures S46 and S47) of carboxylic carbons (*δ*_C_ 177.6 and 178.0), methyl C-19 (*δ*_H_ 1.14, s), methylene CH_2_-11 (*δ*_H_ 2.48, dd (18.1, 5.4) and 2.38, dd (18.1, 4.8)) and ethoxy group (*δ*_H_ 4.23, dq (10.9, 7.1); 4.13, dq (10.9, 7.1) and 1.31, t (7.1)). The key HMBC correlations (Figure 7) satisfied the proposed structure of **6**. However, since the values of carboxyl carbons shifts for **6** are close, distinguishing their correlations and direct ester positioning without data for isomer **5** brought some uncertainty. To avoid future difficulties with structurally related esters we calculated carbon chemical shift values for two isomers **5** and **6** (Figure S66). It was shown, that theoretical *δ*_C_ C-13 (**5**) = 192.7 and *δ*_C_ C-12 (**5**) = 182.4 gave the Δ*δ*_C(13-12)_ = 10.3 ppm close to experimental value Δ*δ*_C(13-12)_ = 10.1 ppm, while theoretical and experimental Δ*δ*_C(13-12)_ for compound **6** were of 0.4 ppm (clcd *δ*_C_ C-13 (**6**) = 183.2, *δ*_C_ C-12 (**6**) = 182.8).

ROESY correlations of **6** supported the relative stereochemistry similarly to that of compound **5**. In fact, we detected expected NOE interactions H-9 (δ_H_ 2.74, br t (4.6))/CH_3_-19 (δ_H_ 1.14, s); H-5 (δ_H_ 1.39, dd (12.7; 3.0))/H_a_-11 (δ_H_ 2.48, dd (18.1, 5.4)) and H_b_-11 (δ_H_ 2.38, dd (18.1, 4.8))/CH_3_-30 (δ_H_ 1.12, s). Therefore, derivatives **5** and **6** possess the same stereochemistry as other co-isolated isomalabaricanes. Although compounds **5** and **6** are rather artificial products derived during EtOH extraction, the isolated pair of esters allowed to reliably establish the position of the ether group based on the chemical shifts of C-12 and C-13.

Both compounds were supposed to be the half-ester derivatives of the hypothetical dicarboxylic acid. The anhydrous form of the acid was reported by Ravi et al. [5] as a product of ozonolysis of isomalabaricane precursor [22,23]. Moreover, Ravi et al. obtained dimethyl and monomethyl esters of the acid and did not point the place of esterification in the case of the latter.

Among isolated new compounds **1**–**6**, we find stellettin S (**3**) the most intriguing, since occurrences of acetylene-containing isoprenoids are rare and not so far reported in the isomalabaricane series. To date, several biosynthetic pathways leading to the alkyne formation in natural products has been supported with identified and characterized gene clusters. In the first case, acetylenases, a special family of desaturases, catalyze the dehydrogenation of olefinic bonds in unsaturated fatty acids to afford acetylenic functionalities [24,25]. Next, acetylenases are also used to form the terminal alkyne in polyketides [26]. One more biosynthetic route results in a terminal alkyne formation in acetylenic amino acids and involves consequent transformations by halogenase BesD, oxidase BesC and lyase BesB [27]. Finally, two recent papers describe the molecular basis for the formation of alkyne moiety in acetylenic prenyl chains occurring in a number of meroterpenoids [28,29]. The abovementioned reports highlight hot trends in a scientific search for enzymatic machineries leading to the biologically significant and synthetically applicable acetylene bond in natural compounds. We believe that isolation of the new terpenoidal alkyne **3** could inspire further investigations of the *Stelletta* spp. sponges and associated microorganisms through genome mining.

According to obtained new data we also report the correction in stereochemistry of two asymmetric centers in globostelletins M (Figure S70) and N (Figure S71). Really, their ECD and NMR spectra in comparison with those of globostelletin K and stellettins Q and R (Figures S59–S71) clearly show rather 15*R*,23*S* configuration for globostelletin M instead of previously reported 15*S*,23*S* [15] as well as 15*S*,23*R* stereochemistry for globostelletin N instead of 15*R*,23*R* [15].

## 3. Materials and Methods

### 3.1. General Experimental Procedures

Optical rotations were measured on Perkin-Elmer 343 digital polarimeter (Perkin Elmer, Waltham, MA, USA). UV-spectra were registered on a Shimadzu UV-1601PC spectrophotometer (Shimadzu Corporation, Kyoto, Japan). ECD spectra were obtained on a Chirascan plus instrument (Applied Photophysics Ltd., Leatherhead, UK). ^1^H-NMR (500.13 MHz, 700.13 MHz) and ^13^C-NMR (125.75 MHz, 176.04 MHz) spectra were recorded in CDCl_3_ on Bruker Avance III HD 500 and Bruker Avance III 700 spectrometers (Bruker BioSpin, Bremen, Germany). The ^1^H- and ^13^C-NMR chemical shifts were referenced to the solvent peaks at δ_H_ 7.26 and δ_C_ 77.0 for CDCl_3_. HRESIMS analyses were performed using a Bruker Impact II Q-TOF mass spectrometer (Bruker). The operating parameters for ESI were as follows: a capillary voltage of 3.5 kV, nebulization witH-Nitrogen at 0.8 bar, dry gas flow of 7 L/min at a temperature of 200 °C. The mass spectra were recorded within *m/z* mass range of 100–1500. The instrument was operated using the otofControl (ver. 4.1, Bruker Daltonics) and data were analyzed using the DataAnalysis Software (ver. 4.4, Bruker Daltonics). Column chromatography was performed on Sephadex LH-20 (25–100 µm, Pharmacia Fine Chemicals AB, Uppsala, Sweden), silica gel (KSK, 50–160 mesh, Sorbfil, Krasnodar, Russia) and YMC ODS-A (12 nm, S-75 um, YMC Co., Ishikawa, Japan). HPLC were carried out using an Agilent 1100 Series chromatograph equipped with a differential refractometer (Agilent Technologies, Santa Clara, CA, USA). The reversed-phase columns YMC-Pack ODS-A (YMC Co., Ishikawa, Japan, 10 mm × 250 mm, 5 µm and 4.6 mm × 250 mm, 5 µm) and Discovery HS F5-5 (SUPELCO Analytical, Bellfonte, PA, USA, 10 mm × 250 mm, 5 µm) were used for HPLC. Yields are based on dry weight (212.1 g) of the sponge sample.

### 3.2. Animal Material

The *Stelletta* sp. sponge sample (wet weight 1.3 kg) was collected by SCUBA diving at the depth of 7–12 m near Cham Island (15°54.3′ N, 108°31.9′ E) in the Vietnamese waters of the South China Sea during the 038-th cruise of R/V “Academik Oparin” in May 2010. The species was identified and described [14] by Dr. Boris B. Grebnev from G. B. Elyakov Pacific Institute of Bioorganic Chemistry, FEB RAS (PIBOC, Vladivostok, Russia). A voucher specimen (PIBOC O38-301) has been deposited at the collection of marine invertebrates in PIBOC.

### 3.3. Extraction and Isolation

The frozen sponge was chopped and extracted with EtOH (1.7 L × 3) (Figure S73). The EtOH soluble materials (52.5 g) were concentrated, dissolved in distilled H_2_O (100 mL) and partitioned in turn with EtOAc (100 mL × 3). The EtOAc extracts were concentrated to a dark brown gum (15.7 g) that was further separated on a Sephadex LH-20 column (2 × 95 cm, CHCl_3_/EtOH, 1:1) to yield three fractions. Fraction 2 (10.2 g) was separated into nine subfractions using step-wise gradient silica gel column chromatography (4 × 15 cm, CHCl_3_→EtOH). Subfraction 2.4 (3.4 g) eluted with CHCl_3_/EtOH (80:1–10:1) was subjected to a silica gel column (4 × 15 cm, CHCl_3_/EtOH, 100:1→10:1) to obtain four subfractions. The fourth subfraction 2.4.4 (255.5 mg) was subjected to reversed-phase HPLC (YMC-Pack ODS-A, 70% EtOH) to give four subsubfractions (2.4.4.1-4) that were subjected to rechromatography. The HPLC fractionation of 2.4.4.1 (YMC-Pack ODS-A, 60% EtOH) gave cyclobutastellettolide A (7.7 mg, 0.004%), mixtures of globostelletins E+F (~2:1; 4.0 mg, 0.002%), K (3.4 mg, 0.002%), and M (1.7 mg, 0.002%), that were purified by HPLC procedures (Discovery HS F5-5, 60% EtOH), as it was reported previously [14]. One more component of this subsubfraction was purified (Discovery HS F5-5, 60% EtOH) to yield stellettin V (**6**, 2.6 mg, 0.001%). The subsubfraction 2.4.4.2, subjected to HPLC (Discovery HS F5-5, 70% EtOH) gave cyclobutastellettolide B [14] (1.6 mg, 0.004%) and stellettin T (**4**, 1.2 mg, 0.0006%), purified by HPLC (Discovery HS F5-5, 70% EtOH). The subsubfraction 2.4.4.3 contained cyclobutastellettolide B (1.4 mg) and stellettin Q (**1**, 0.9 mg, 0.0008%) isolated using reversed-phase HPLC (Discovery HS F5-5, 70% EtOH). The third subfraction 2.4.3 (641.5 mg) was divided four times (~160 mg × 4) using reversed-phase column chromatography (1 × 5 cm, YMC-Pack ODS-A, 50% EtOH and 100% EtOH) to yeild two subsubfractions. The subsubfraction eluted with 50% EtOH was separated by HPLC (YMC-Pack ODS-A, 70% EtOH) to afford a number of compounds and mixes for further purification. Then, stellettin U (**5**, 10.3 mg, 0.005%) as well as globostelletins N (9.2 mg, 0.004%) and M (1.5 mg) were isolated by HPLC (Discovery HS F5-5) in 80% MeOH. The HPLC procedures (Discovery HS F5-5) in 80% EtOH were used to obtain individual stellettins R (**2**, 2.1 mg, 0.001%), S (**3**, 6.9 mg, 0.003%) and portion of stellettin Q (**1**, 0.9 mg) that was purified by HPLC (Discovery HS F5-5) rechromatography in 65% CH_3_CN. Finally, one more portion of cyclobutastellettolide B (5.0 mg) was obtained from the subfraction by HPLC (Discovery HS F5-5) in 85% MeOH.

### 3.4. Compound Characteristics

Stellettin Q (**1**): Yellow oil; [*α*]_D_^25^–65.0 (*c* 0.1, CHCl_3_); ECD (*c* 8.6 × 10^−^^4^ M, EtOH) *λ*_max_ (Δ*ε*) 195 (4.15), 229 (−27.92), 262 (12.78), 355 (−1.75) nm; ^1^H- and ^13^C-NMR data (CDCl_3_), Table 1; HRESIMS *m/z* 523.3065 [M–H]^−^ (calcd for C_32_H_43_O_6_ 523.3065).

Stellettin R (**2**): Yellow oil; [*α*]_D_^25^–24.0 (*c* 0.2, CHCl_3_); ECD (*c* 1.3 × 10^−^^3^ M, EtOH) *λ*_max_ (Δ*ε*) 195 (4.06), 229 (−5.33), 252 (1.46), 273 (−0.48), 295 (0.47), 353 (−1.20) nm; ^1^H- and ^13^C- NMR data (CDCl_3_), Table 1; HRESIMS *m/z* 523.3068 [M–H]^−^ (calcd for C_32_H_43_O_6_ 523.3065).

Stellettin S (**3**): Slightly yellow oil; [*α*]_D_^25^ + 31.5 (*c* 0.2, CHCl_3_); ECD (*c* 5.3 × 10^−^^3^ M, EtOH) *λ*_max_ (Δ*ε*) 289 (0.34), 321 (−0.14) nm; ^1^H- and ^13^C-NMR data (CDCl_3_), Table 2; HRESIMS *m/z* 303.1966 [M–H]^−^ (calcd for C_19_H_27_O_3_ 303.1966).

Stellettin T (**4**): Slightly yellow oil; [*α*]_D_^25^–22.0 (*c* 0.1, CHCl_3_); ECD (*c* 3.4 × 10^−^^3^ M, EtOH) *λ*_max_ (Δ*ε*) 208 (−0.98), 249 (0.58), 280 (−0.27), 321 (−0.29) nm; ^1^H- and ^13^C-NMR data (CDCl_3_), Table 2; HRESIMS *m/z* 351.2176 [M–H]^−^ (calcd for C_20_H_31_O_5_ 351.2177).

Stellettin U (**5**): Slightly yellow oil; [*α*]_D_^25^ 0.0 (*c* 0.2, CHCl_3_); ECD (*c* 5.7 × 10^−^^3^ M, EtOH) *λ*_max_ (Δ*ε*) 197 (−0.73), 232 (0.27), 261 (0.45), 295 (0.01), 321 (−0.34) nm; ^1^H- and ^13^C-NMR data (CDCl_3_), Table 2; HRESIMS *m/z* 337.2022 [M–H]^−^ (calcd for C_19_H_29_O_5_ 337.2020).

Stellettin V (**6**): Slightly yellow oil; [*α*]_D_^25^ + 32.9 (*c* 0.17, CHCl_3_); ECD (*c* 7.7 × 10^−^^3^ M, EtOH) *λ*_max_ (Δ*ε*) 210 (−0.04), 220 (0.08), 238 (−0.23), 269 (0.38), 291 (0.26), 330 (0.13), 357 (−0.09) nm; ^1^H- and ^13^C-NMR data (CDCl_3_), Table 2; HRESIMS *m/z* 337.2025 [M–H]^−^ (calcd for C_19_H_29_O_5_ 337.2020).

Globostelletin N (Figure 1): Slightly yellow oil; [*α*]_D_^25^ + 17.8 (*c* 0.23, MeOH); ECD (*c* 1.2 × 10^−3^ M, EtOH) *λ*_max_ (Δ*ε*) 196 (−4.18), 228 (9.44), 256 (−1.72), 289 (0.97), 340 (−1.97) nm; ^1^H- and ^13^C-NMR spectra (CDCl_3_) corresponded to previously reported data [15] (Figures S62–S64); HRESIMS *m/z* 479.2808 [M–H]^−^ (calcd for C_30_H_39_O_5_ 479.2803).

## 4. Conclusions

To summarize, the present report describes the isolation and structural elucidation of six metabolites **1**–**6** from a tropical marine sponge belonging the genus *Stelletta*. A combination of NMR methods, supported with computational quantum-chemical modeling allowed us to establish the structures and absolute stereochemistry of two isomalabaricanes **1** and **2**, while the structures and configurations of four isomalabaricane-derived terpenoids **3**–**6** were suggested on the basis of spectral data and biogenetic considerations. Stellettin S (**3**) represents the first acetylene-containing isomalabaricane-related compound. Additionally, according to new data the absolute stereochemistry of the C-15 and C-23 asymmetric centers of known globostelletins M and N were corrected.

## Figures and Tables

**Table 1:** ^1^H and ^13^C NMR data of **1** (700 and 176 MHz) and **2** (500 and 126 MHz) in CDCl_3_.

No. ^1^	1		2	
	*δ*_H_ mult (*J* in Hz)	*δ*_C_	*δ*_H_ mult (*J* in Hz)	*δ*_C_
1*α*	1.57, td (13.0, 3.9)	33.1	1.57, m	32.9
1*β*	1.38, m		1.36, m	
2*α*	1.82, m	25.1	1.81, m	25.1
2*β*	1.71, m		1.68, m	
3*α*	4.54, dd (11.7, 5.2)	80.8	4.53, dd (11.6, 5.2)	80.7
4		38.2		38.2
5*α*	1.73, m	46.5	1.75, m	46.4
6*α*	1.70, m	18.2	1.68, m	18.2
6*β*	1.48, m		1.48, m	
7*α*	1.99, m	37.7	2.06, m	38.5
7*β*	2.00, m		1.93, m	
8		44.2		43.8
9*β*	1.75, m	50.3	1.77, m	50.3
10		35.4		35.4
11*α*	2.13, m	36.4	2.15, m	36.4
11*β*	2.13, m		2.15, m	
12		206.6		206.9
13		146.8		146.0
14		147.9		147.5
15	4.75, m	45.0 ^2^	3.22, dt (9.2, 6.0)	48.0
16*α*	2.27, m	37.9	2.52, m	38.4
16*β*	3.02, ddt (19.4, 9.3, 2.5)		2,89, ddt (19.4, 9.2, 2.7)	
17	6.80, br s	144.6	6.78, dd (4.3, 2.5)	143.6
18	1.79, s	16.2 ^2^	2.06, s	15.7
19	1.02, s	22.4	1.01, s	22.4
20		195.6		195.1
21	2.29, s	26.9	2.30, s	27.0
22		146.6		146.7
23	3.86, br t (8.3)	47.5	3.95, m	48.0
24	6.57, br d (10.2)	145.9	6.59, dd (10.6, 1.5)	144.6
25		126.3		127.3
26		171.0		170.7
27	1.88, br s	12.6	1.86, d (1.3)	12.5
28	0.91, s	29.0	0.90, s	29.0
29	0.89, s	16.9	0.88, s	17.0
30	1.29, s	24.1	1.23, s	26.4
OAc	2.06, s	171.021.2	2.05, s	170.921.2

^1^ Assignments were made with the aid of HSQC, HMBC and ROESY data. ^2^ The values were found from HSQC experiment.

**Table 2:** ^1^H and ^13^C NMR data (700 and 176 MHz) of **3**–**6** in CDCl_3_.

No. ^1^	3		4		5		6	
	*δ*_H_ mult (*J* in Hz)	*δ*_C_	*δ*_H_ mult (*J* in Hz)	*δ*_C_	*δ*_H_ mult (*J* in Hz)	*δ*_C_	*δ*_H_ mult (*J* in Hz)	*δ*_C_
1*α*	1.76, m	35.9	1.33, m	34.3	1.78, m	35.5	1.79, m	35.6
1*β*	1.57, ddd (13.3, 6.3, 3.7)		1.28, m		1.57, ddd (13.5, 6.0, 4.0)		1.60, m	
2*α*	2.33, m	34.8	1.72, m	23.3	2.31, m	34,8	2.32, dt (15.4, 4.5)	34.8
2*β*	2.70, ddd (15.6, 12.3, 6.4)		1.68, m		2.65, ddd (15.4, 12.6, 6.1)		2.66, ddd (15.4, 12.6, 6.1)	
3		216.5	α: 4.44, dd (11.2, 5.1)	80.3		216.1		216.0
4		47.7		37.6		47.5		47.5
5*α*	1.33, dd (12.4, 2.7)	47.5	1.01, dd (11.9; 2.7)	46.2	1.40, dd (12.6; 3.0)	46.7	1.39, dd (12.7; 3.0)	46.8
6*α*	1.47, dq (13.6, 3.2)	21.1	1.65, m	17.9	1.46, m	20.4	1.47, m	20.5
6*β*	1.83, qd (13.2, 3.4)		1.47, m		1.79, m		1.78, m	
7*α*	1.25, td (13.2, 3.7)	36.5	1.51, m	30.0	1.11, m	31.2	1.08, m	31.5
7*β*	1.74, m		1.59, m		2.20, br d (15.1)		2.23, br d (14.2)	
8		34.1		52.5		44.5		44.5
9*β*	2.22, t (5.1)	51.2	2.31 br d (8.0)	49.4	2.78 br t (5.0)	48.3	2.74 br t (4.6)	48.3
10		38.5		38.6		38.5		38.4
11a	2.40, dd (18.1, 5.8)	31.1	2.58, dd (18.9, 7.9)	34.1	2.41, dd (17.7, 5.3)	31.1	2.48, dd (18.1, 5.4)	30.5
11b	2.33, dd (17.7, 4.8)		1.70, br d (18.9)		2.30, m		2.38, dd (18.1, 4.8)	
12		178.8		175.2 ^2^		173.7		177.6 ^3^
13		88.1		213.0		183.8		178.0 ^3^
14		77.8	2.12, s	24.7				
15–17								
18	1.80, s	3.7						
19	1.62, s	23.3	1.27, s	24.6	1.24, s	20.8	1.14, s	20.7
20–27								
28	1.08, s	25.9	0.89, s	27.9	1.07, s	25.7	1.07, s	25.7
29	1.06, s	21.6	0.87, s	16.2	1.00, s	21.5	0.99, s	21.5
30	1.25, s	30.8	1.31,s	24.6	1.17, s	27.8	1.12, s	27.7
OAc			2.05, s	170.9 21.2				
OEt					4.15, q (7.1), 2H, 1.26, t (7.1), 3H	60.8 14.1	4.23, dq (10.9, 7.1), H 4.13, dq (10.9, 7.1), H 1.31, t (7.1), 3H	60.6 14.0

^1^ Assignments were made with the aid of HSQC, HMBC and ROESY data. ^2^ The values were found from HMBC experiment. ^3^ These signals could be interchanged.

## Supplementary Materials

The following are available online. Figure S1: article title, authors affiliations and contact information, Figure S2: contents of Supplementary Materials, Figures S3–S10: HRESIMS, ^1^H- and ^13^C-NMR, HSQC, HMBC, COSY, ROESY spectra (CDCl_3_, 700 MHz) and ECD spectrum (EtOH) of stellettin Q (**1**), respectively, Figures S11–S18: HRESIMS, ^1^H- and ^13^C-NMR, HSQC, HMBC, COSY, ROESY spectra (CDCl_3_, 500 MHz) and ECD spectrum (EtOH) of stellettin R (**2**), respectively, Figures S19–S27: HRESIMS, ^1^H- and ^13^C-NMR, DEPT, HSQC, HMBC, COSY, ROESY spectra (CDCl_3_, 700 MHz) and ECD spectrum (EtOH) of stellettin S (**3**), respectively, Figures S28–S35: HRESIMS, ^1^H- and ^13^C-NMR, HSQC, HMBC, COSY, ROESY spectra (CDCl_3_, 700 MHz) and ECD spectrum (EtOH) of stellettin T (**4**), respectively, Figures S36–S44: HRESIMS, ^1^H- and ^13^C-NMR, DEPT, HSQC, HMBC, COSY, ROESY spectra (CDCl_3_, 700 MHz) and ECD spectrum (EtOH) of stellettin U (**5**), respectively, Figures S45–S53: HRESIMS, ^1^H- and ^13^C-NMR, DEPT, HSQC, HMBC, COSY, ROESY spectra (CDCl_3_, 700 MHz) and ECD spectrum (EtOH) of stellettin V (**6**), respectively, Figures S54–S57: HRESIMS, ^1^H- and ^13^C-NMR spectra (CDCl_3_) and ECD spectrum (EtOH) of globostelletin K, respectively, Figures S58–S61: HRESIMS, ^1^H- and ^13^C-NMR spectra (CDCl_3_) and ECD spectrum (EtOH) of globostelletin M, Figures S62–S65: HRESIMS, ^1^H and ^13^C NMR spectra (CDCl_3_) and ECD spectrum (EtOH) of globostelletin N, respectively, Figure S66: theoretical modeling details, Figures S67 and S68: optimized geometries and statistical weights of main and minor conformations of stellettin Q (**1**) and stellettin R (**2**), respectively, Figures S69–S71: the computational ECD results for globostelletins K, M and N, respectively. Figure S72: the computational ECD results calculated for all possible 15,23-stereoisomers of studied globostelletins K, M, N, and new stellettins Q (**1**) and R (**2**), Figure S73: the isolation scheme.

## Sample Availability

Samples of the compounds are available from the authors.
