# Supplementary material for: New Isomalabaricane-Derived Metabolites from a Stelletta sp. Marine Sponge
Source: Molecules. 2021 Jan 28;26(3):678. doi: 10.3390/molecules26030678 (PMC7866221; doi:10.3390/molecules26030678)
Supplement: Supplementary file 1 [file molecules-26-00678-s001.pdf]

S1

Supplementary Materials

New Isomalabaricane-derived Metabolites from a *Stelletta* sp. Marine Sponge

Sophia A. Kolesnikova [1,*], Ekaterina G. Lyakhova [1], Anastasia B. Kozhushnaya [2], Anatoly I. Kalinovsky [1], Dmitrii V. Berdyshev [1], Roman S. Popov [1], and Valentin A. Stonik [1,2]

[1] G.B. Elyakov Pacific Institute of Bioorganic Chemistry, Far Eastern Branch of Russian Academy of Sciences, Pr. 100-let Vladivostoku 159, 690022 Vladivostok, Russia; sovin81@inbox.ru (S.A.K.); elyakhova@inbox.ru (E.G.L.); kaaniw@piboc.dvo.ru (A.I.K.); berdyshev@piboc.dvo.ru (D.V.B.); prs_90@mail.ru (R.S.P.); stonik@piboc.dvo.ru (V.A.S.)

[2] School of Natural Sciences, Far Eastern Federal University, Sukhanova Str. 8, 690000 Vladivostok, Russia; kozhushnaia.ab@mail.ru (A.B.K); stonik@piboc.dvo.ru (V.A.S.)

* **Correspondence:** sovin81@inbox.ru Tel.: +7-423-231-1168

S2 **Contents:**

S3 HRESIMS and MS/MS Spectra (Negative Ion Mode) of Stellettin Q (**1**)
S4 ^{1}H NMR Spectrum of Stellettin Q (**1**) in $CDCl_3$ (700 MHz)
S5 ^{13}C NMR Spectrum of Stellettin Q (**1**) in $CDCl_3$ (700 MHz)
S6 HSQC Spectrum of Stellettin Q (**1**) in $CDCl_3$ (700 MHz)
S7 HMBC Spectrum of Stellettin Q (**1**) in $CDCl_3$ (700 MHz)
S8 COSY Spectrum of Stellettin Q (**1**) in $CDCl_3$ (700 MHz)
S9 ROESY Spectrum of Stellettin Q (**1**) in $CDCl_3$ (700 MHz)
S10 ECD Spectrum of Stellettin Q (**1**) in EtOH
S11 HRESIMS and MS/MS Spectra (Negative Ion Mode) of Stellettin R (**2**)
S12 ^{1}H NMR Spectrum of Stellettin R (**2**) in $CDCl_3$ (500 MHz)
S13 ^{13}C NMR Spectrum of Stellettin R (**2**) in $CDCl_3$ (500 MHz)
S14 HSQC Spectrum of Stellettin R (**2**) in CDCl3 (500 MHz)
S15 HMBC Spectrum of Stellettin R (**2**) in $CDCl_3$ (500 MHz)
S16 COSY Spectrum of Stellettin R (**2**) in $CDCl_3$ (500 MHz)
S17 ROESY Spectrum of Stellettin R (**2**) in $CDCl_3$ (500 MHz)
S18 ECD Spectrum of Stellettin R (**2**) in EtOH
S19 HRESIMS and MS/MS Spectra (Negative Ion Mode) of Stellettin S (**3**)
S20 ^{1}H NMR Spectrum of Stellettin S (**3**) in $CDCl_3$ (700 MHz)
S21 ^{13}C NMR Spectrum of Stellettin S (**3**) in $CDCl_3$ (176 MHz)
S22 DEPT Spectrum of Stellettin S (**3**) in $CDCl_3$ (176 MHz)
S23 HSQC Spectrum of Stellettin S (**3**) in $CDCl_3$ (700 MHz)
S24 HMBC Spectrum of Stellettin S (**3**) in $CDCl_3$ (700 MHz)
S25 COSY Spectrum of Stellettin S (**3**) in $CDCl_3$ (700 MHz)
S26 ROESY Spectrum of Stellettin S (**3**) in $CDCl_3$ (700 MHz)
S27 ECD Spectrum of Stellettin S (**3**) in EtOH
S28 HRESIMS and MS/MS Spectra (Negative Ion Mode) of Stellettin T (**4**)
S29 ^{1}H NMR Spectrum of Stellettin T (**4**) in $CDCl_3$ (700 MHz)
S30 ^{13}C NMR Spectrum of Stellettin T (**4**) in $CDCl_3$ (176 MHz)
S31 HSQC Spectrum of Stellettin T (**4**) in $CDCl_3$ (700 MHz)
S32 HMBC Spectrum of Stellettin T (**4**) in $CDCl_3$ (700 MHz)
S33 COSY Spectrum of Stellettin T (**4**) in $CDCl_3$ (700 MHz)
S34 ROESY Spectrum of Stellettin T (**4**) in $CDCl_3$ (700 MHz)
S35 ECD Spectrum of Stellettin T (**4**) in EtOH
S36 HRESIMS and MS/MS Spectra (Negative Ion Mode) of Stellettin U (**5**)
S37 ^{1}H NMR Spectrum of Stellettin U (**5**) in $CDCl_3$ (700 MHz)

S38 ^{13}C NMR Spectrum of Stellettin U (**5**) in $CDCl_3$ (176 MHz)
S39 DEPT Spectrum of Stellettin U (**5**) in $CDCl_3$ (176 MHz)
S40 HSQC Spectrum of Stellettin U (**5**) in $CDCl_3$ (700 MHz)
S41 HMBC Spectrum of Stellettin U (**5**) in $CDCl_3$ (700 MHz)
S42 COSY Spectrum of Stellettin U (**5**) in $CDCl_3$ (700 MHz)
S43 ROESY Spectrum of Stellettin U (**5**) in $CDCl_3$ (700 MHz)
S44 ECD Spectrum of Stellettin U (**5**) in EtOH
S45 HRESIMS and MS/MS Spectra (Negative Ion Mode) of Stellettin V (**6**)
S46 ^{1}H NMR Spectrum of Stellettin V (**6**) in $CDCl_3$ (700 MHz)
S47 ^{13}C NMR Spectrum of Stellettin V (**6**) in $CDCl_3$ (176 MHz)
S48 DEPT Spectrum of Stellettin V (**6**) in $CDCl_3$ (176 MHz)
S49 HSQC Spectrum of Stellettin V (**6**) in $CDCl_3$ (700 MHz)
S50 HMBC Spectrum of Stellettin V (**6**) in $CDCl_3$ (700 MHz)
S51 COSY Spectrum of Stellettin V (**6**) in $CDCl_3$ (700 MHz)
S52 ROESY Spectrum of Stellettin V (**6**) in $CDCl_3$ (700 MHz)
S53 ECD Spectrum of Stellettin V (**6**) in EtOH
S54 HRESIMS and MS/MS Spectra (Negative Ion Mode) of Globostelletin K from *Stelletta* sp.
S55 ^{1}H NMR Spectra of Globostelletin K in $CDCl_3$
S56 ^{13}C NMR Spectra of Globostelletin K in $CDCl_3$
S57 ECD Spectra of Globostelletin K
S58 HRESIMS and MS/MS Spectra (Negative Ion Mode) of Globostelletin M from *Stelletta* sp.
S59 ^{1}H NMR Spectra of Globostelletin M in $CDCl_3$
S60 ^{13}C NMR Spectra of Globostelletin M in $CDCl_3$
S61 ECD Spectra of Globostelletin M
S62 HRESIMS and MS/MS Spectra (Negative Ion Mode) of Globostelletin N from *Stelletta* sp.
S63 ^{1}H NMR Spectra of Globostelletin N in $CDCl_3$
S64 ^{13}C NMR Spectra of Globostelletin N in $CDCl_3$
S65 ECD Spectra of Globostelletin N
S66 Theoretical Modeling
S67 Optimized geometries and statistical weights of main and minor conformations of Stellettin Q (**1**)
S68 Optimized geometries and statistical weights of main and minor conformations of Stellettin R (**2**)
S69 The computational ECD results for Globostelletin K
S70 The computational ECD results for Globostelletin M
S71 The computational ECD results for Globostelletin N
S72 The computational ECD results calculated for all possible 15,23-stereoisomers of studied Globostelletins K, M, N, and new Stellettins Q (**1**) and R (**2**).
S73 The Isolation Scheme

S3 HRESIMS and MS/MS Spectra (Negative Ion Mode) of Stellettin Q (**1**)

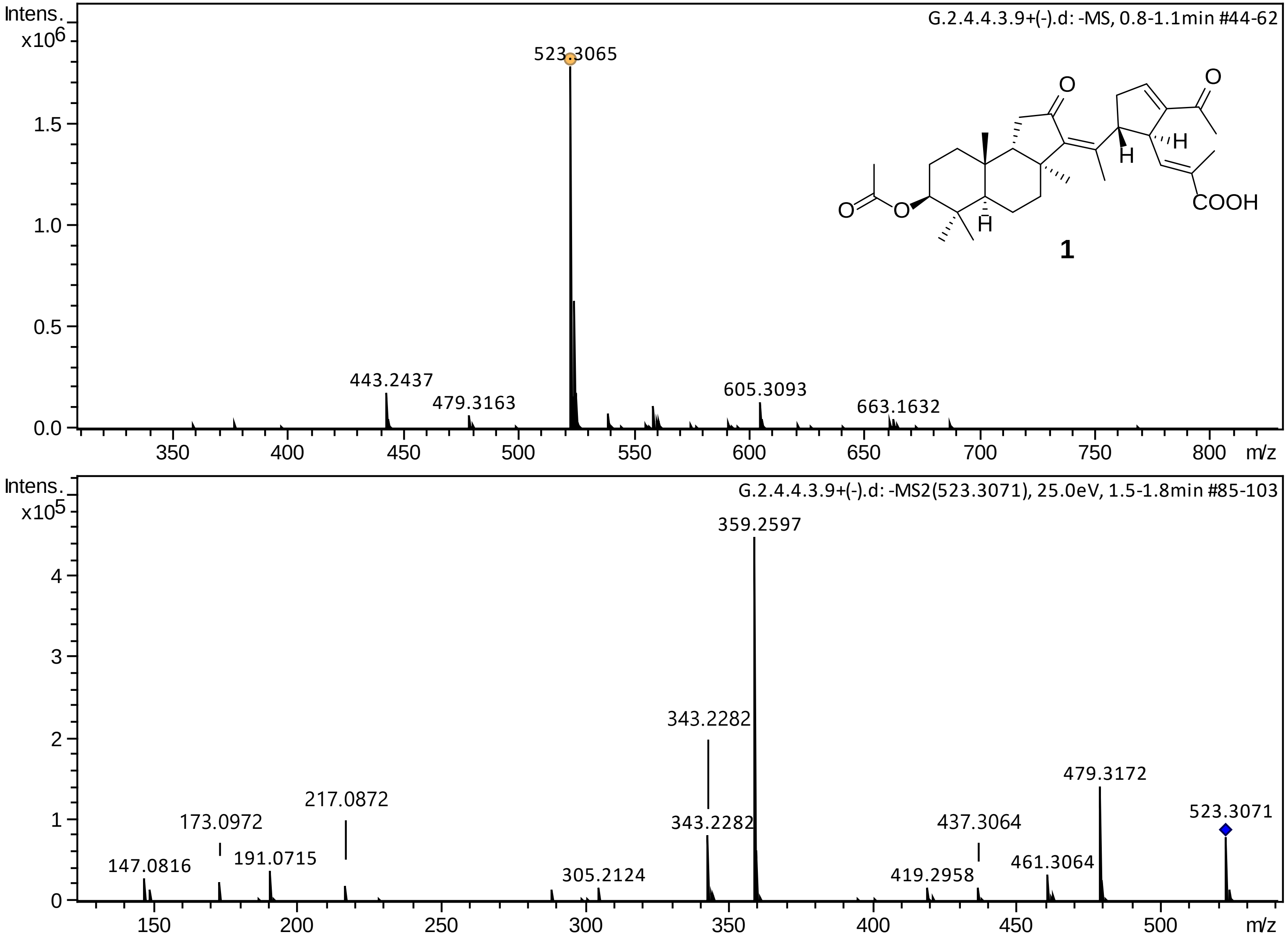

S4 ^{1}H NMR Spectrum of Stellettin Q (**1**) in $CDCl_3$ (700 MHz)

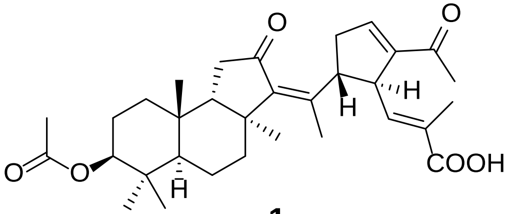

1

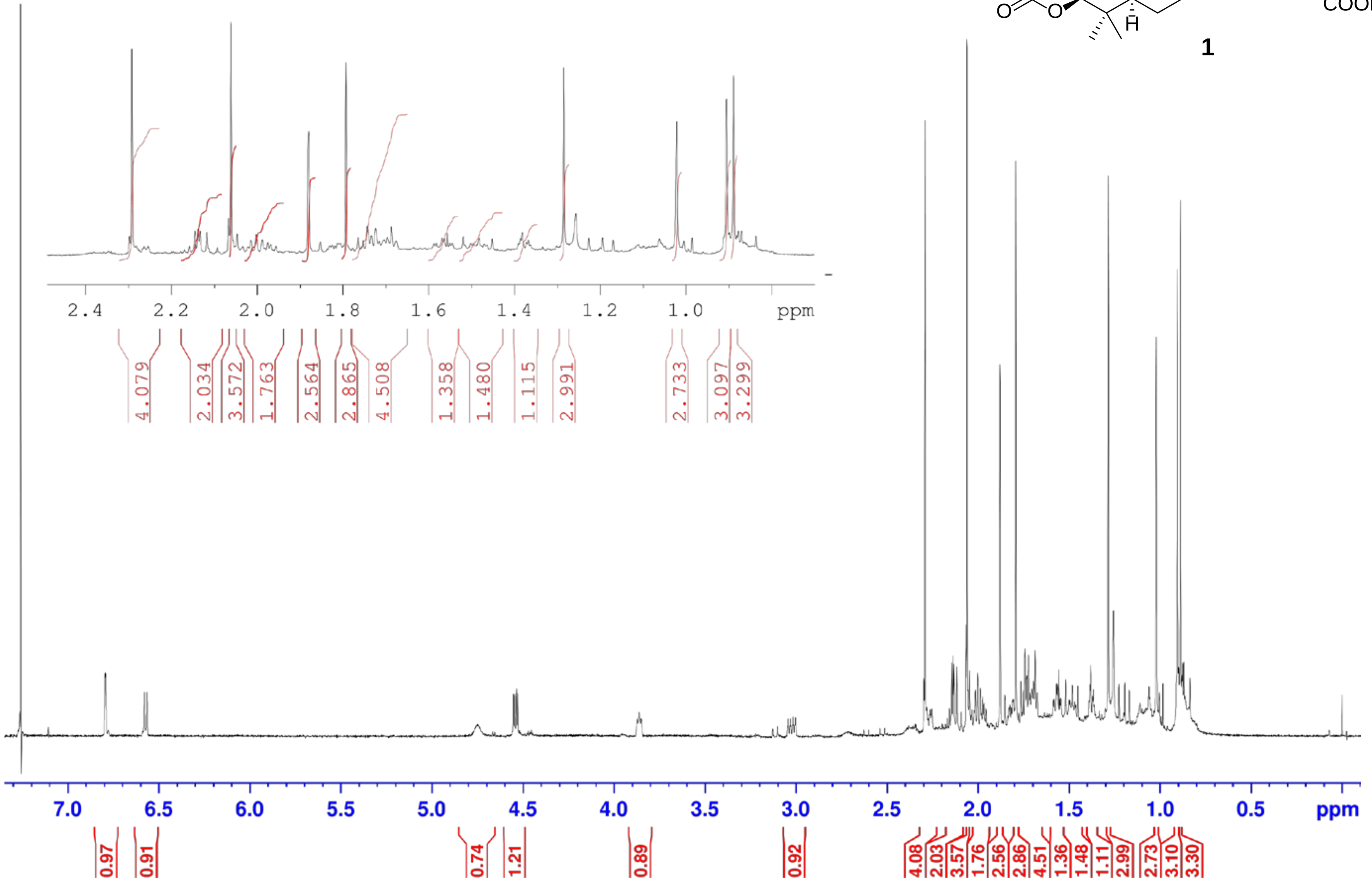

S5 ^{13}C NMR Spectrum of Stellettin Q (**1**) in $CDCl_3$ (700 MHz)

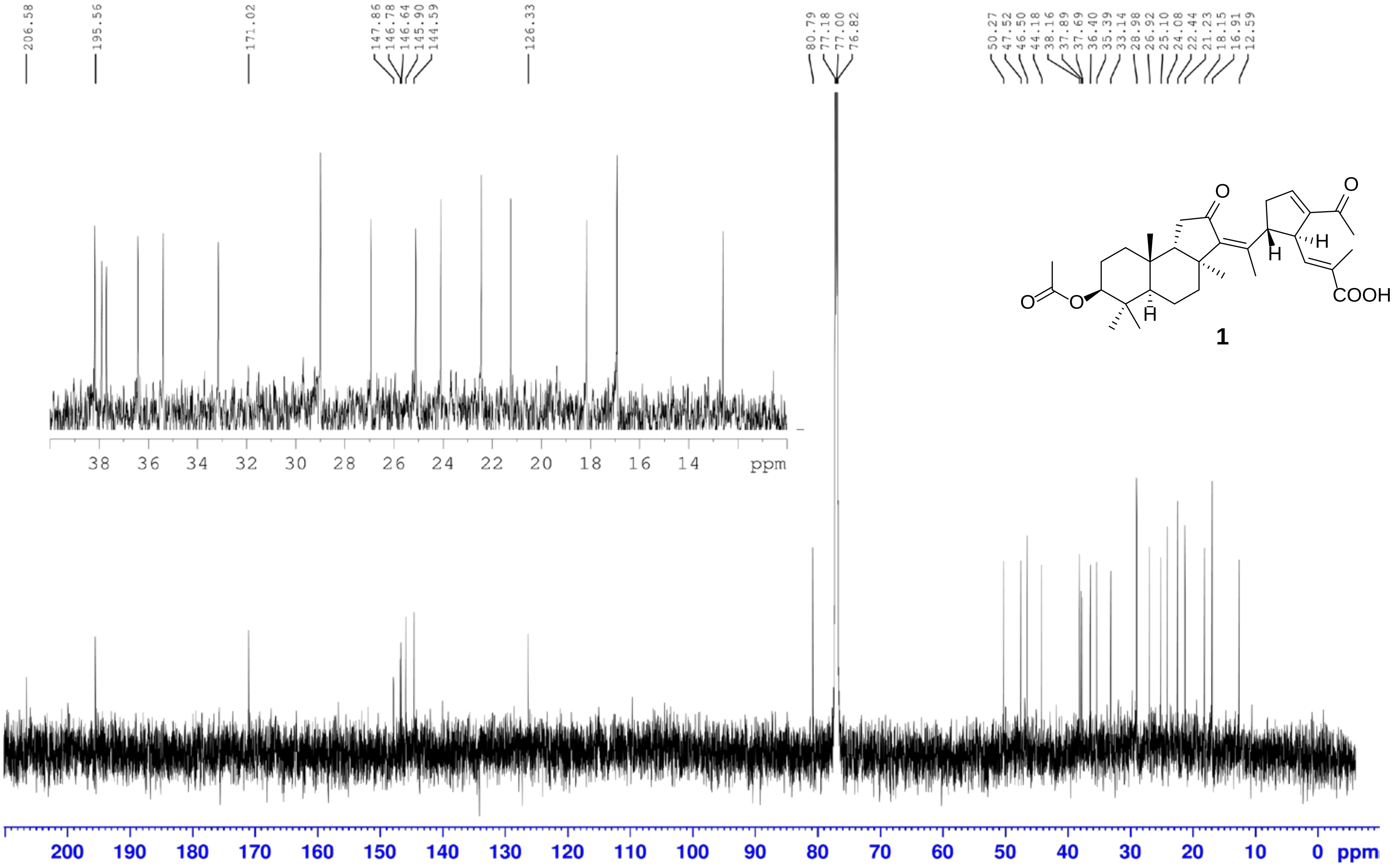

S6 HSQC Spectrum of Stellettin Q (**1**) in $CDCl_3$ (700 MHz)

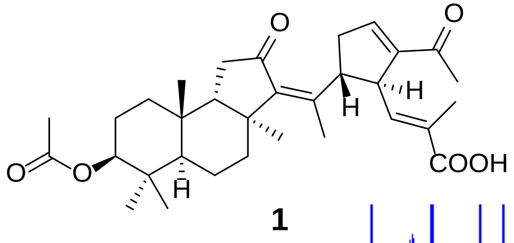

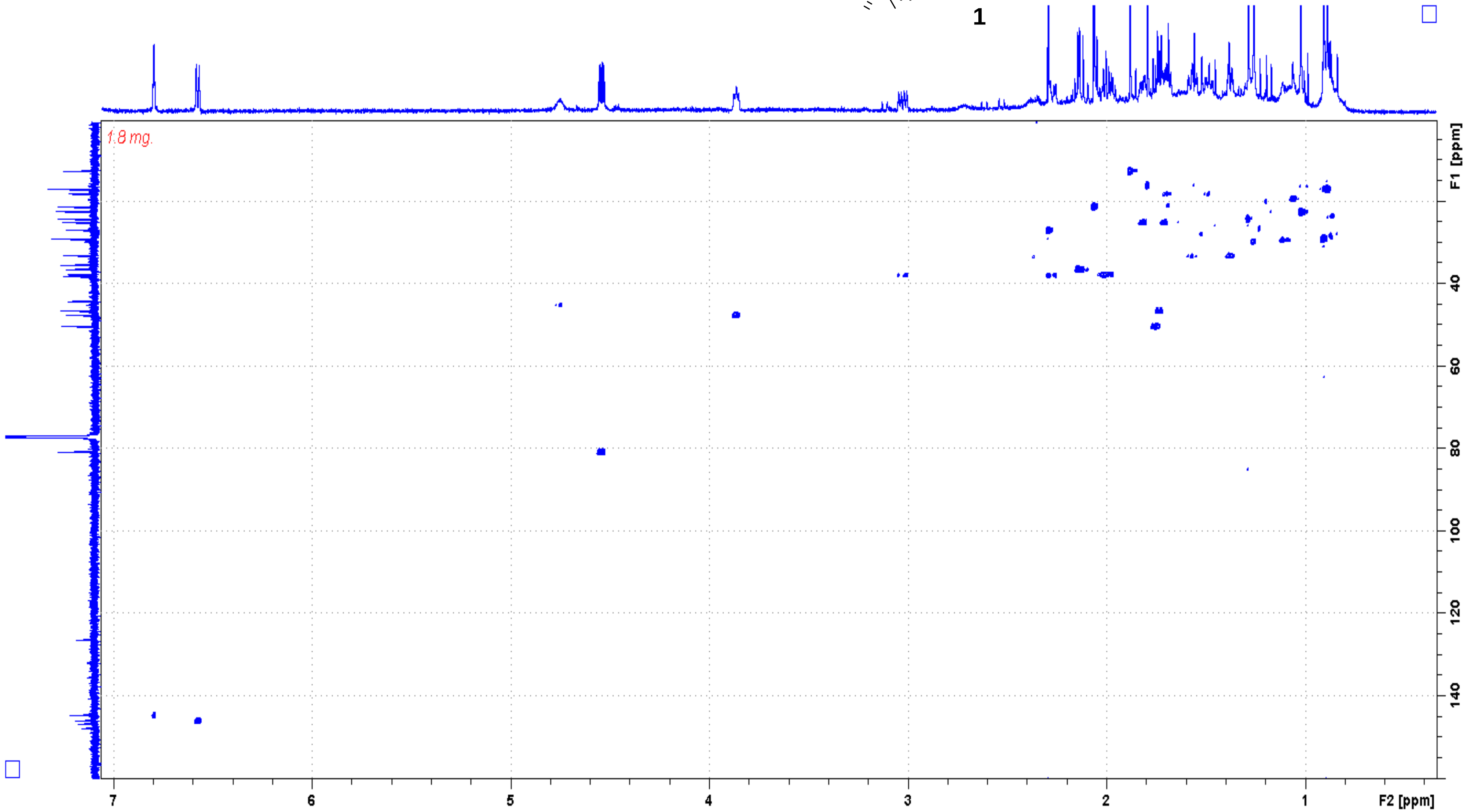

S7 HMBC Spectrum of Stelletin Q (**1**) in $CDCl_3$ (700 MHz)

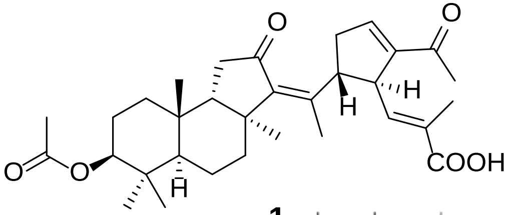

1

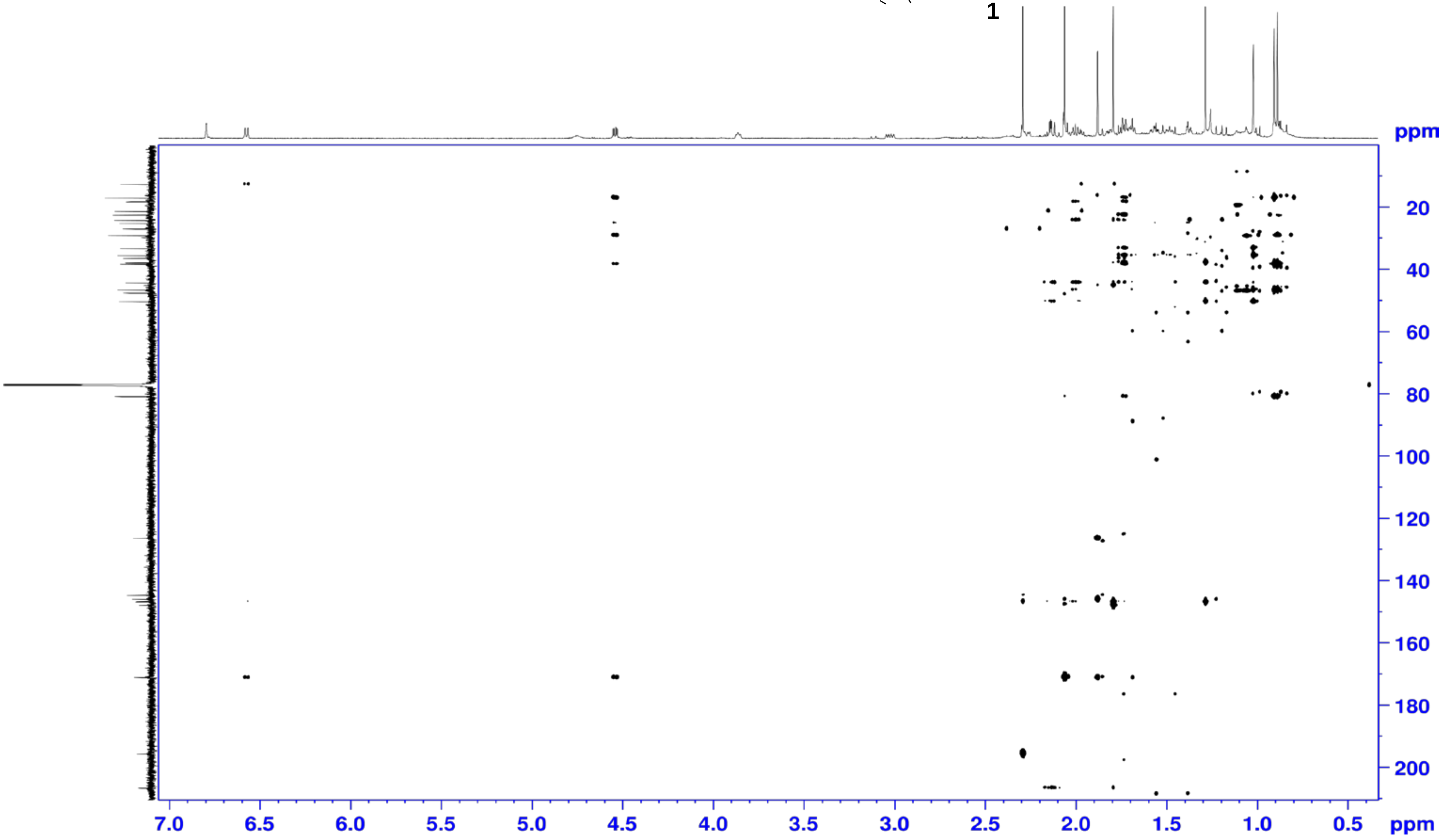

S8 COSY Spectrum of Stellettin Q (**1**) in $CDCl_3$ (700 MHz)

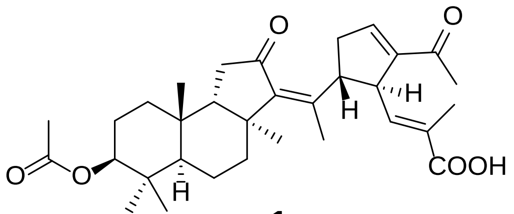

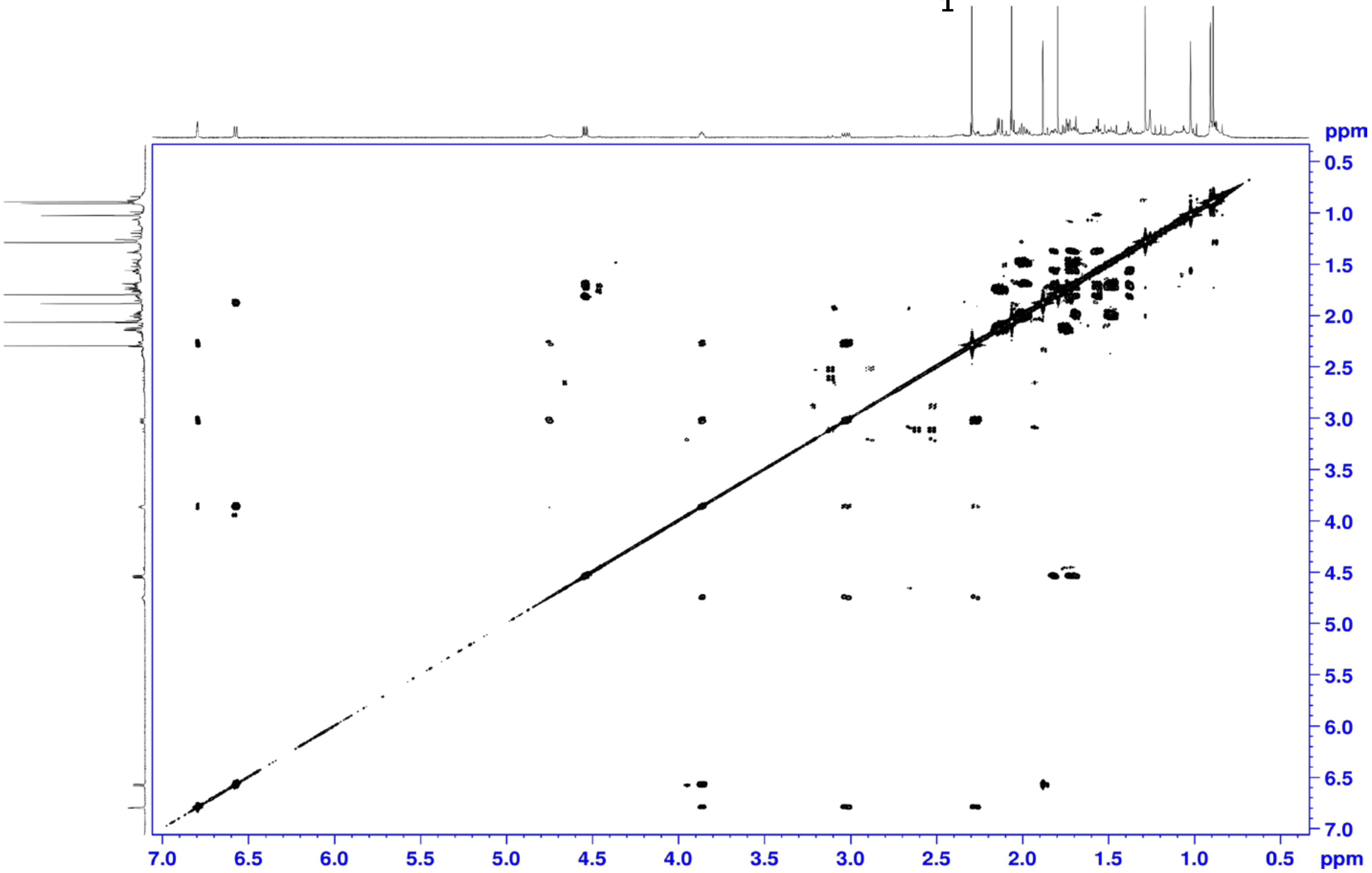

S9 ROESY Spectrum of Stellettin Q (**1**) in $CDCl_3$ (700 MHz)

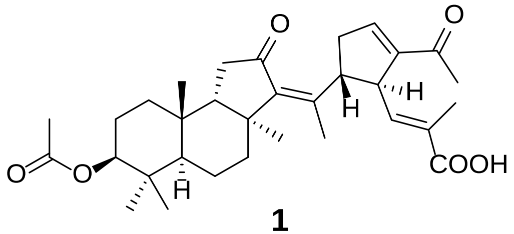

1

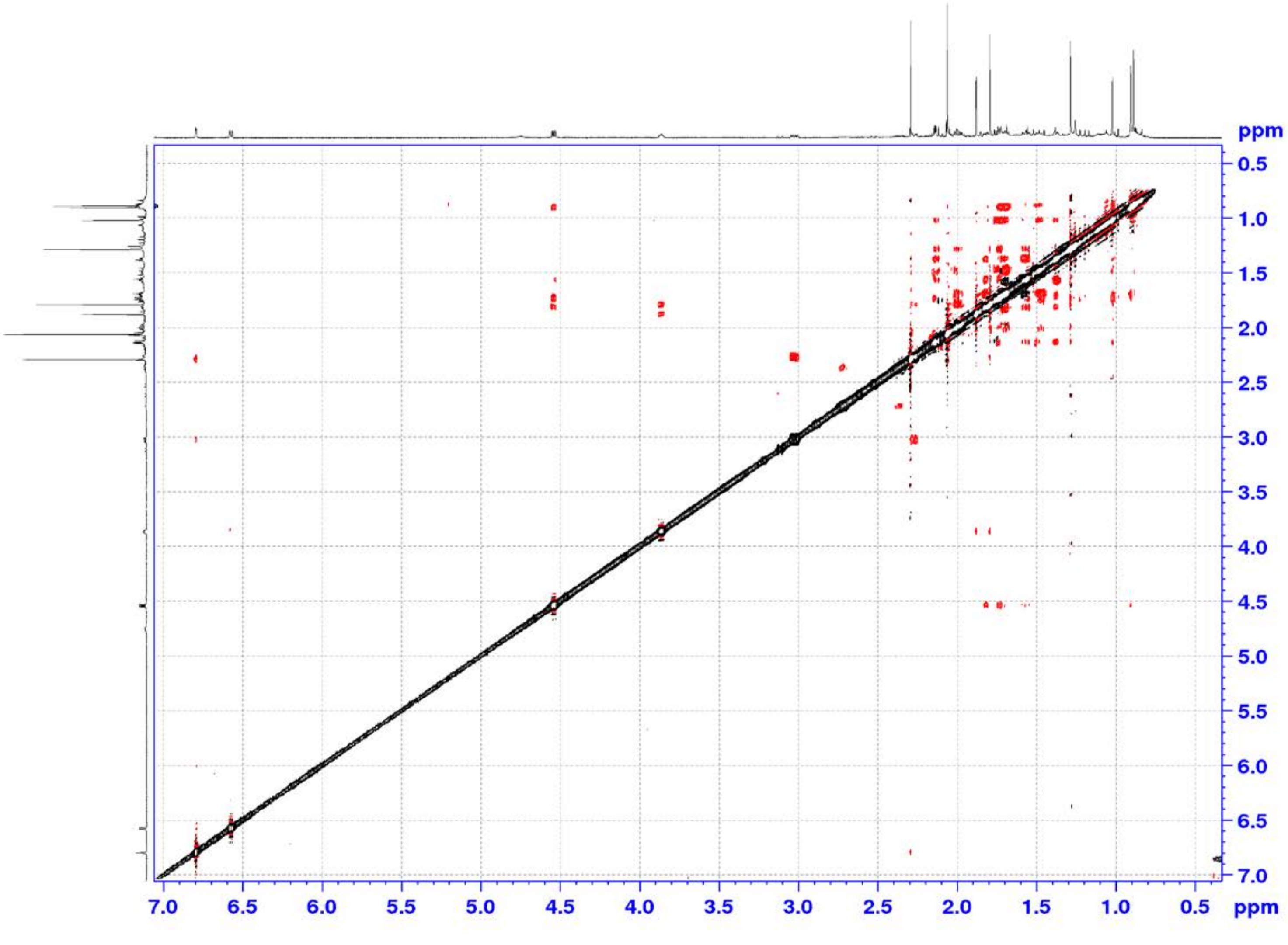

S10 ECD Spectrum of Stellettin Q (**1**) in EtOH

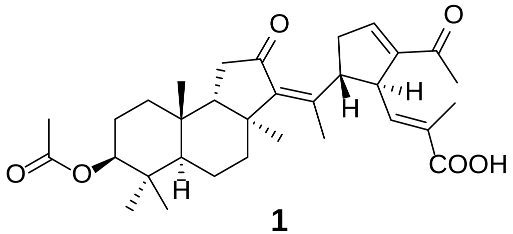

1

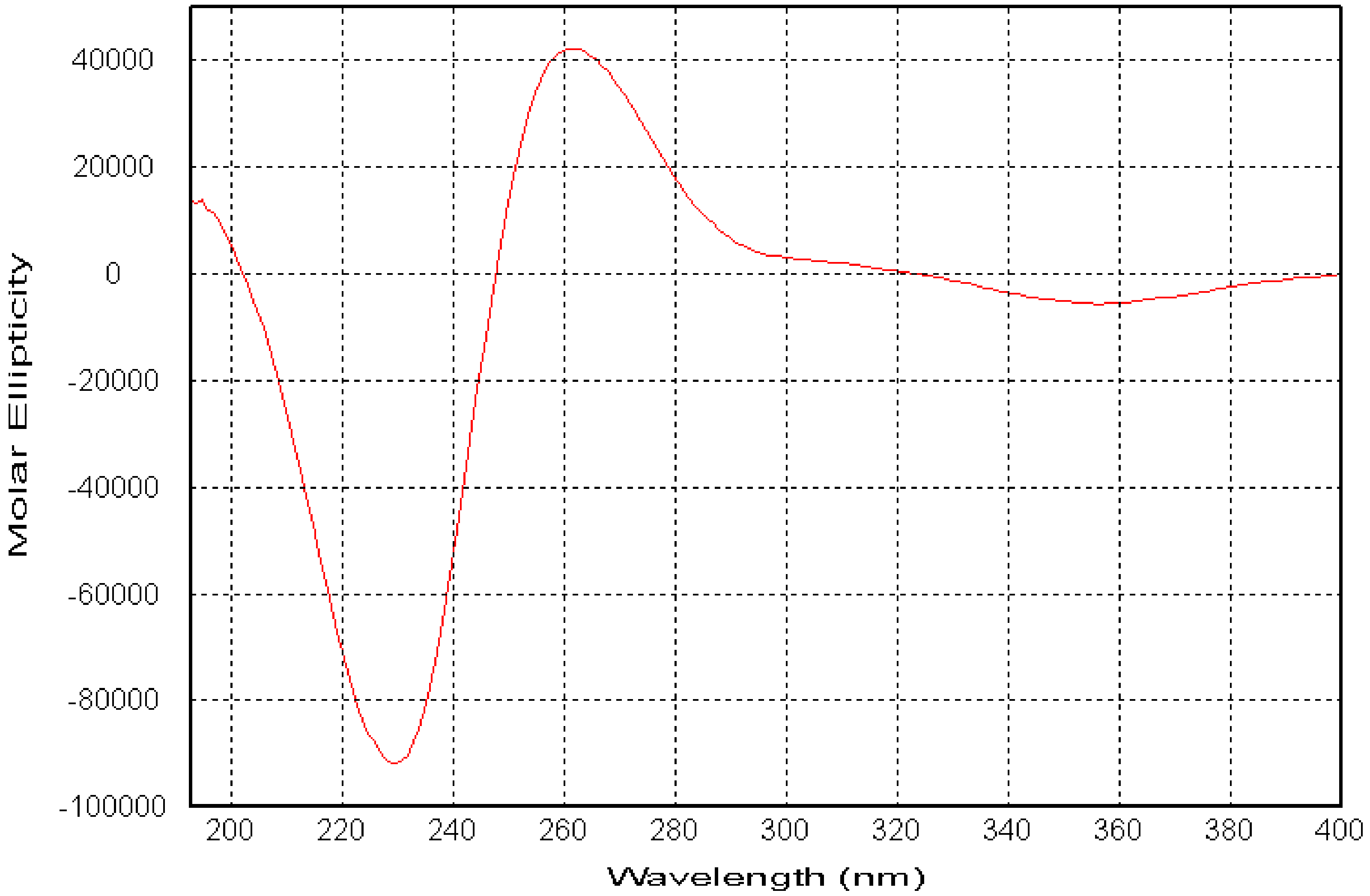

S11 HRESIMS and MS/MS Spectra (Negative Ion Mode) of Stellettin R (**2**)

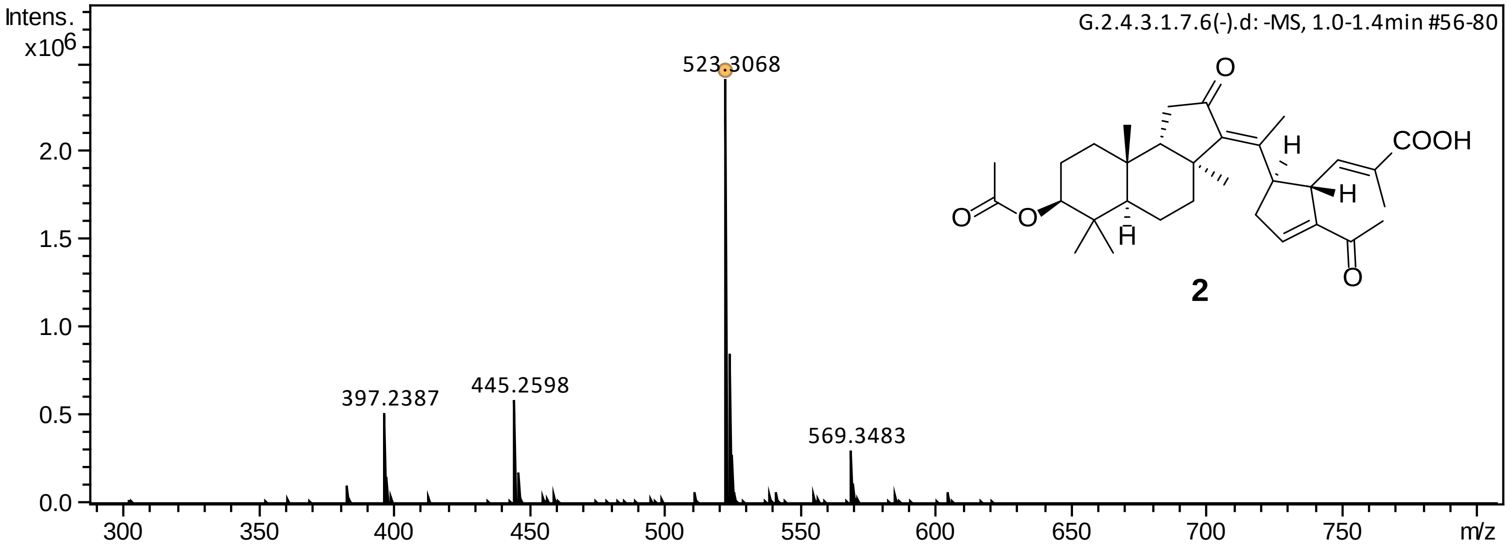

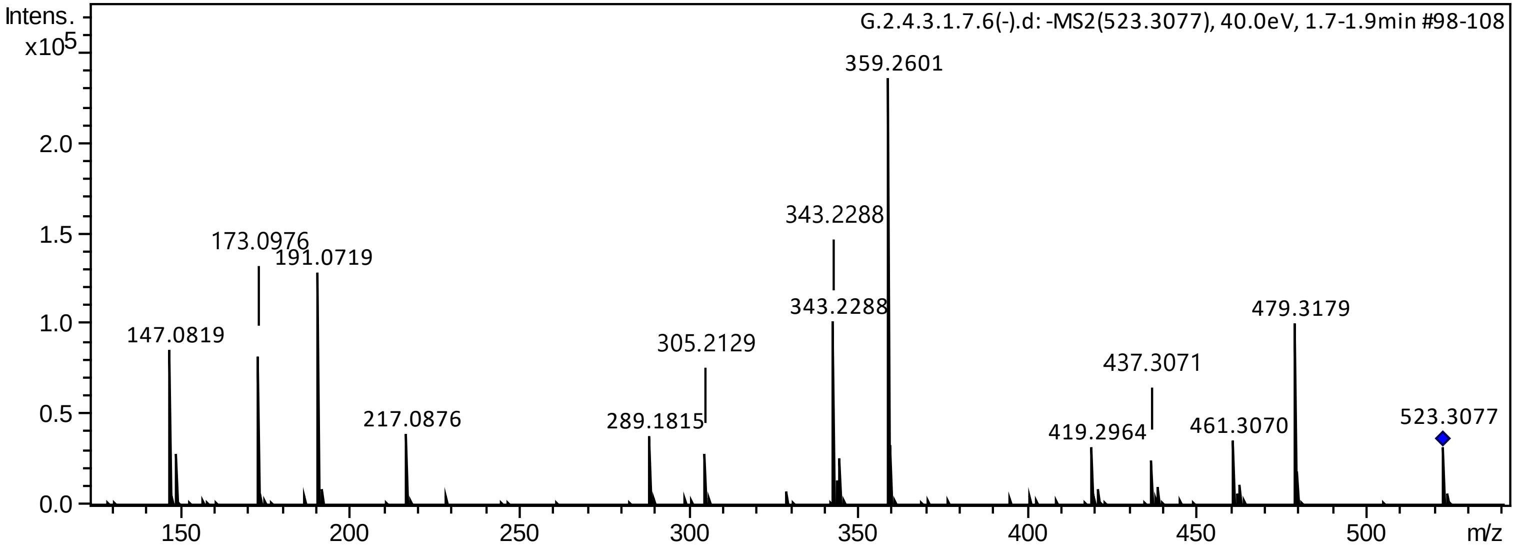

S12 ^{1}H NMR Spectrum of Stellettin R (**2**) in $CDCl_3$ (500 MHz)

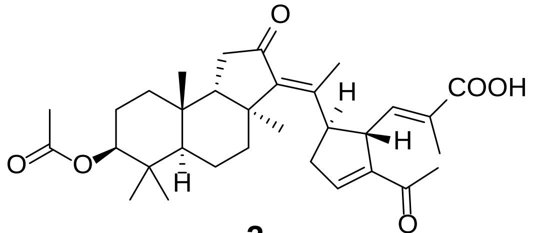

2

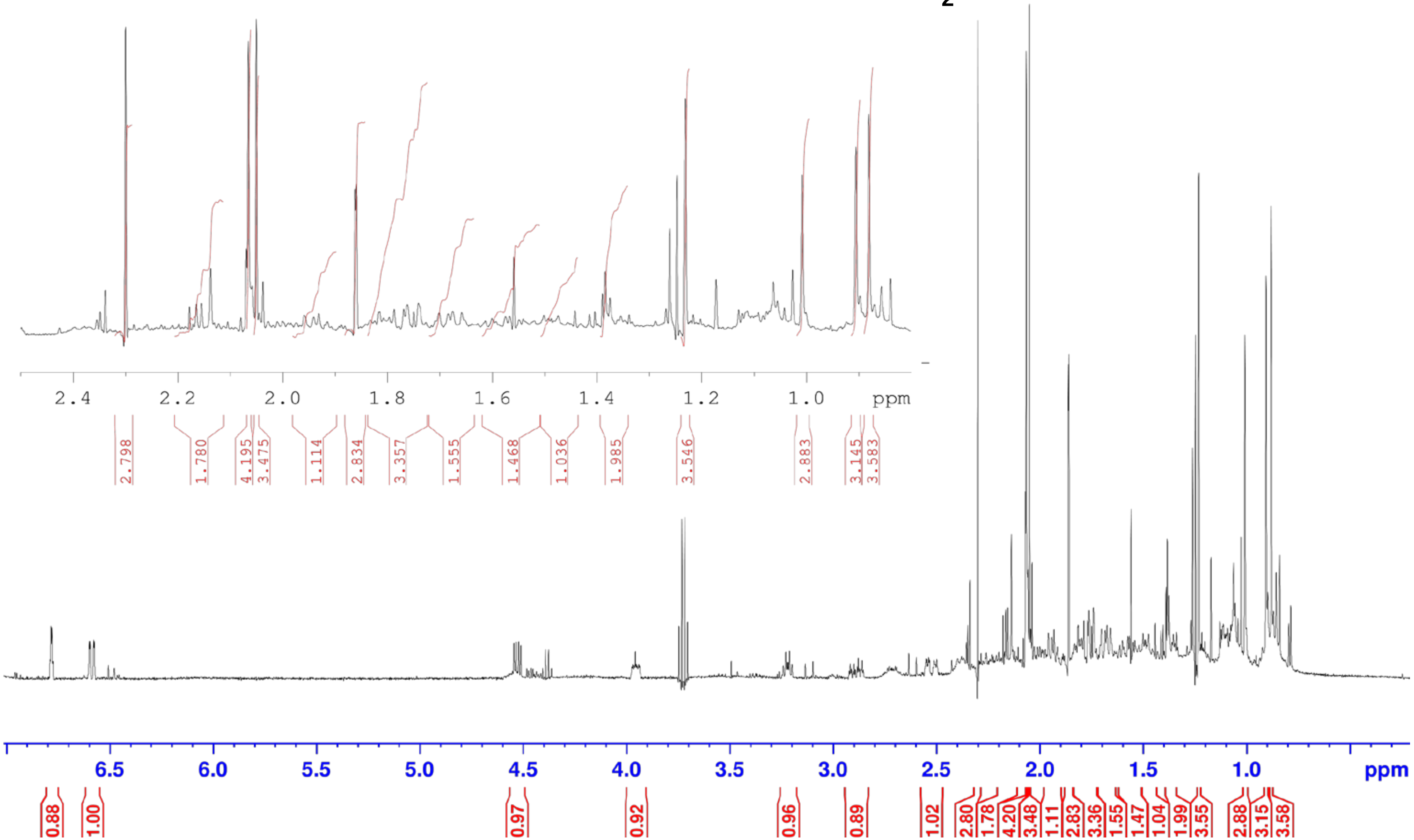

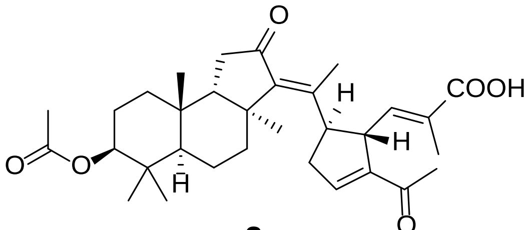

2

S13 ^{13}C NMR Spectrum of Stelletin R (**2**) in $CDCl_3$ (126 MHz)

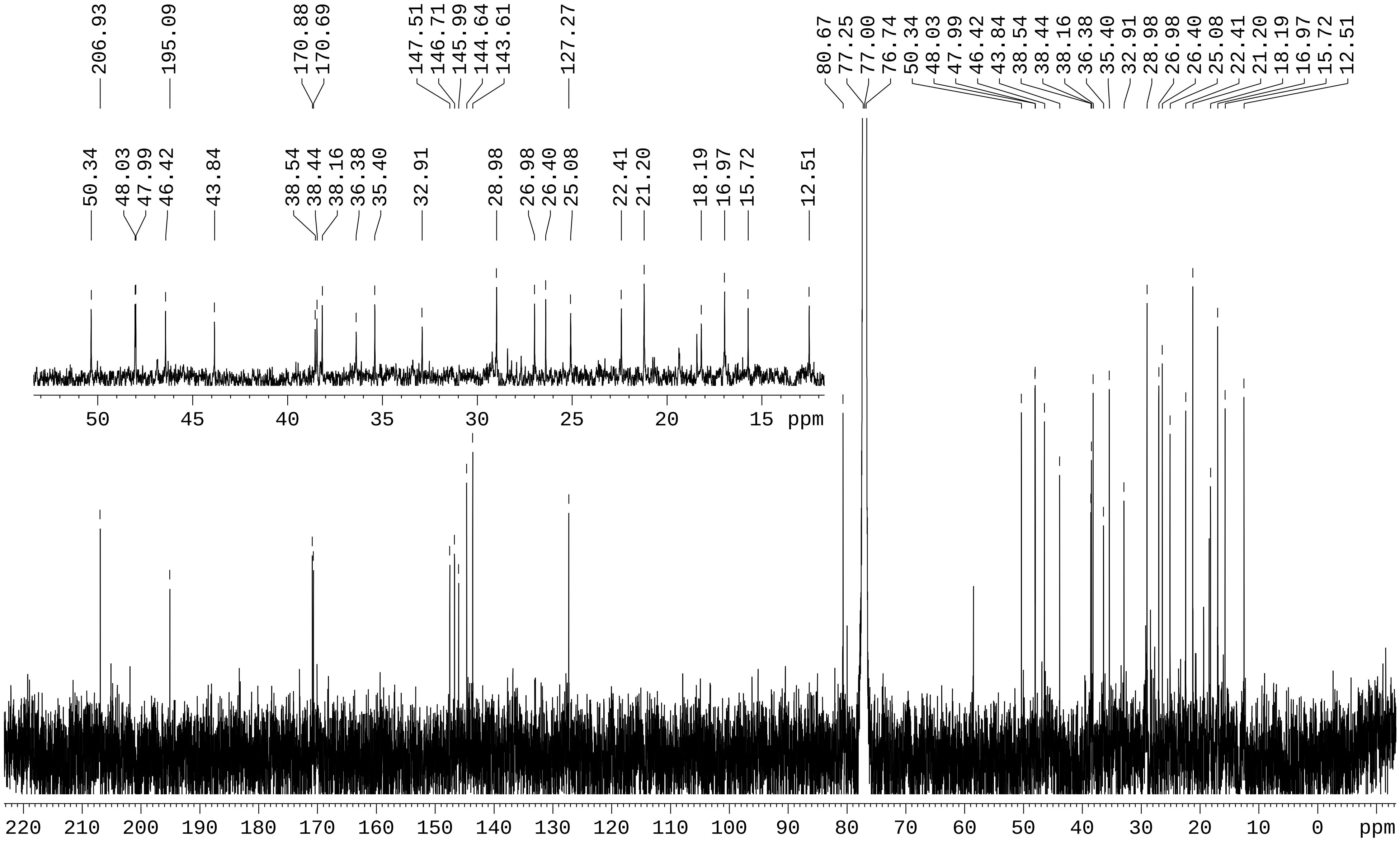

S14 HSQC Spectrum of Stellettin R (**2**) in $CDCl_3$ (500 MHz)

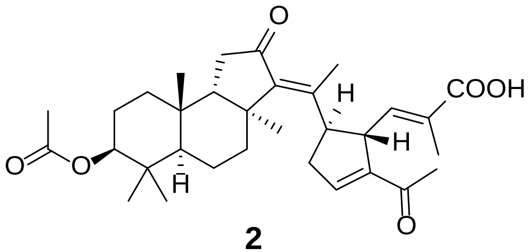

2

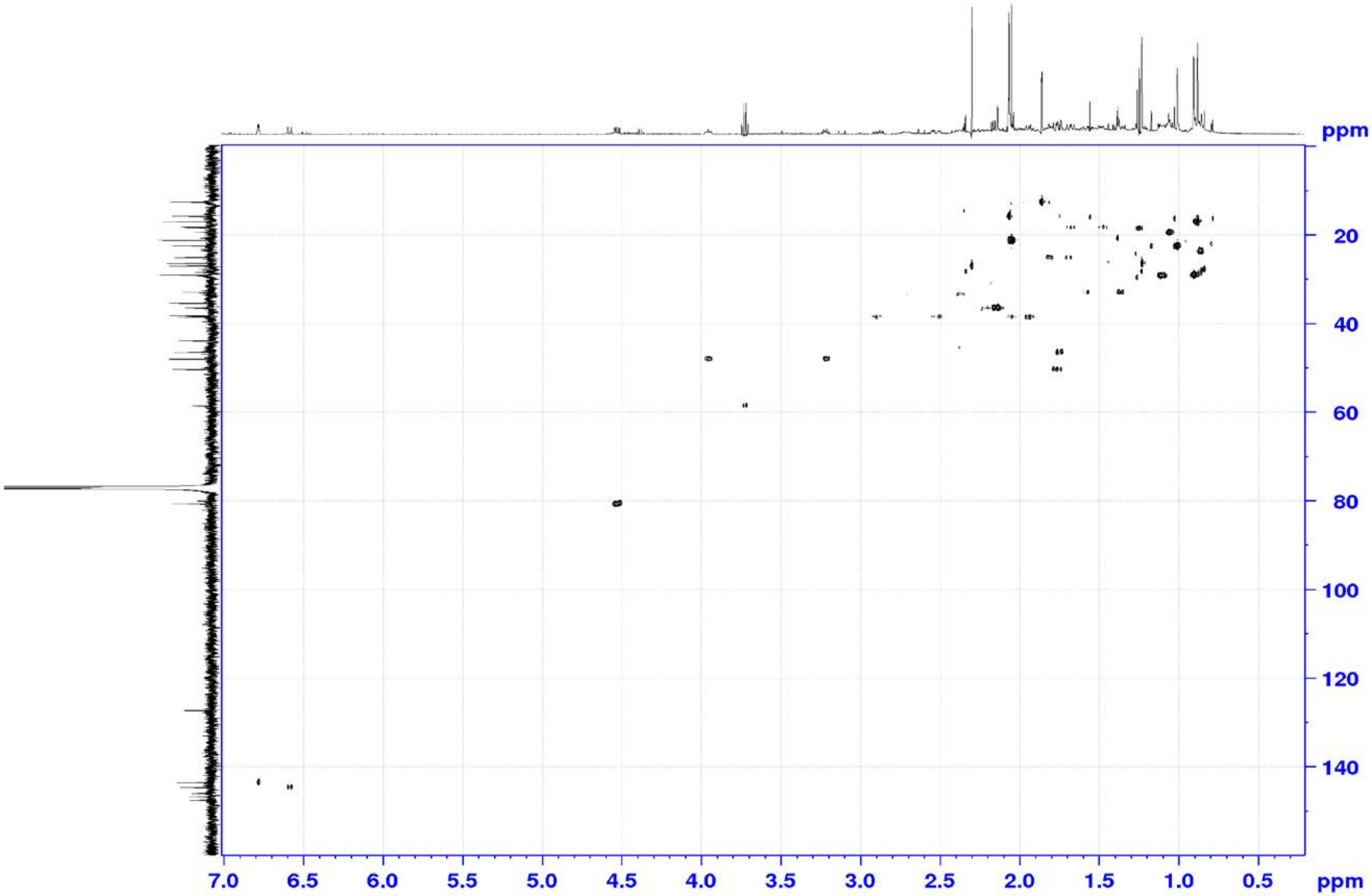

S15 HMBC Spectrum of Stellettin R (**2**) in $CDCl_3$ (500 MHz)

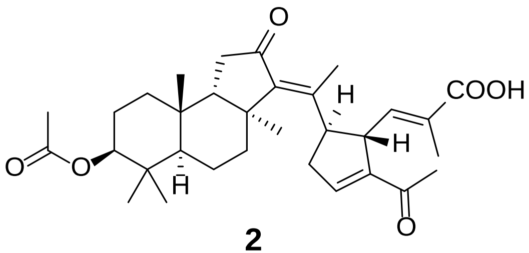

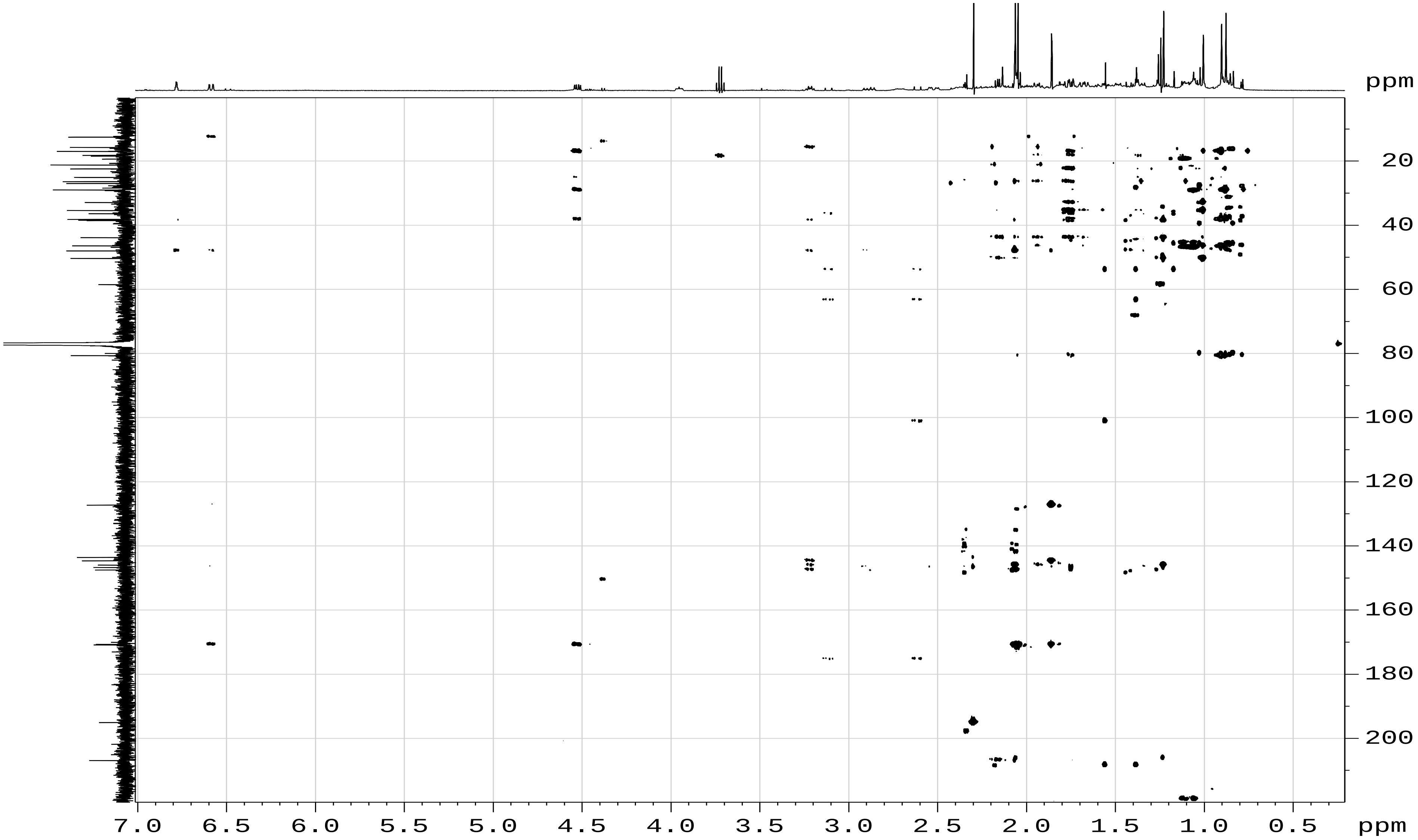

S16 COSY Spectrum of Stellettin R (**2**) in $CDCl_3$ (500 MHz)

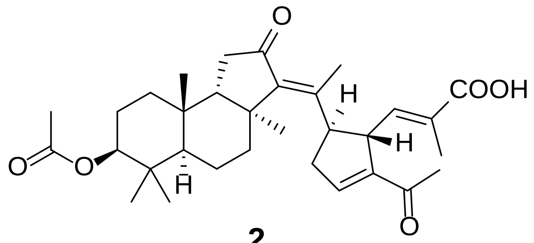

2

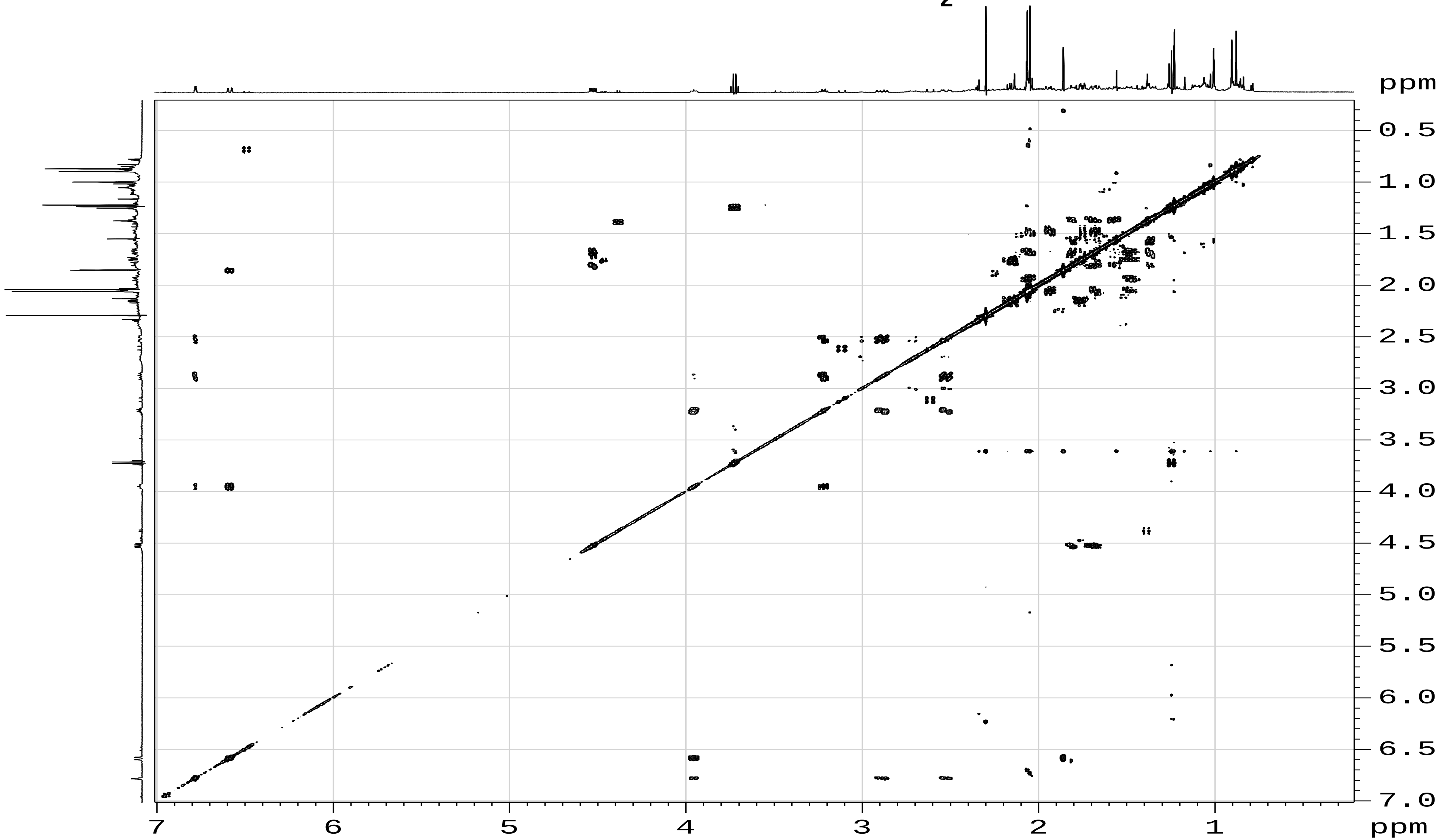

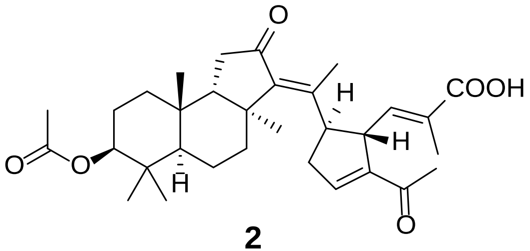

2

S17 ROESY Spectrum of Stellettin R (**2**) in $CDCl_3$ (500 MHz)

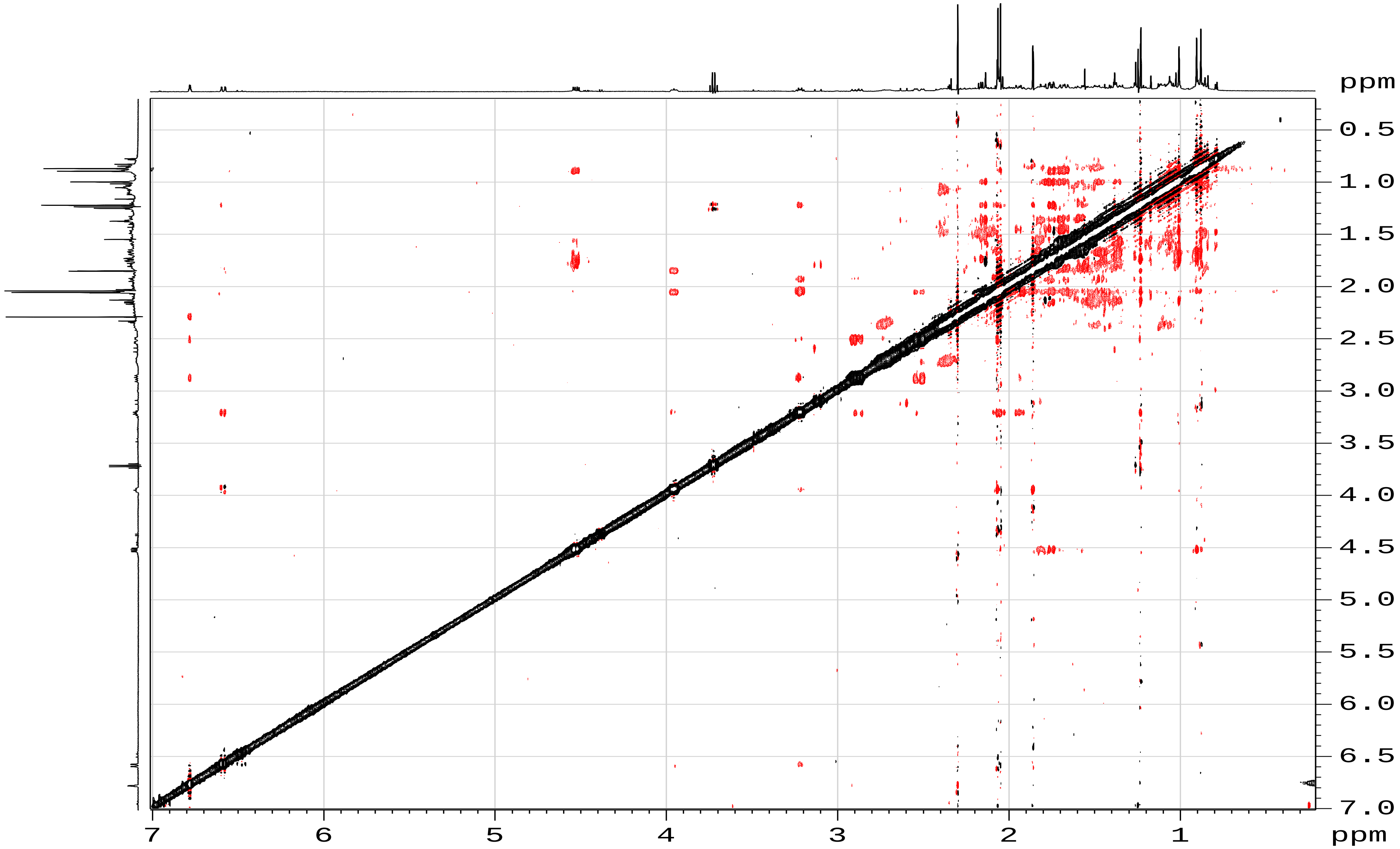

S18 ECD Spectrum of Stelletin R (**2**) in EtOH

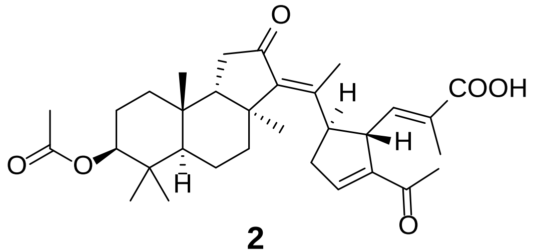

2

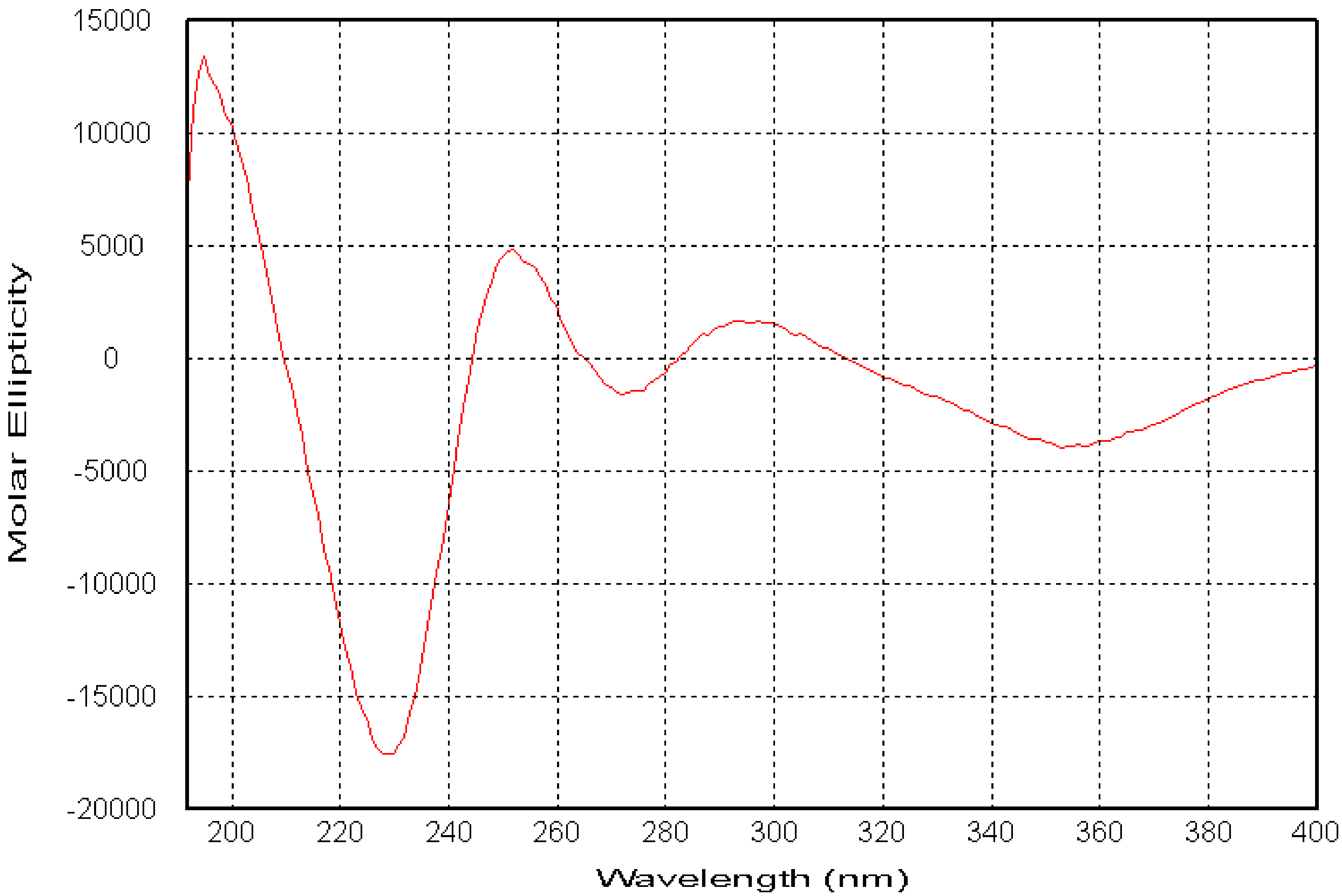

S19 HRESIMS and MS/MS Spectra (Negative Ion Mode) of Stellettin S (**3**)

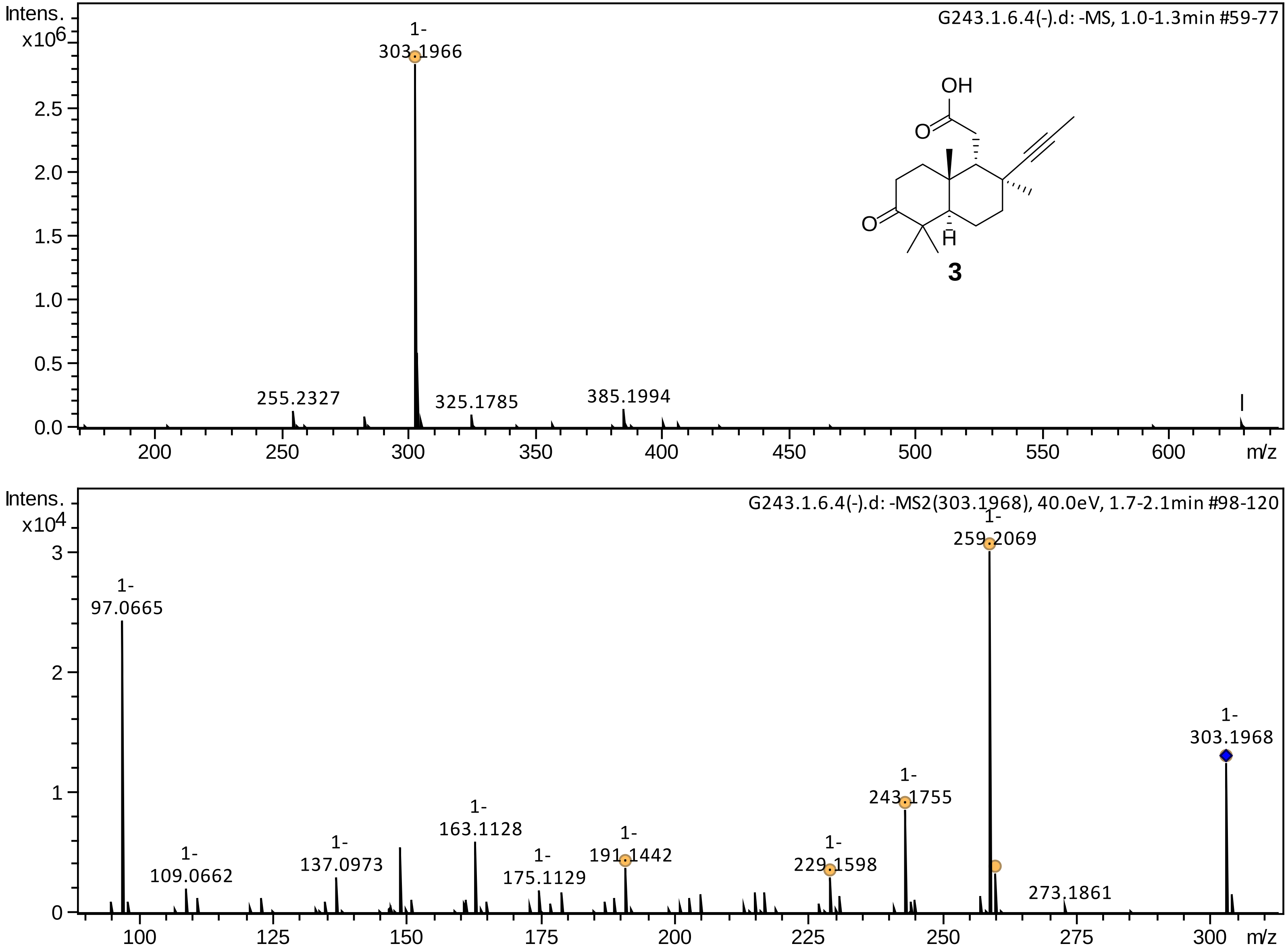

S20 ^{1}H NMR Spectrum of Stellettin S (**3**) in $CDCl_3$ (700 MHz)

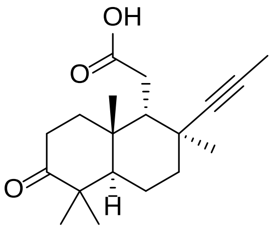

3

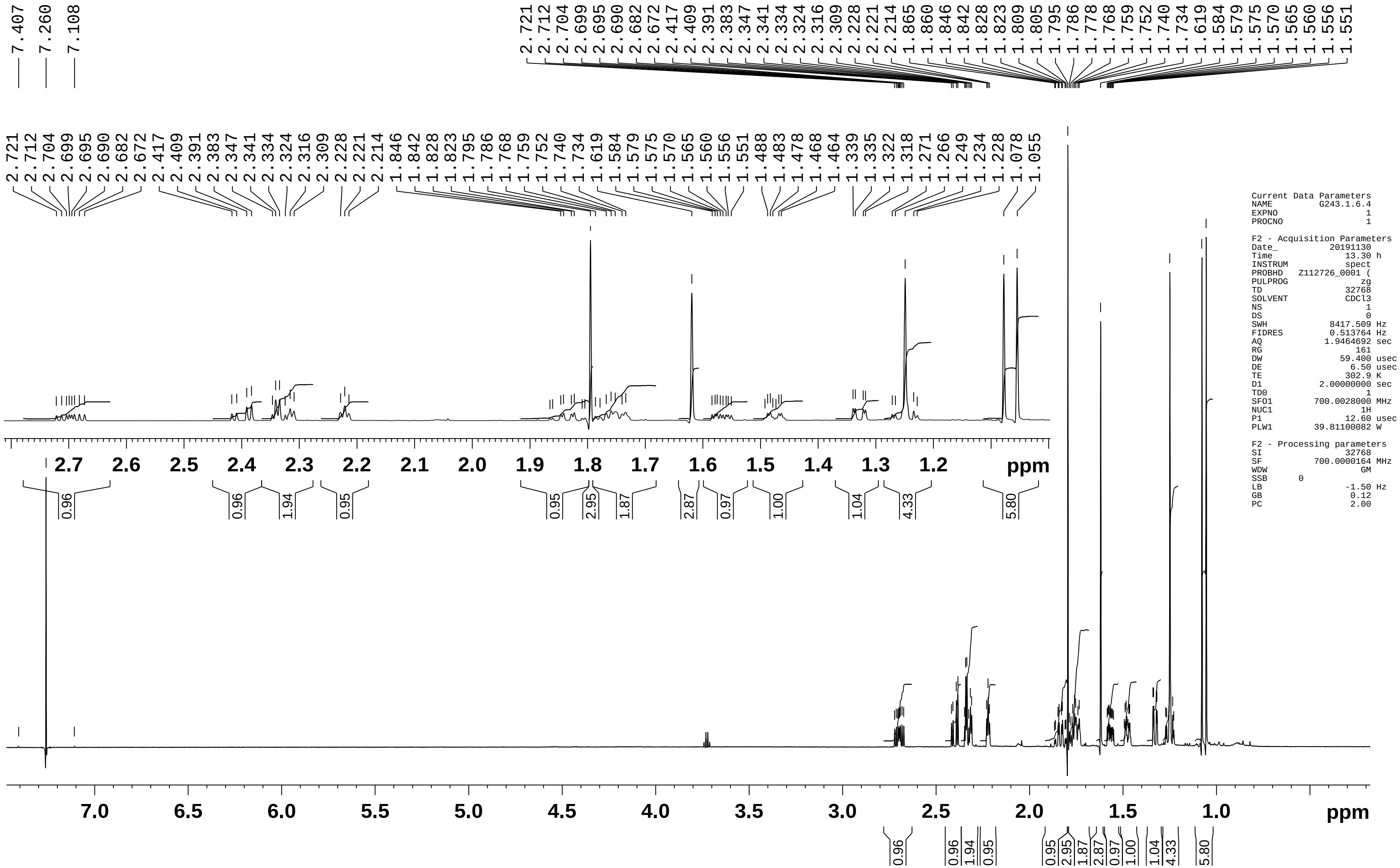

S21 ^{13}C NMR Spectrum of Stellettin S (**3**) in $CDCl_3$ (176 MHz)

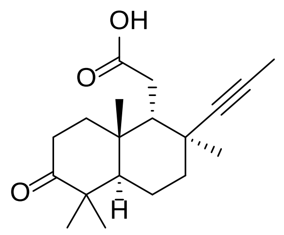

3

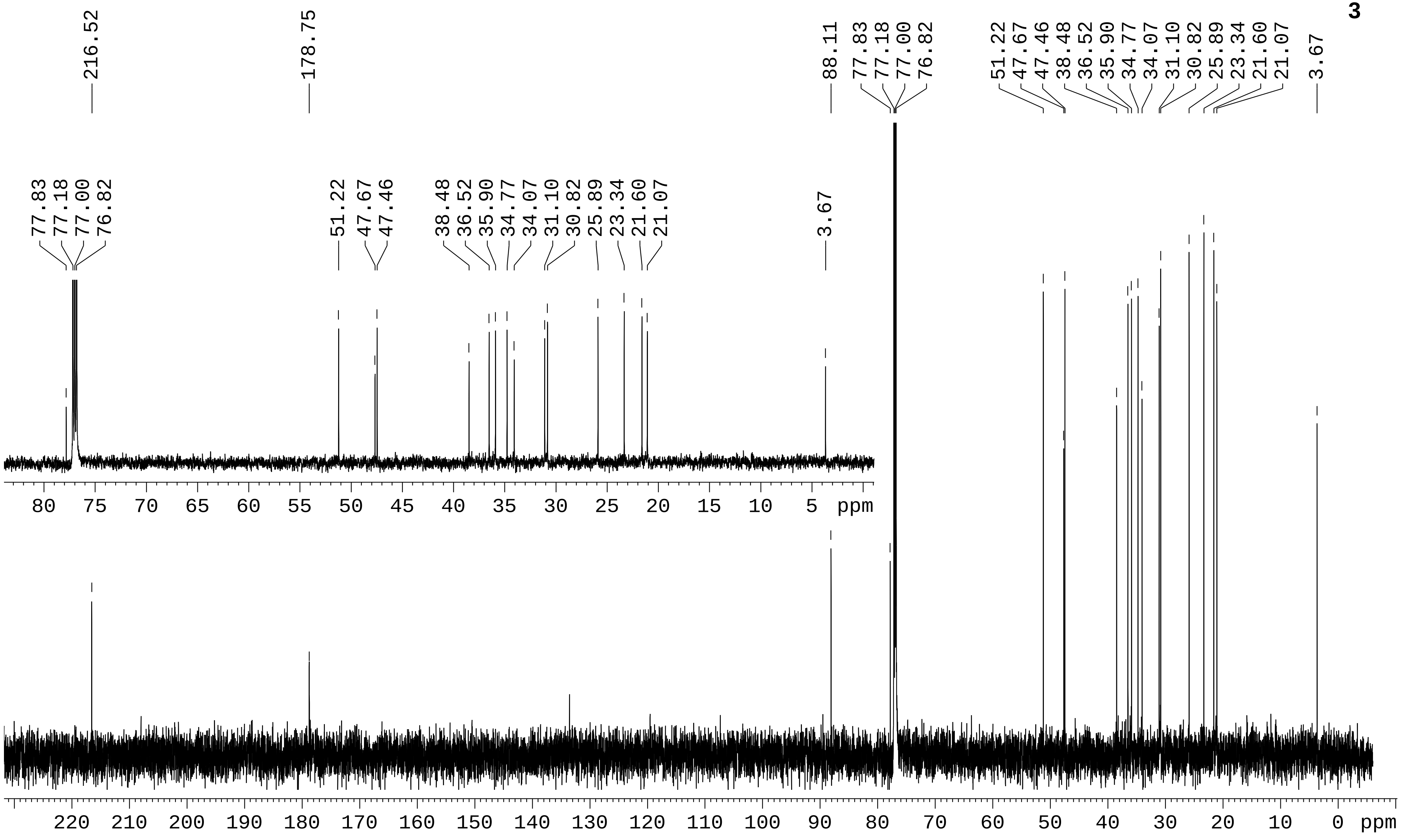

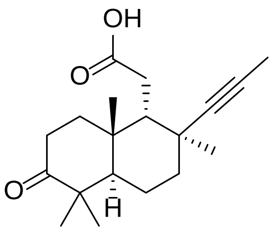

3

S22 DEPT Spectrum of Stelletin S (**3**) in $CDCl_3$ (176 MHz)

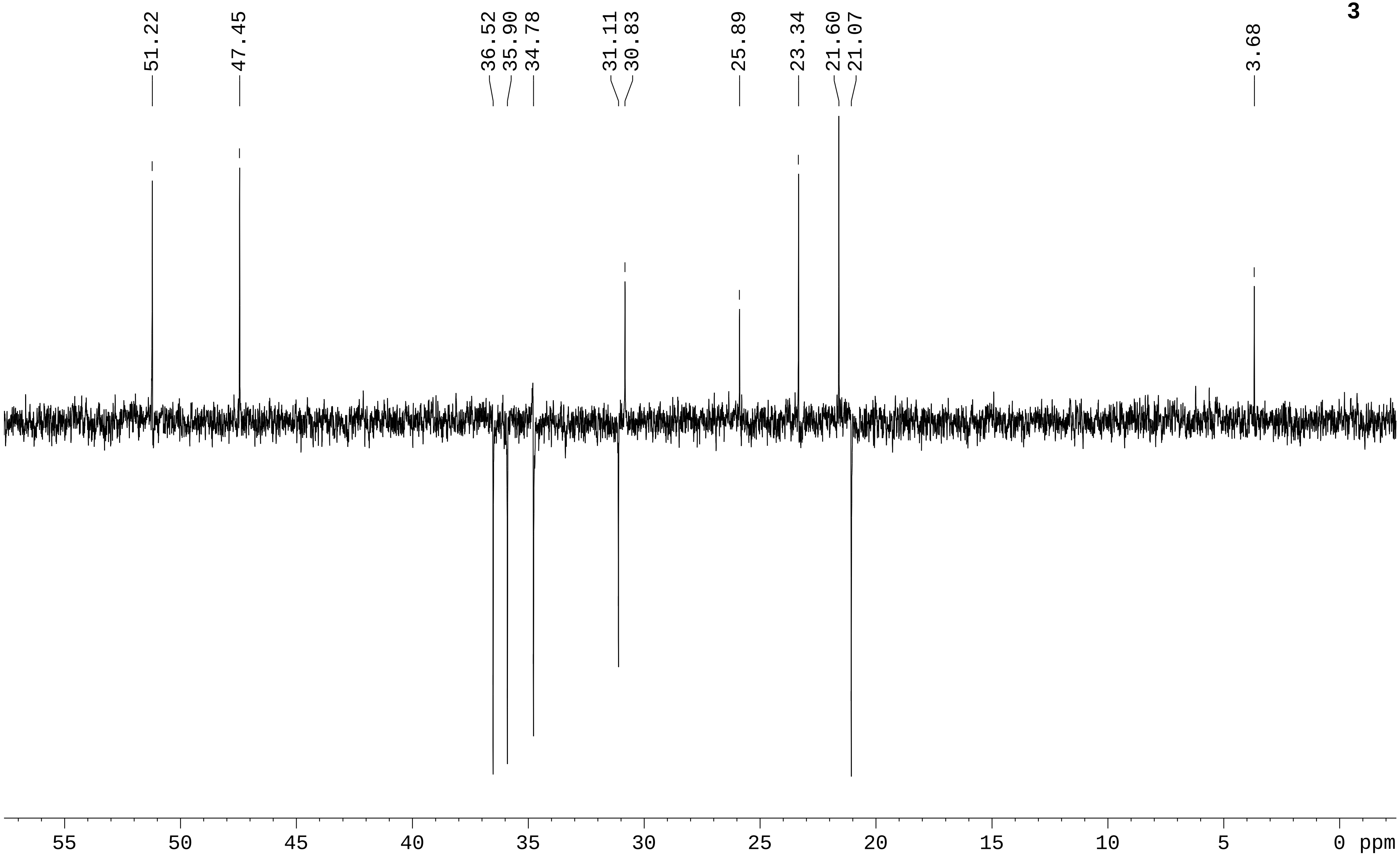

S23 HSQC Spectrum of Stellettin S (**3**) in $CDCl_3$ (700 MHz)

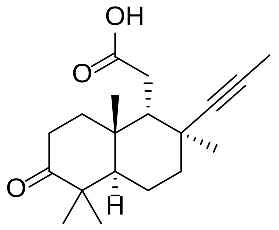

3

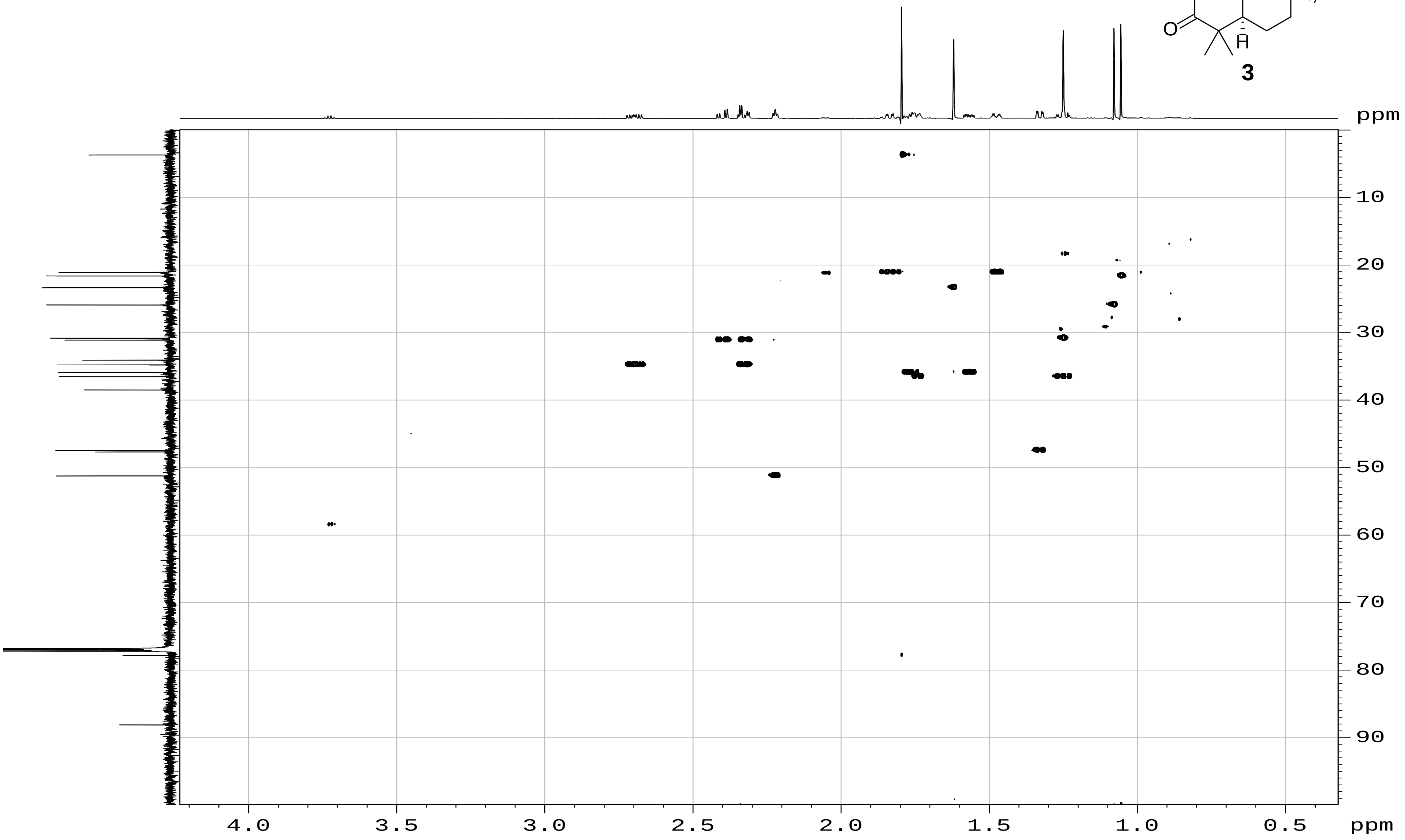

S24 HMBC Spectrum of Stelletin S (**3**) in $CDCl_3$ (700 MHz)

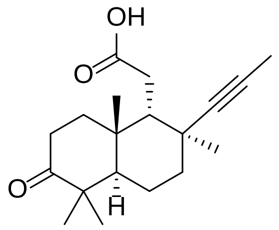

3

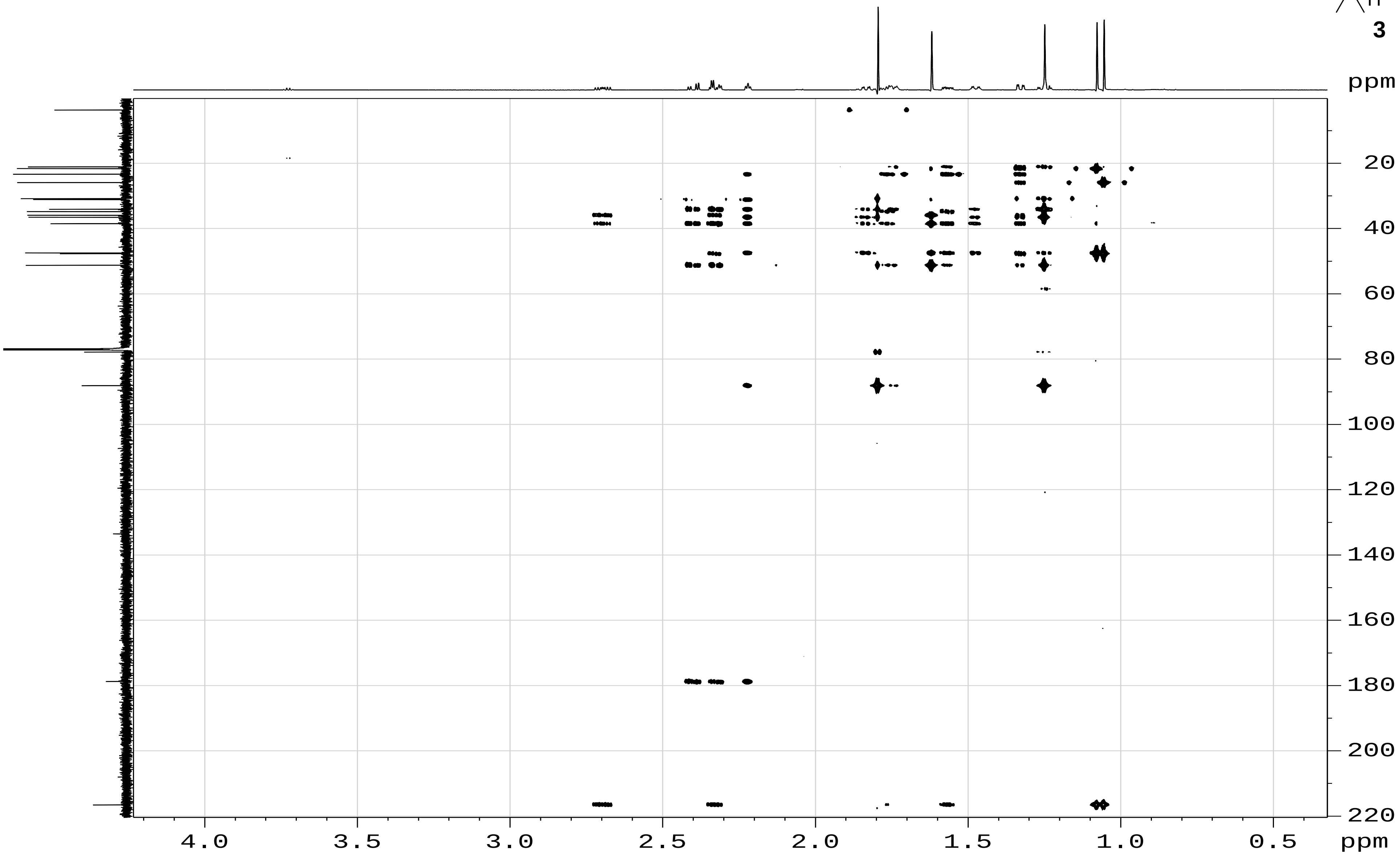

S25 COSY Spectrum of Stellettin S (**3**) in $CDCl_3$ (700 MHz)

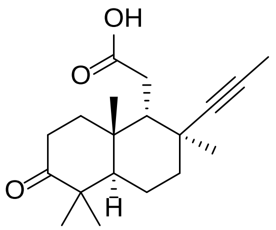

3

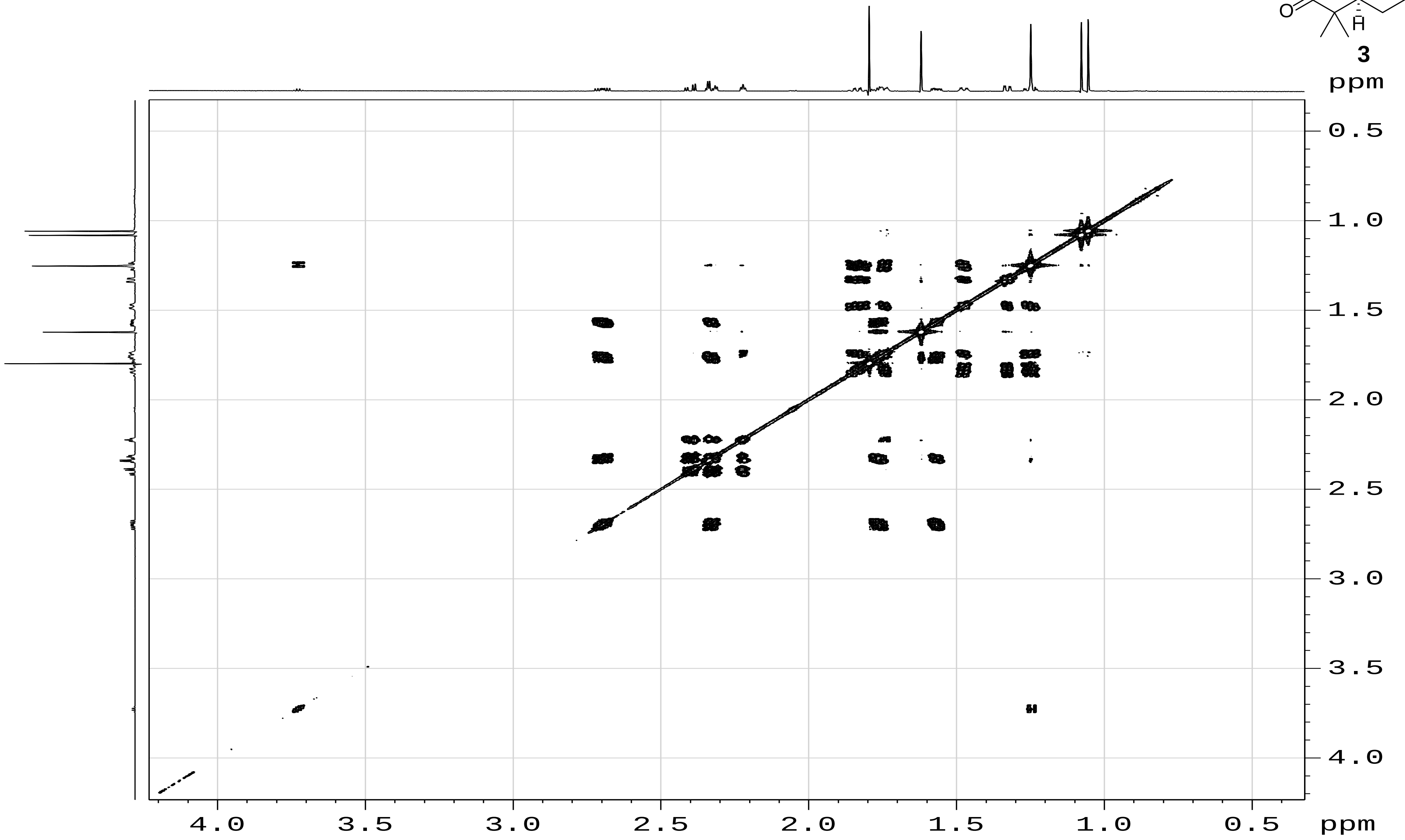

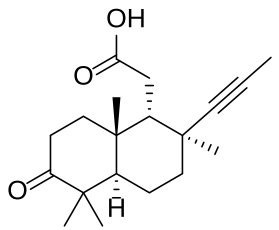

3

S26 ROESY Spectrum of Stellettin S (**3**) in $CDCl_3$ (700 MHz)

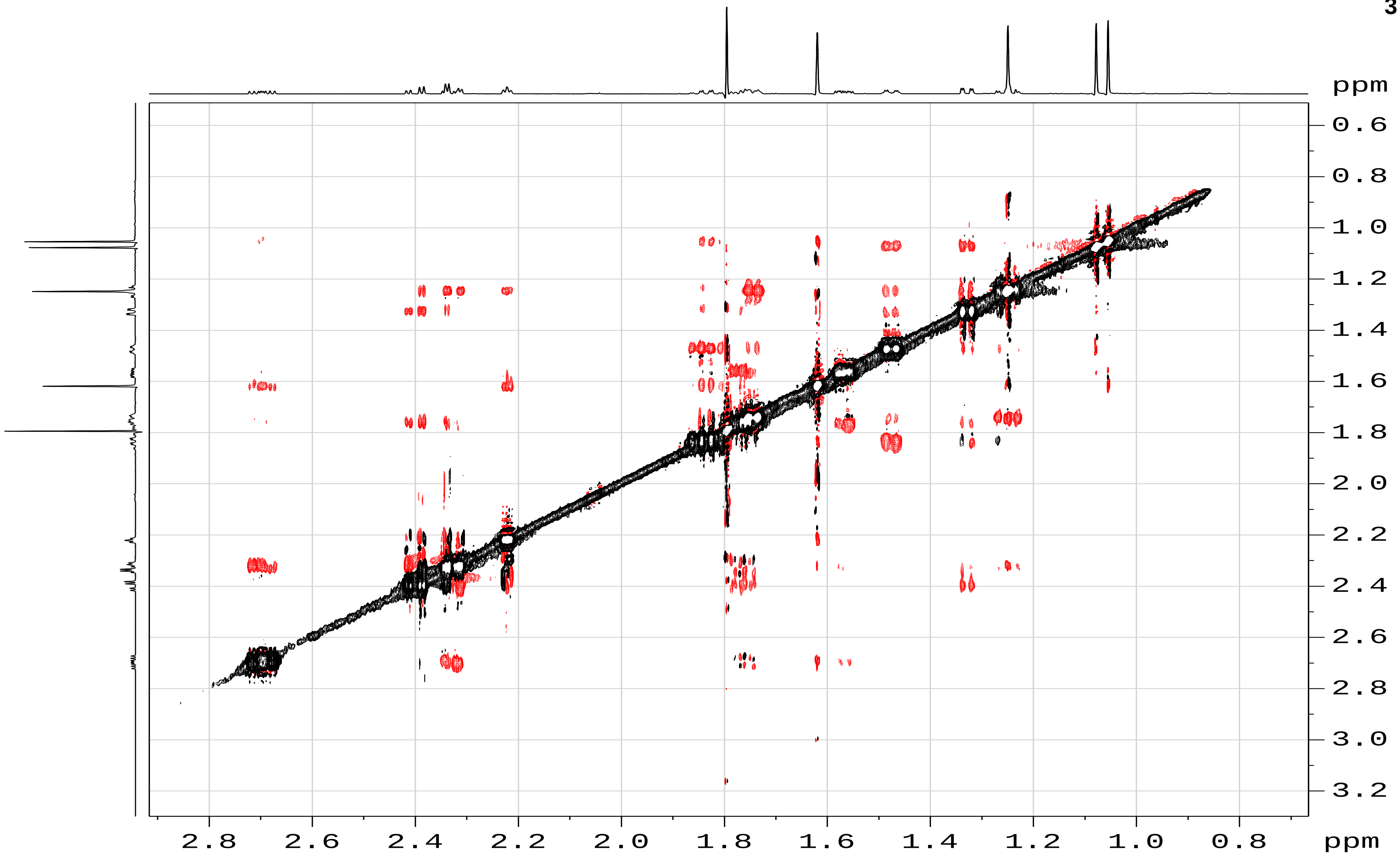

S27 ECD Spectrum of Stellettin S (**3**) in EtOH

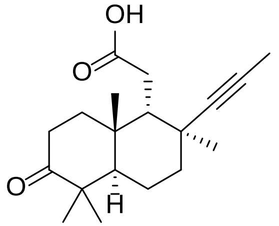

3

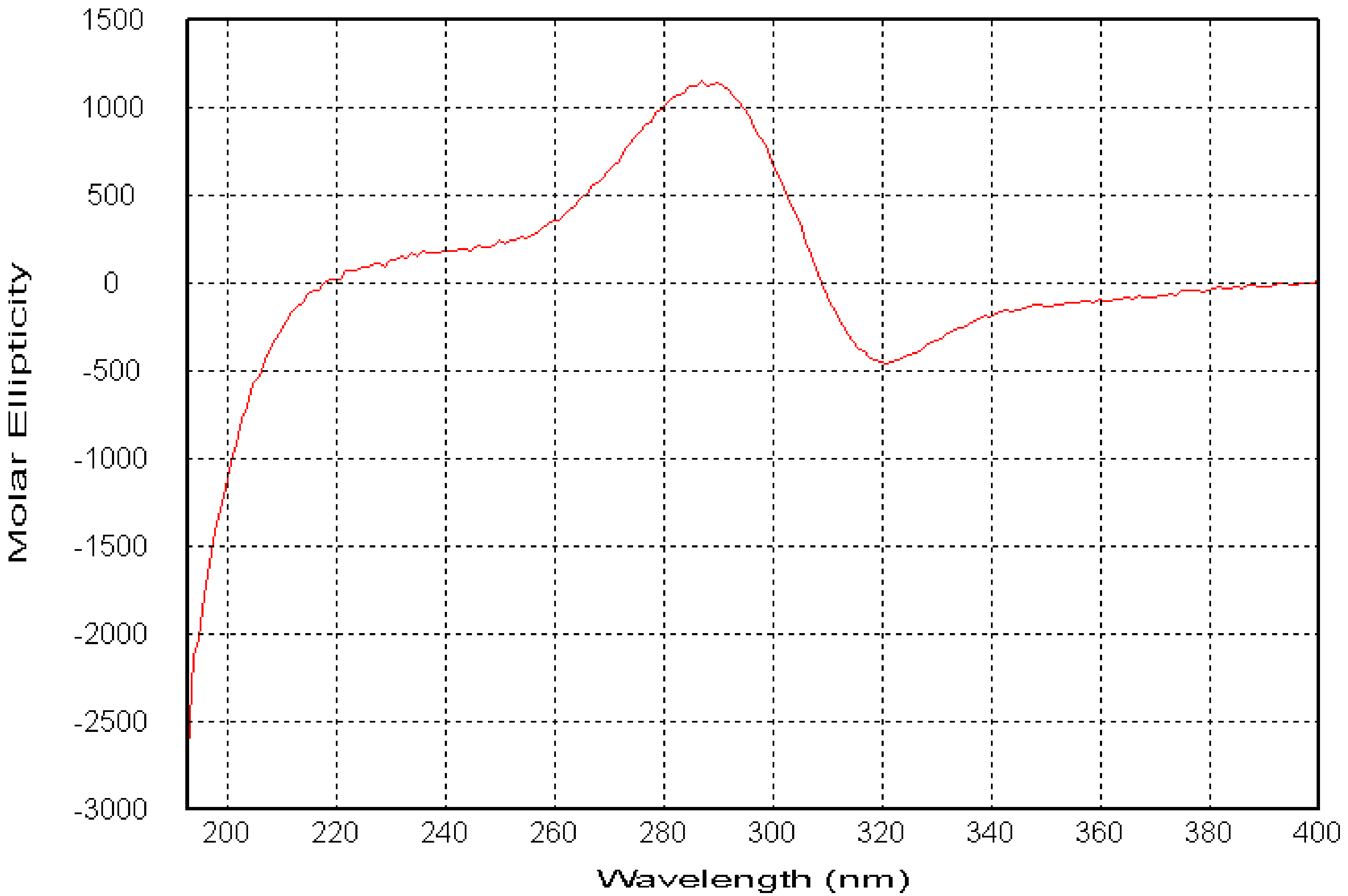

S28 HRESIMS and MS/MS Spectra (Negative Ion Mode) of Stellettin T (**4**)

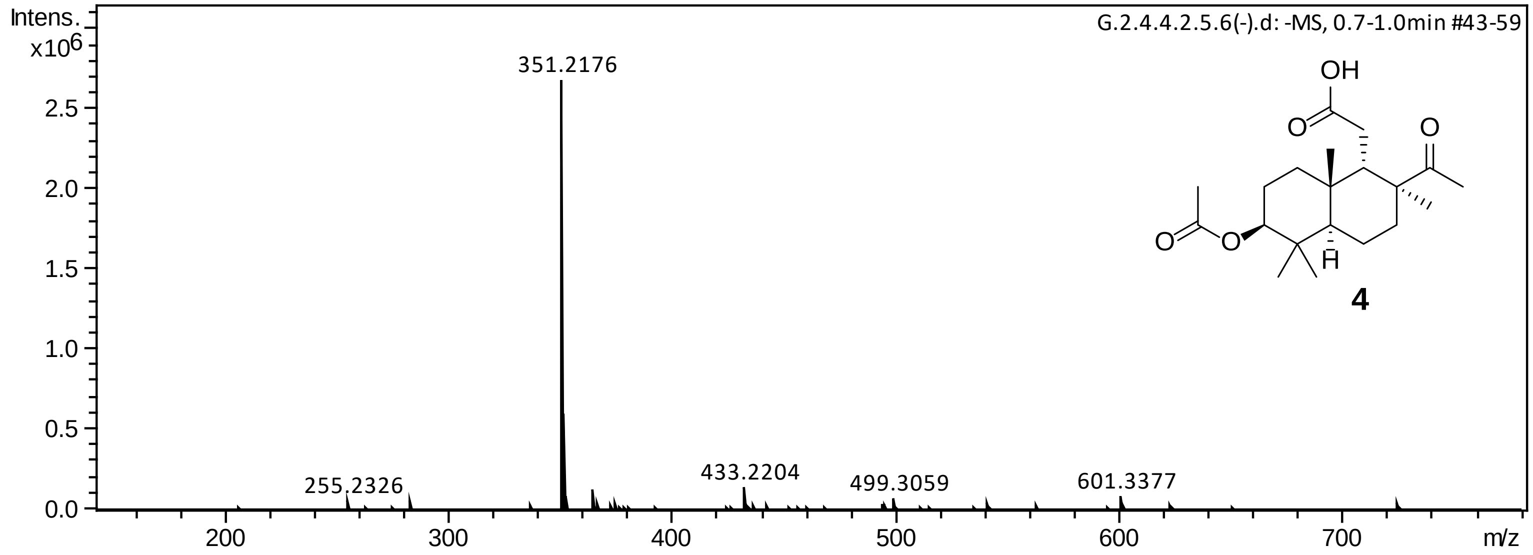

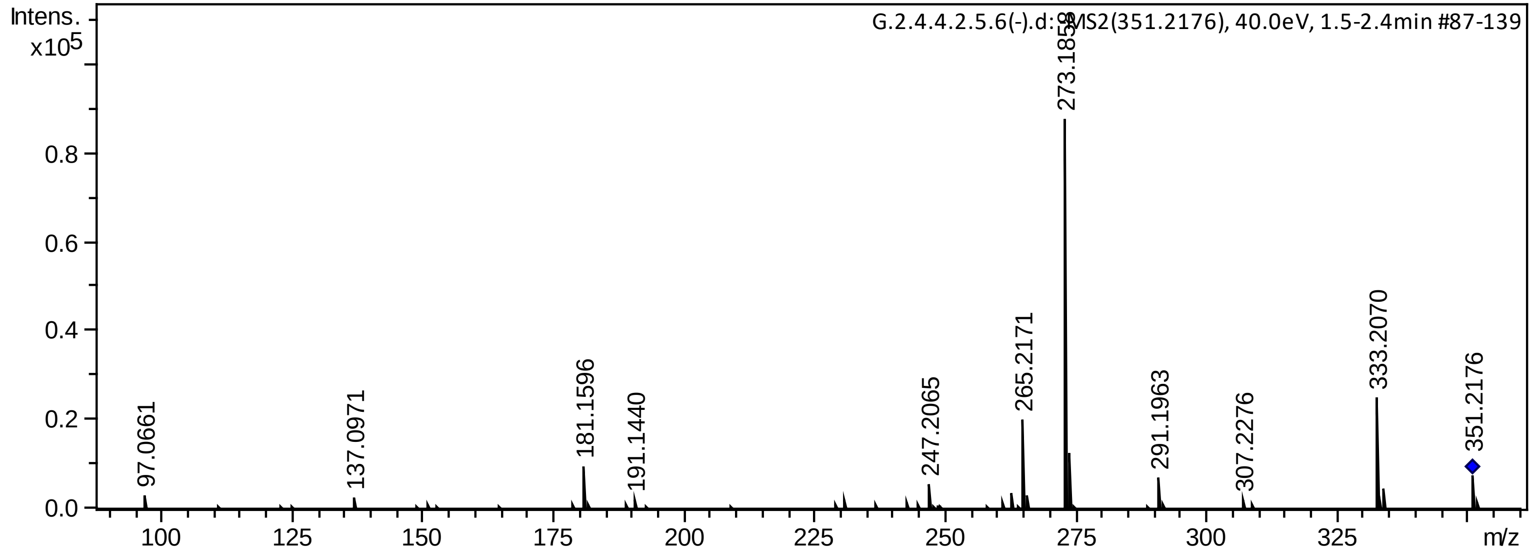

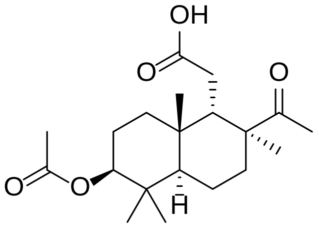

4

S29 ^{1}H NMR Spectrum of Stellettin T (**4**) in $CDCl_3$ (700 MHz)

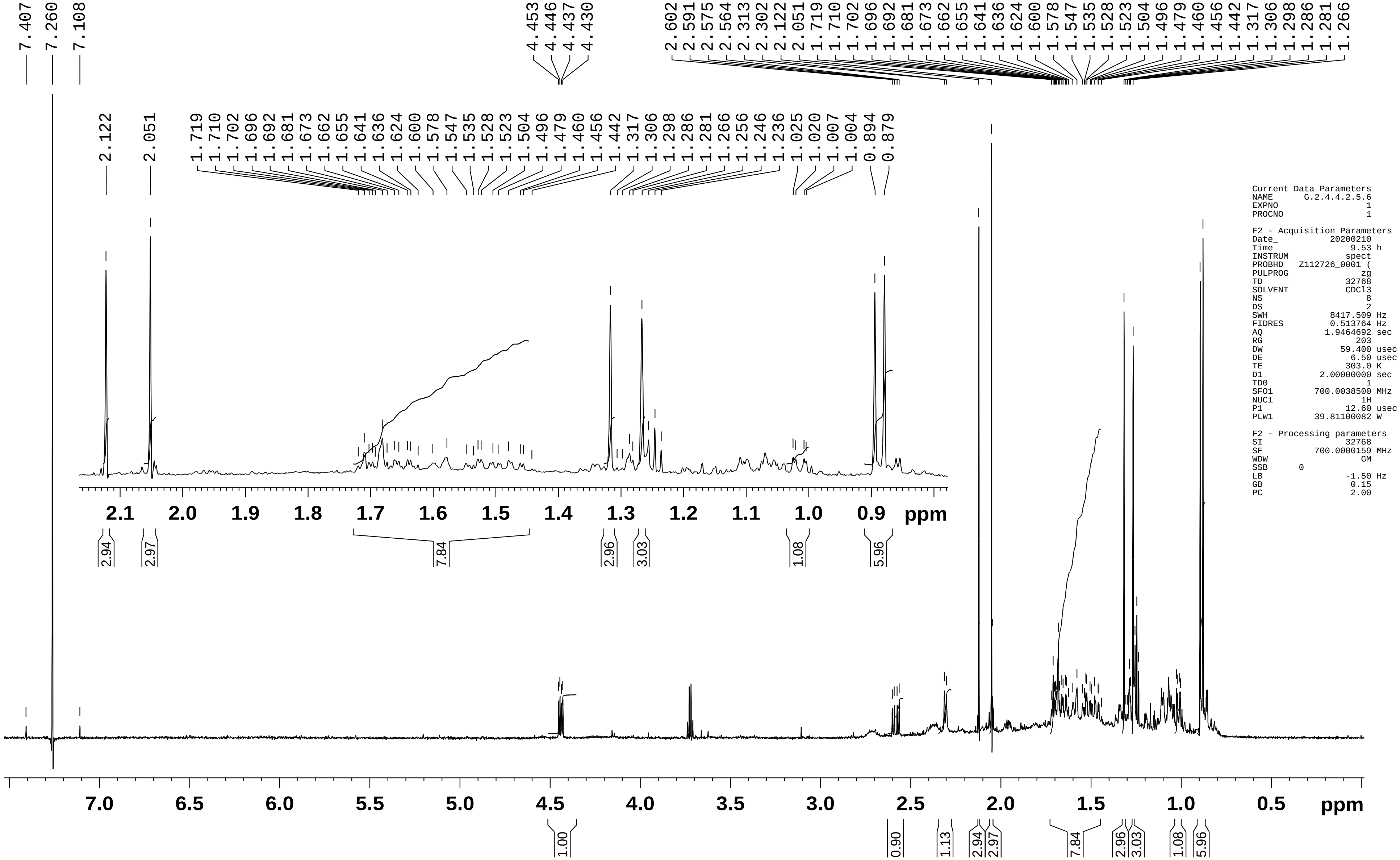

S30 ^{13}C NMR Spectrum of Stellettin T (**4**) in $CDCl_3$ (176 MHz)

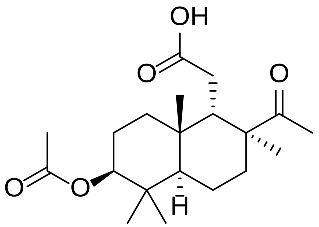

4

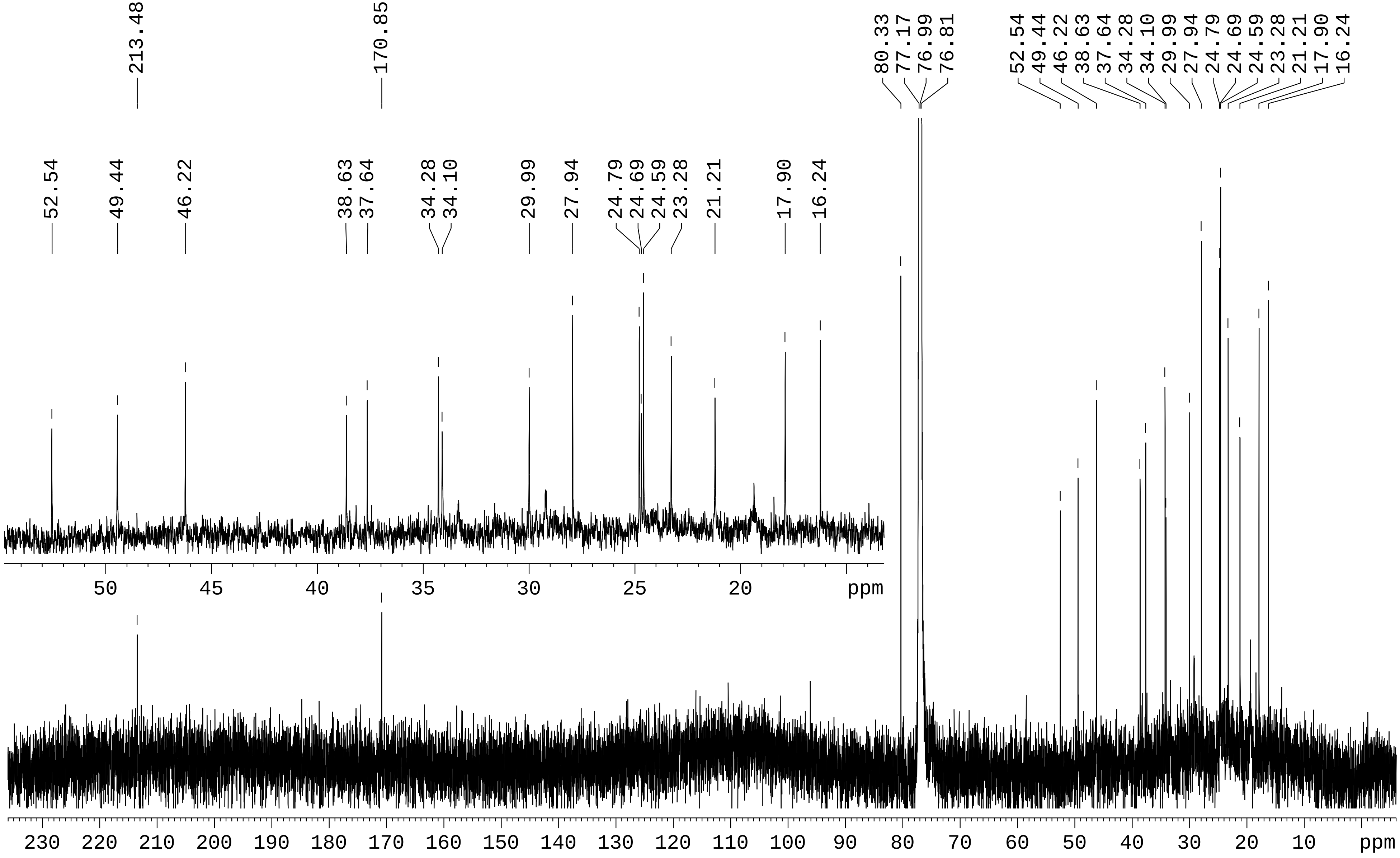

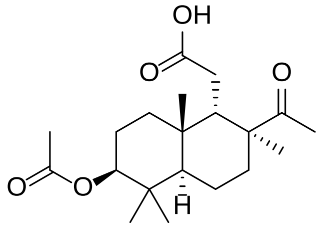

4

S31 HSQC Spectrum of Stellettin T (**4**) in $CDCl_3$ (700 MHz)

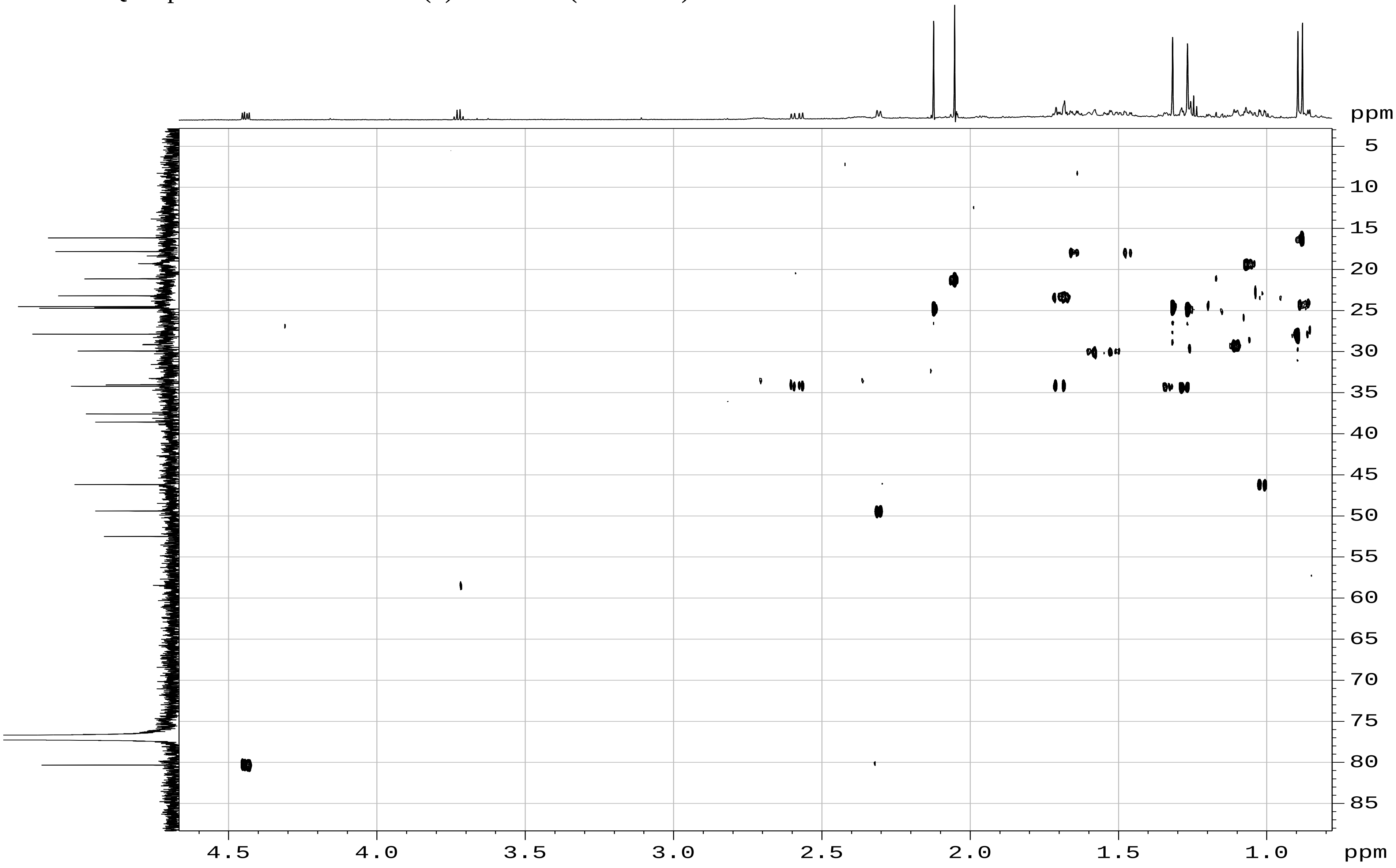

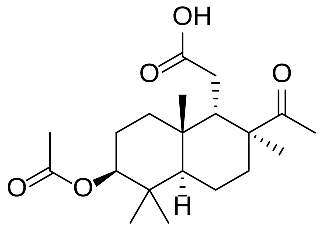

4

S32 HMBC Spectrum of Stellettin T (**4**) in $CDCl_3$ (700 MHz)

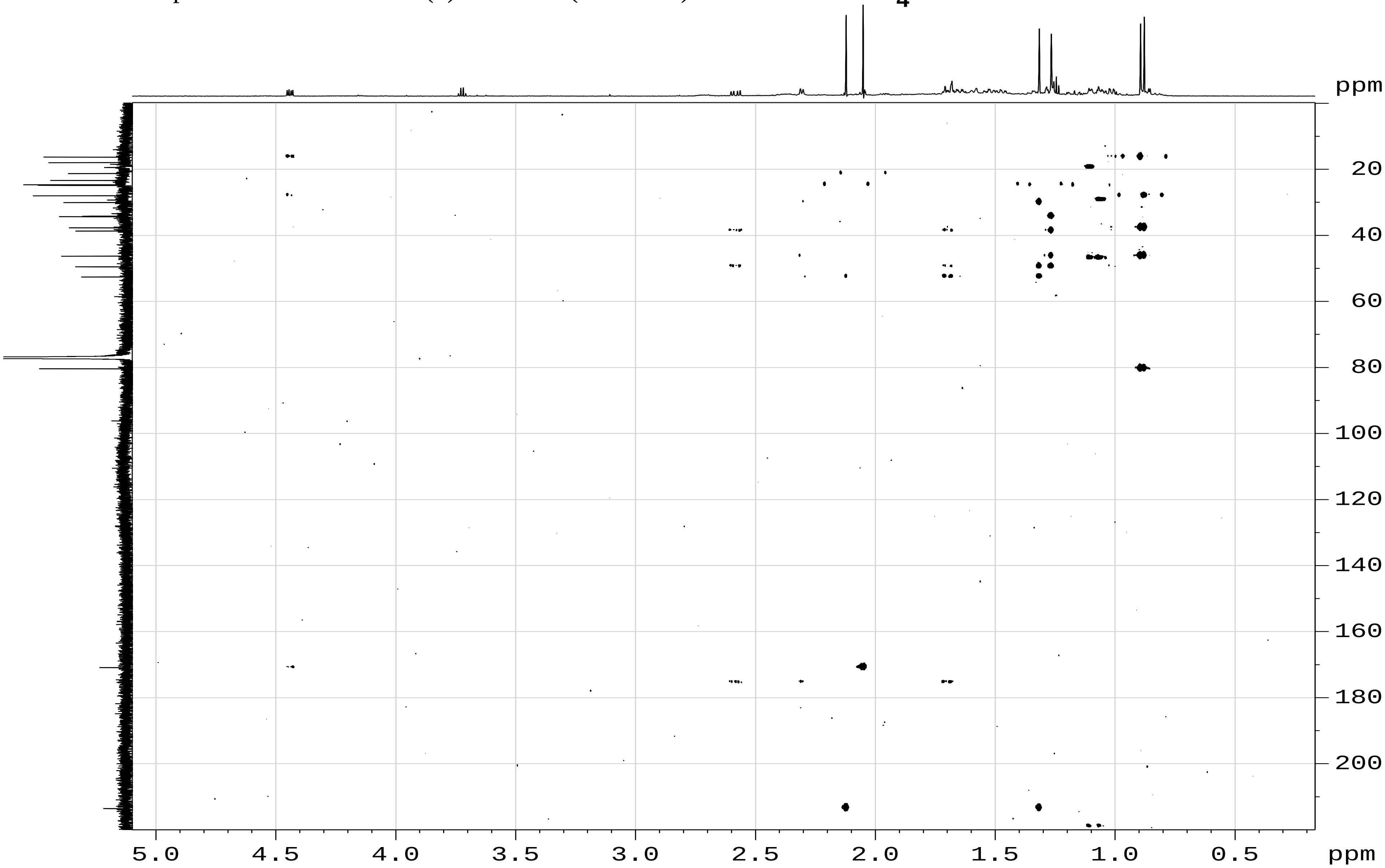

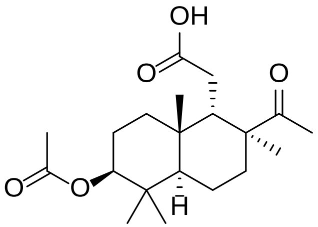

4

S33 COSY Spectrum of Stellettin T (**4**) in $CDCl_3$ (700 MHz)

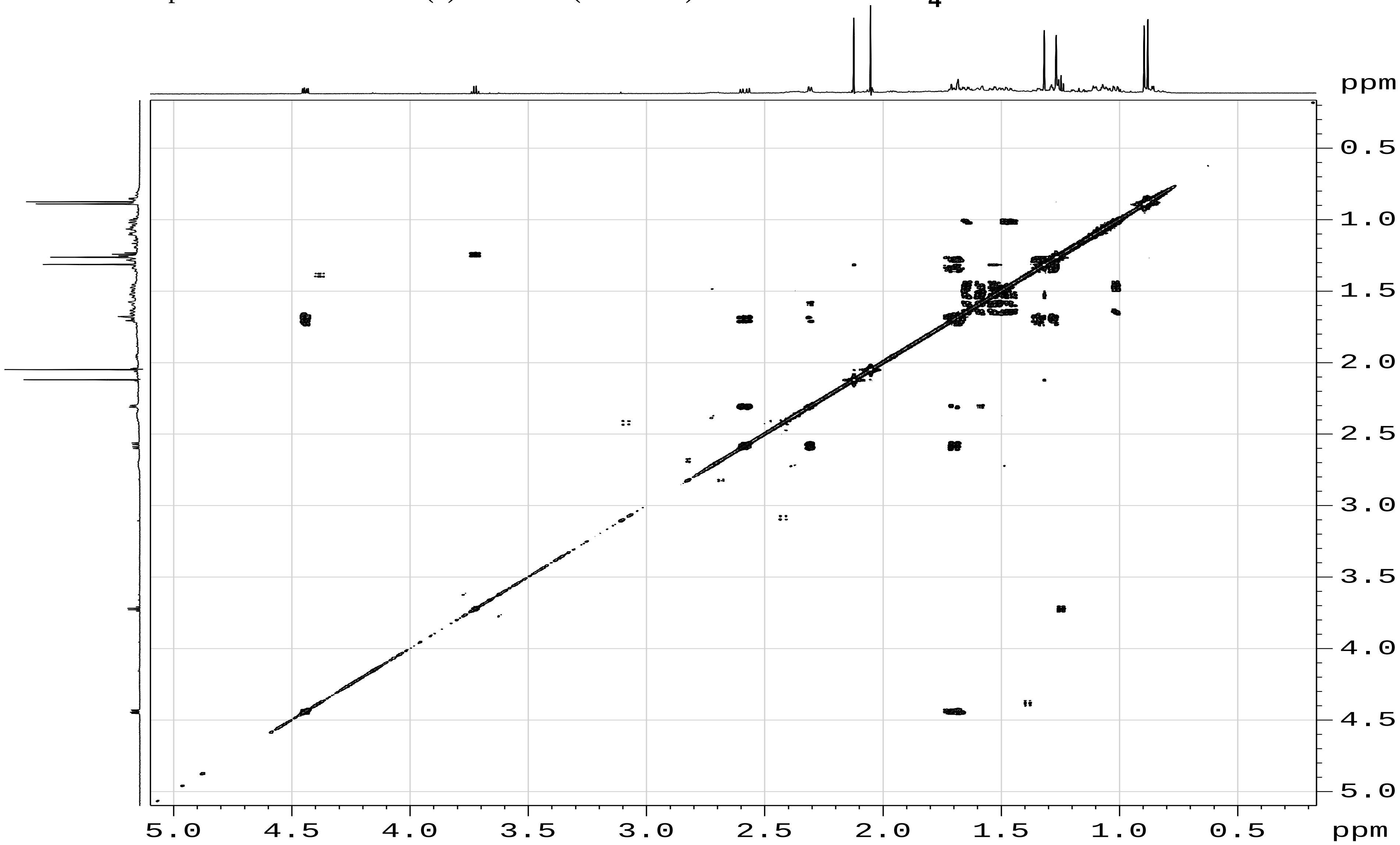

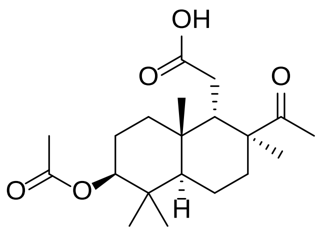

4

S34 ROESY Spectrum of Stellettin T (**4**) in $CDCl_3$ (700 MHz)

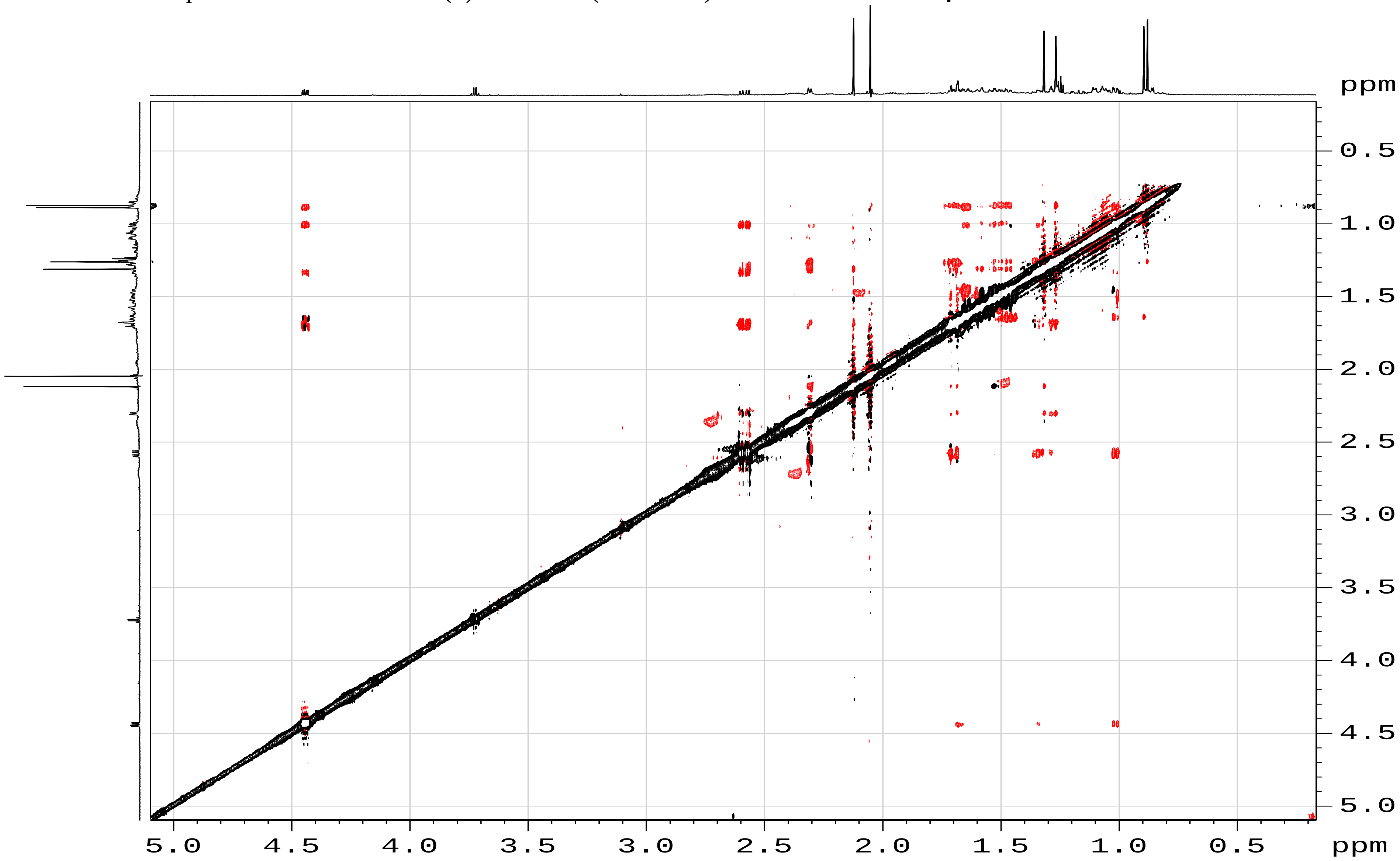

S35 ECD Spectrum of Stellettin T (**4**) in EtOH

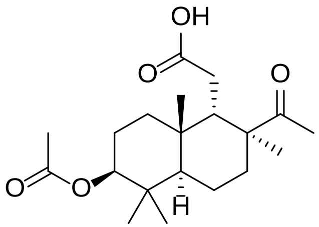

4

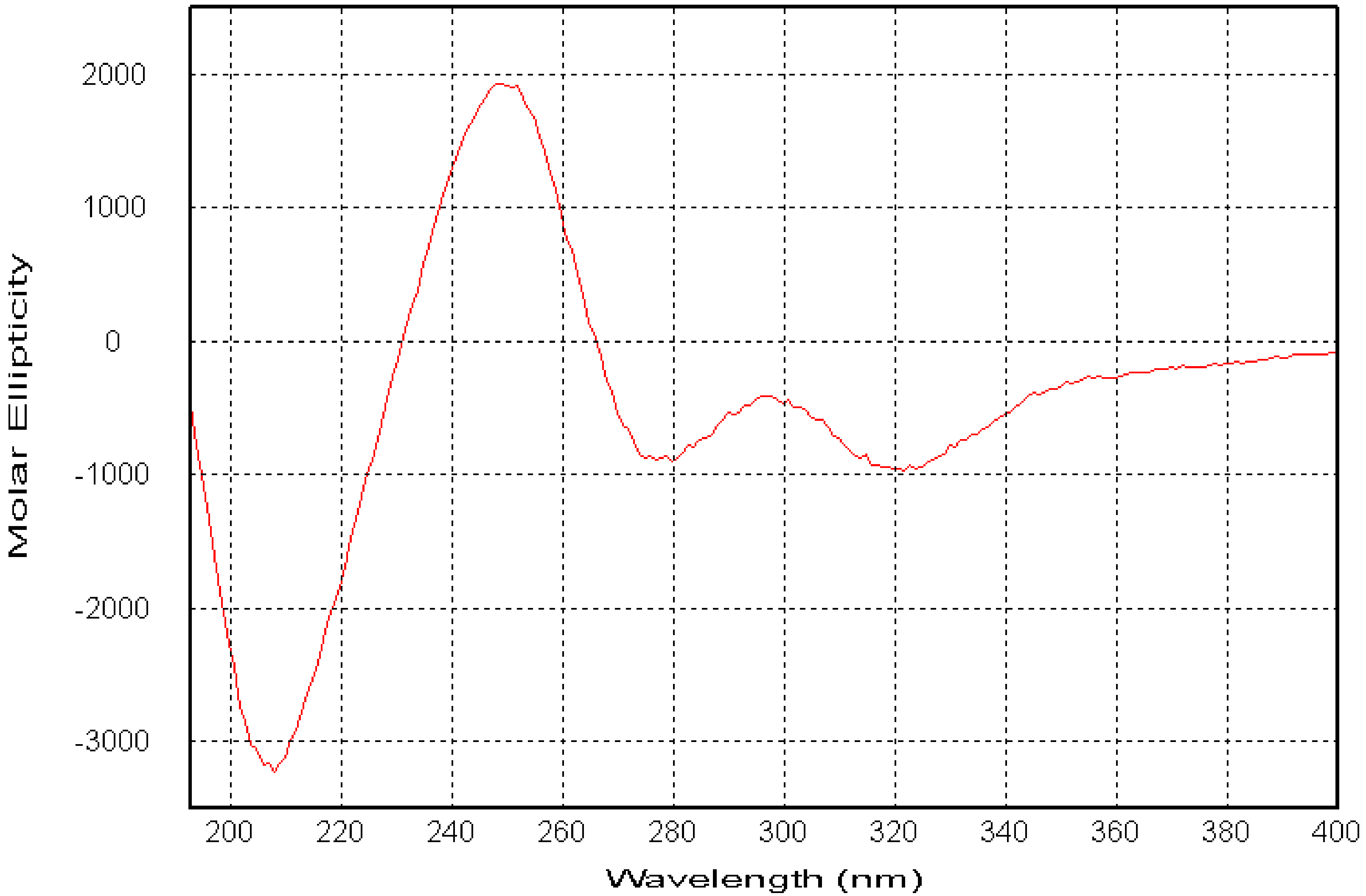

S36 HRESIMS and MS/MS Spectra (Negative Ion Mode) of Stellettin U (**5**)

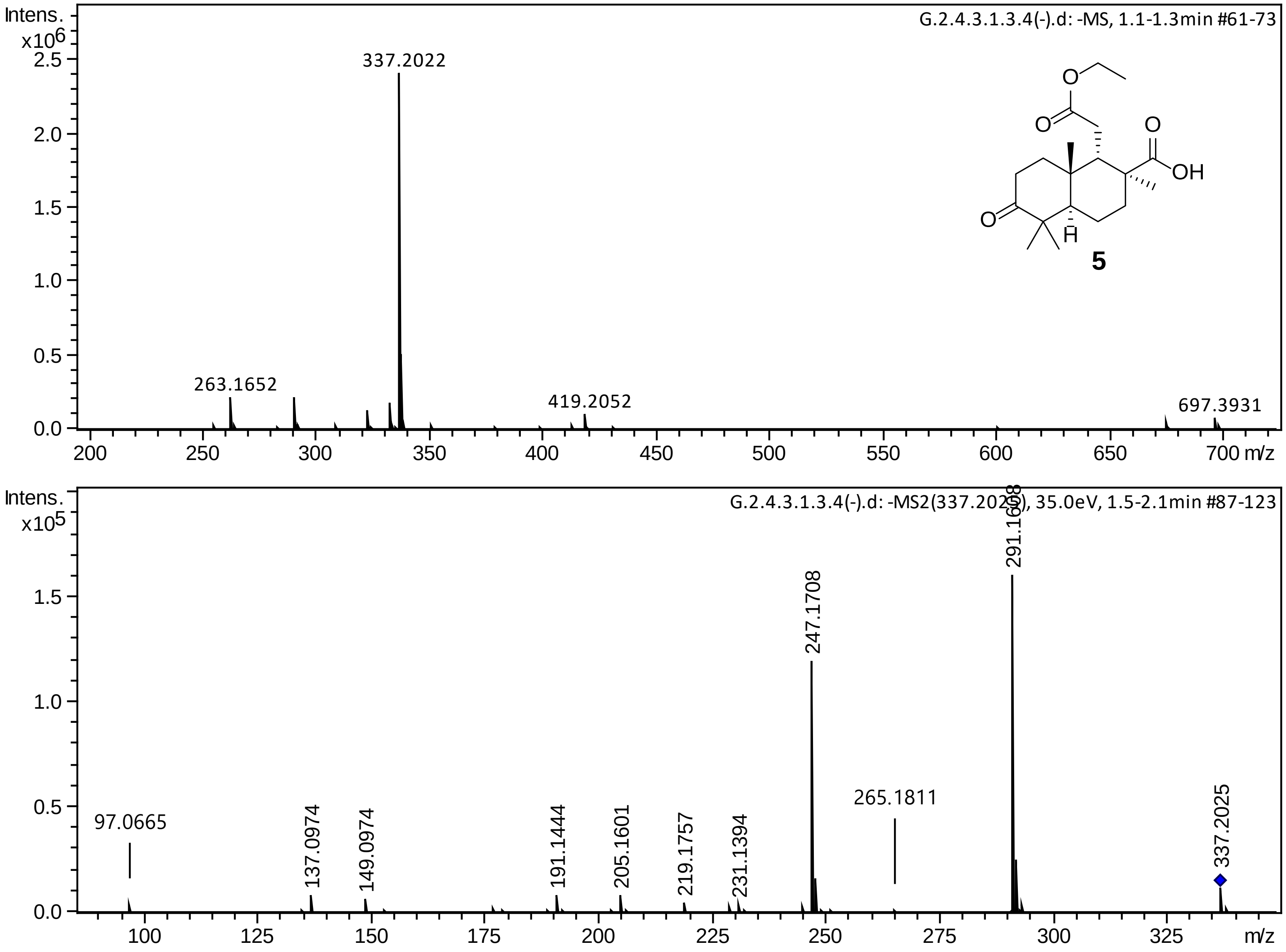

S37 ^{1}H NMR Spectrum of Stellettin U (**5**) in $CDCl_3$ (700 MHz)

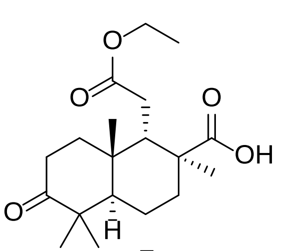

5

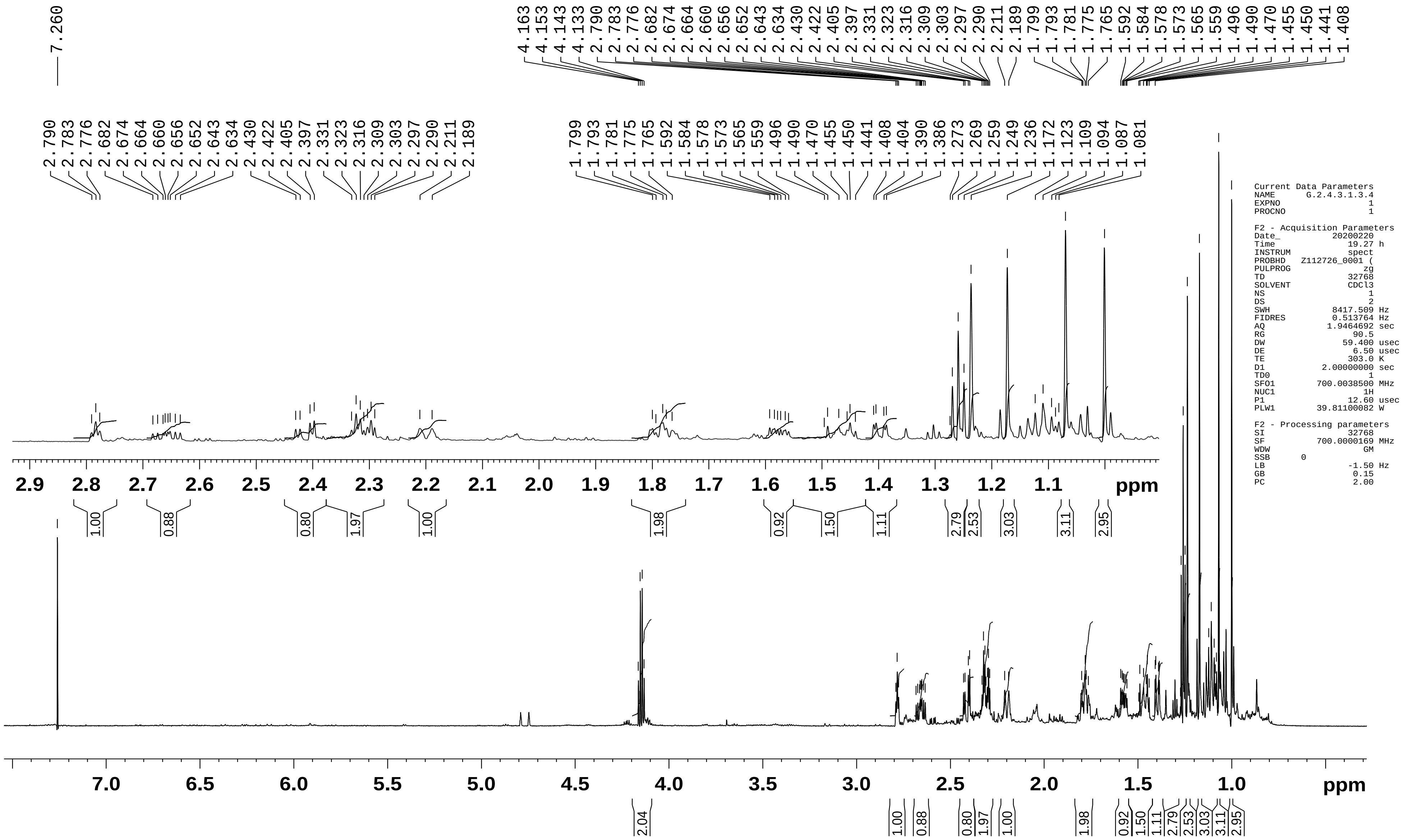

S38 ^{13}C NMR Spectrum of Stellettin U (**5**) in $CDCl_3$ (176 MHz)

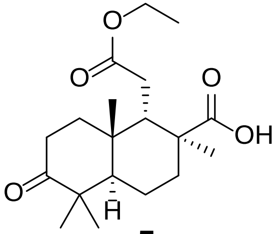

5

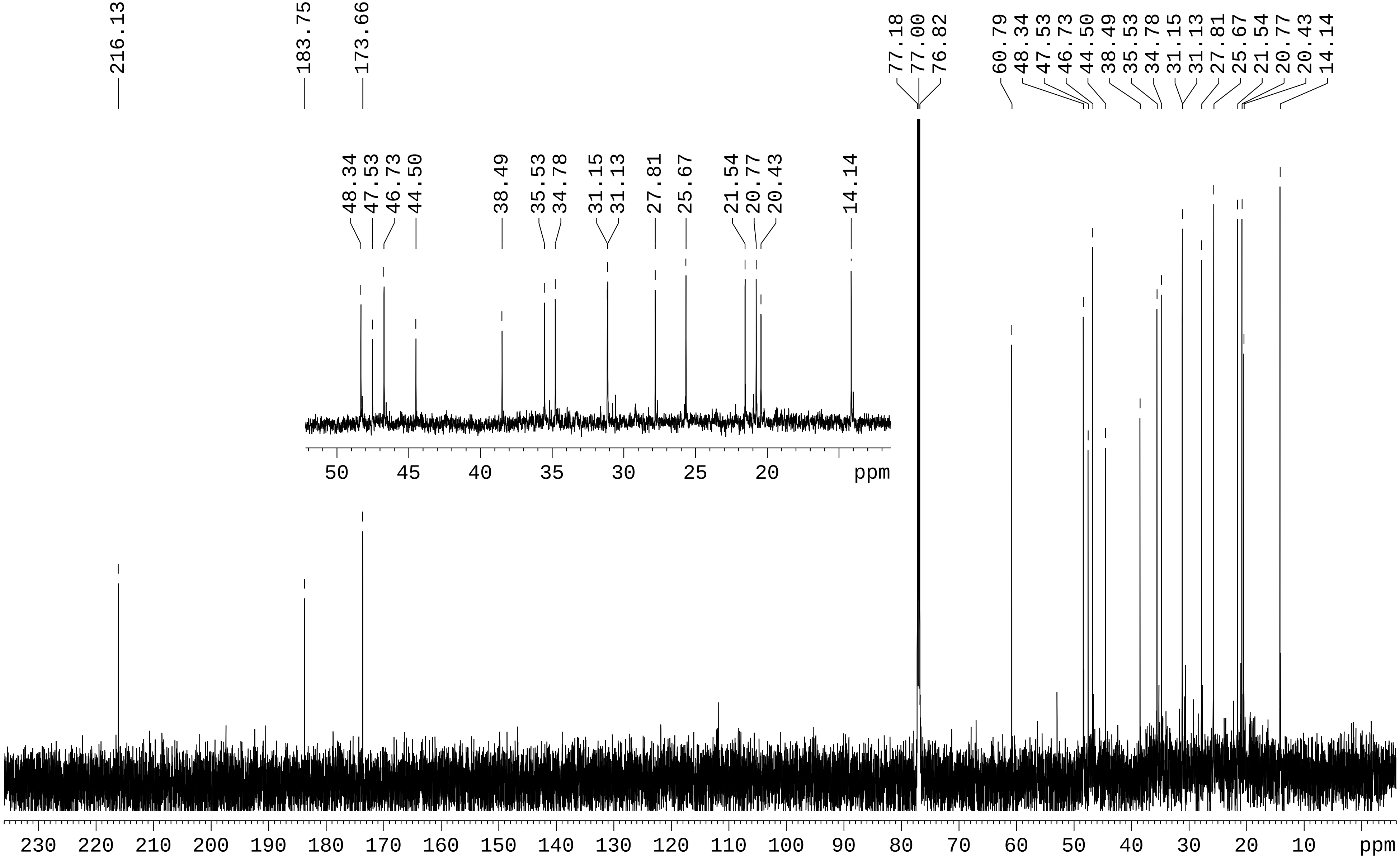

S39 DEPT Spectrum of Stellettin U (**5**) in $CDCl_3$ (176 MHz)

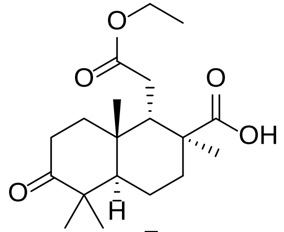

5

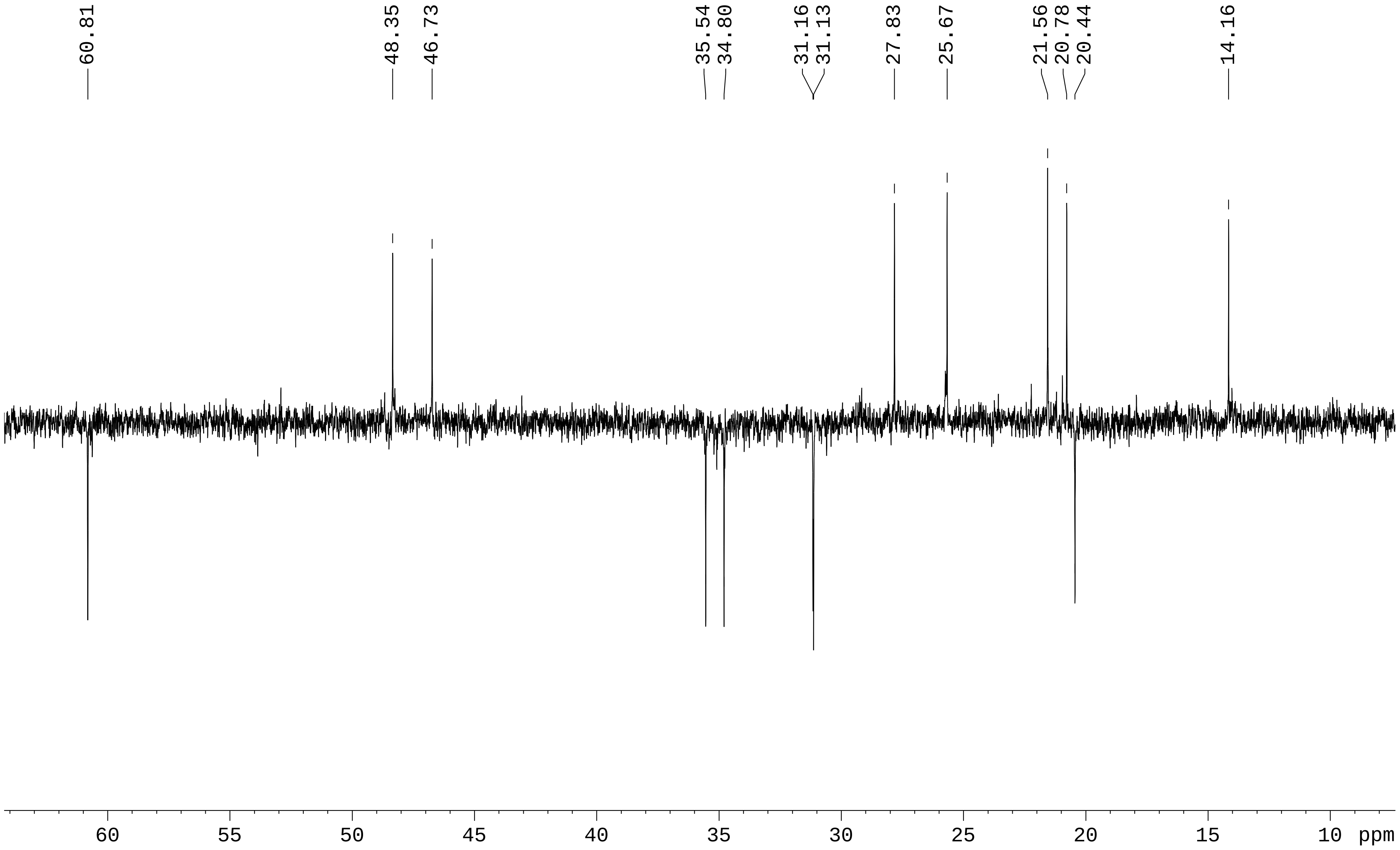

S40 HSQC Spectrum of Stellettin U (**5**) in $CDCl_3$ (700 MHz)

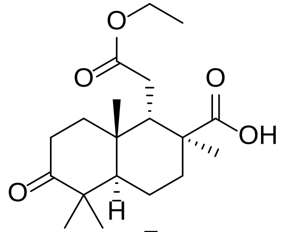

5

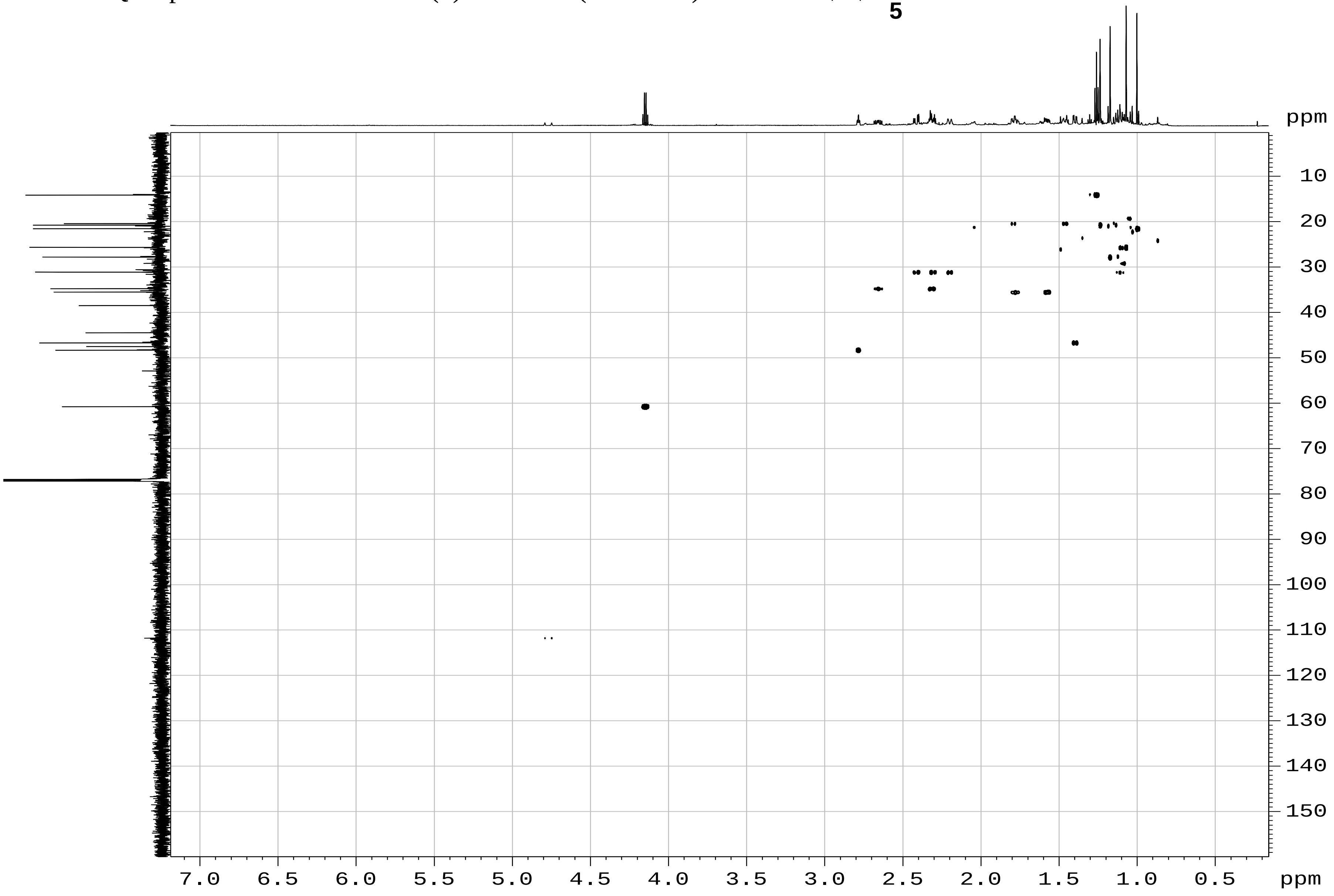

S41 HMBC Spectrum of Stellettin U (**5**) in $CDCl_3$ (700 MHz)

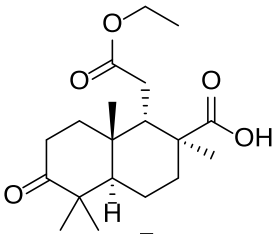

5

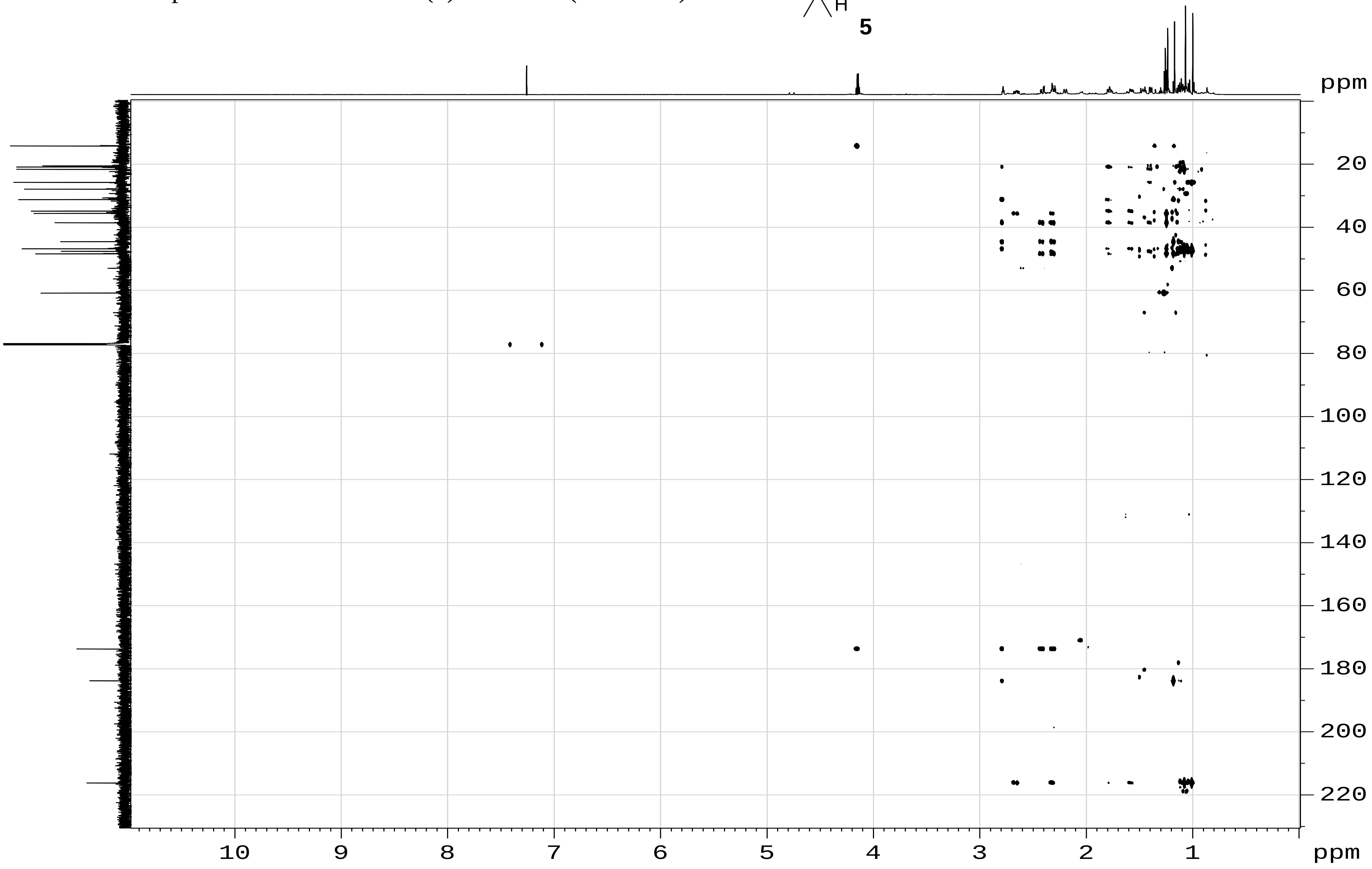

S42 COSY Spectrum of Stellettin U (**5**) in $CDCl_3$ (700 MHz)

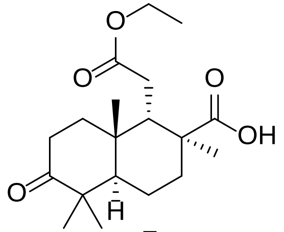

5

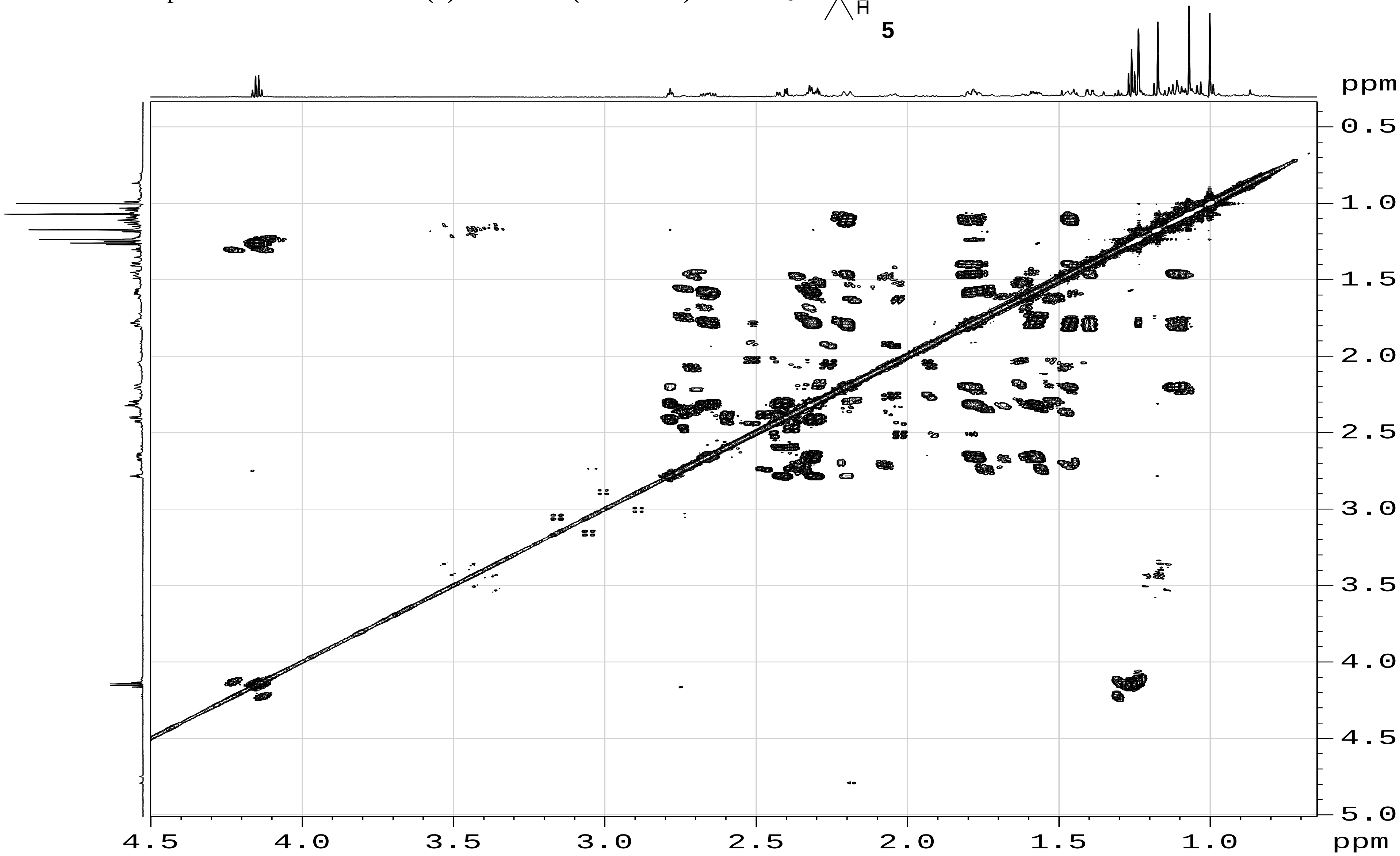

S43 ROESY Spectrum of Stellettin U (**5**) in $CDCl_3$ (700 MHz)

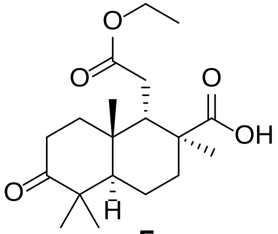

5

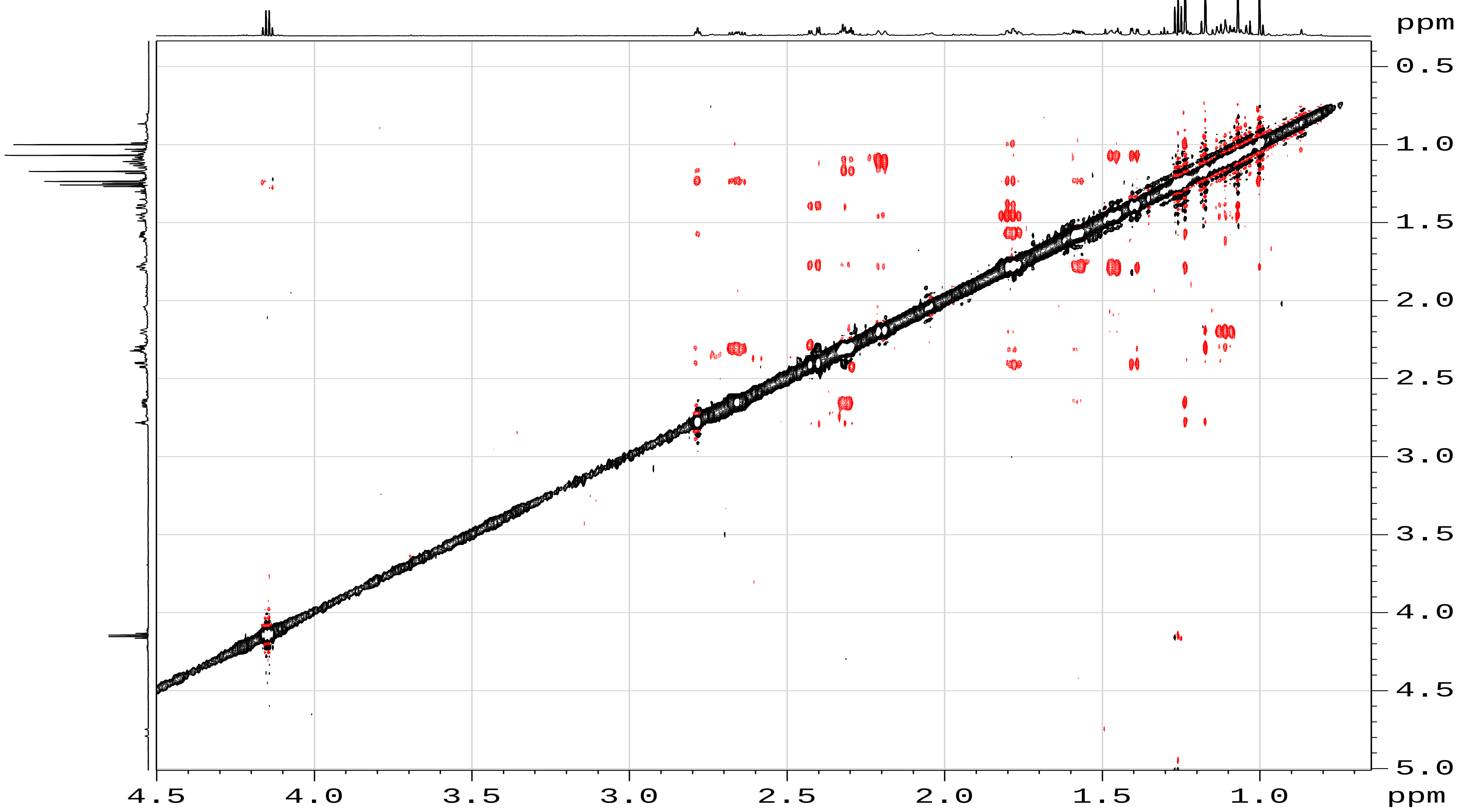

S44 ECD Spectrum of Stellettin U (**5**) in EtOH

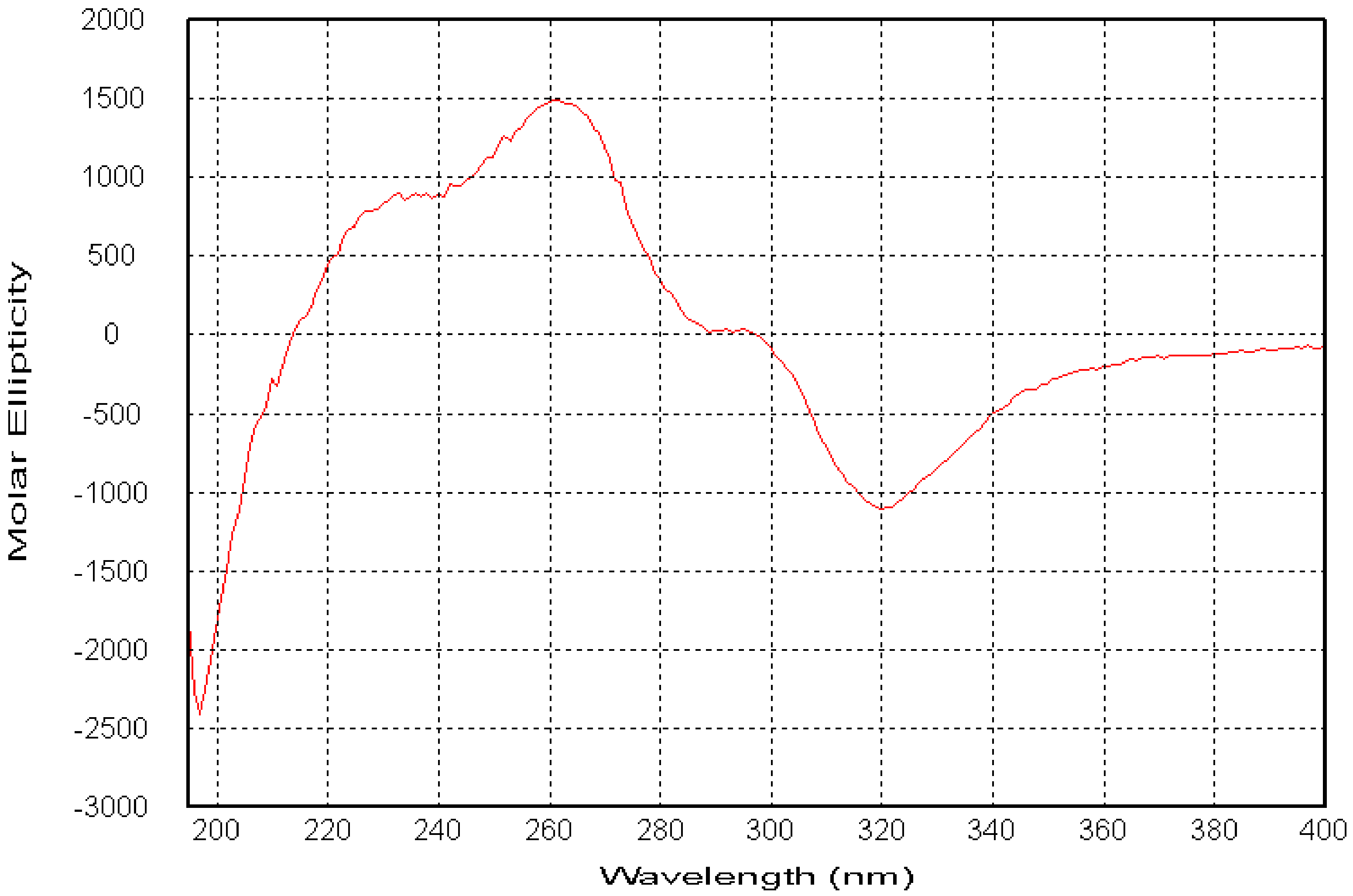

S45 HRESIMS and MS/MS Spectra (Negative Ion Mode) of Stellettin V (**6**)

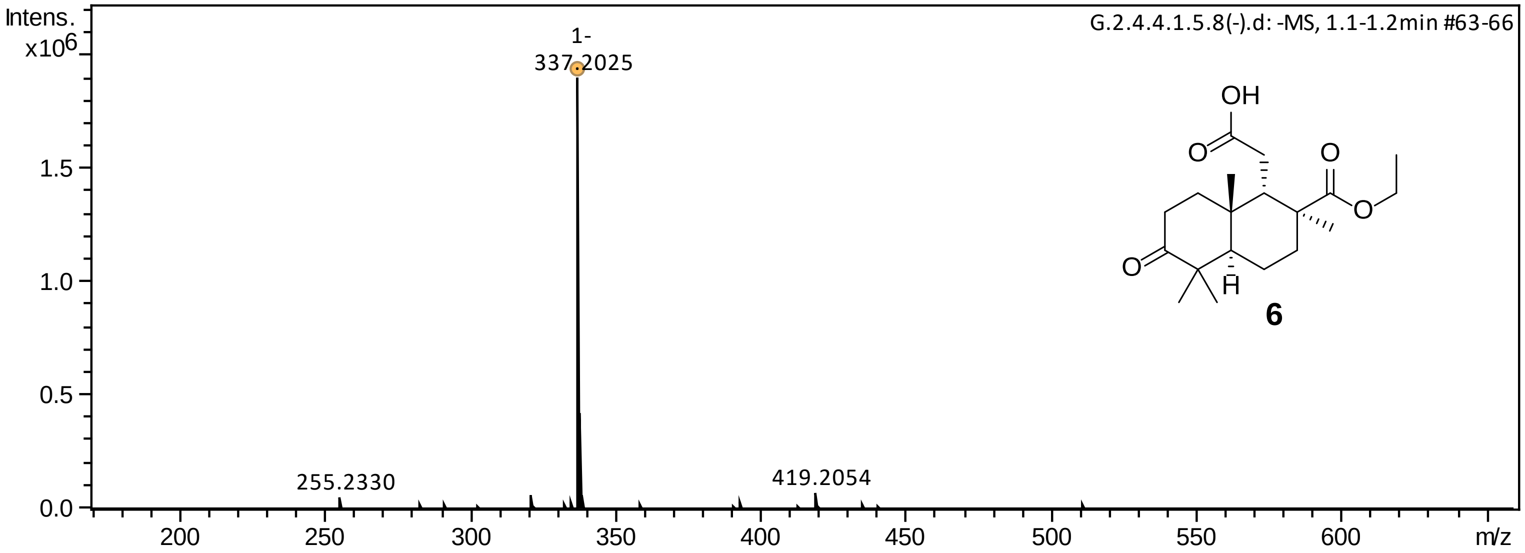

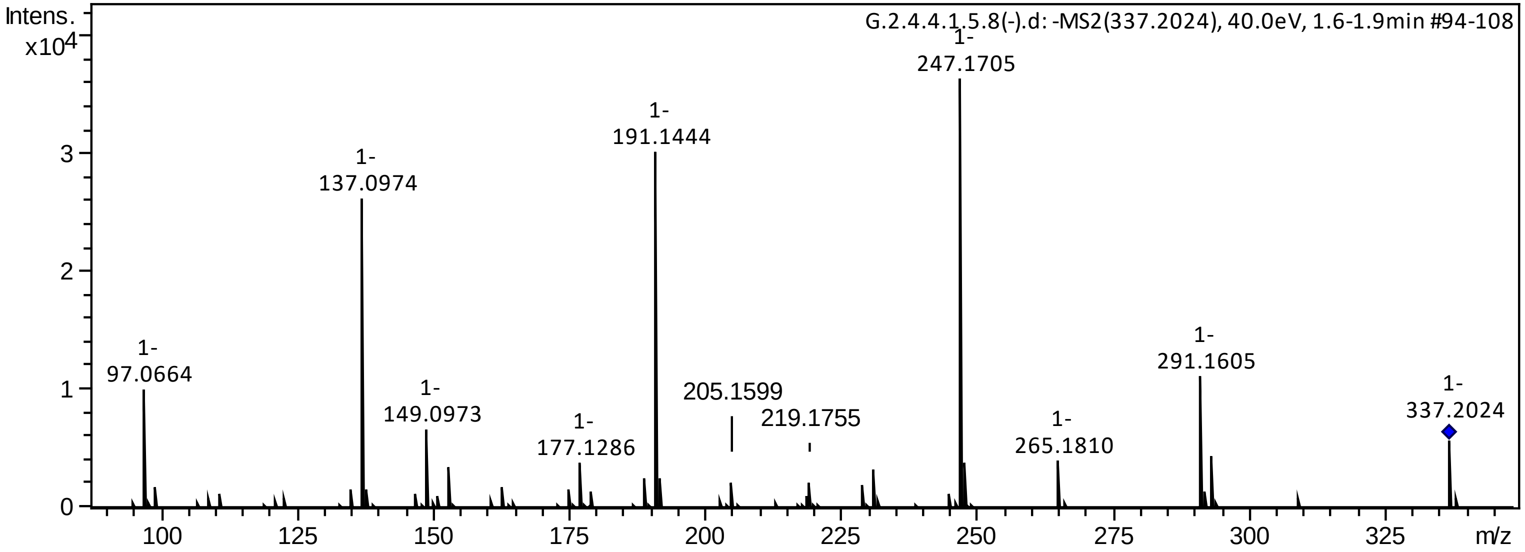

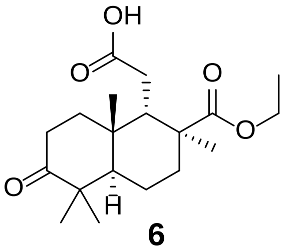

6

S46 ^{1}H NMR Spectrum of Stellettin V (**6**) in $CDCl_3$ (700 MHz)

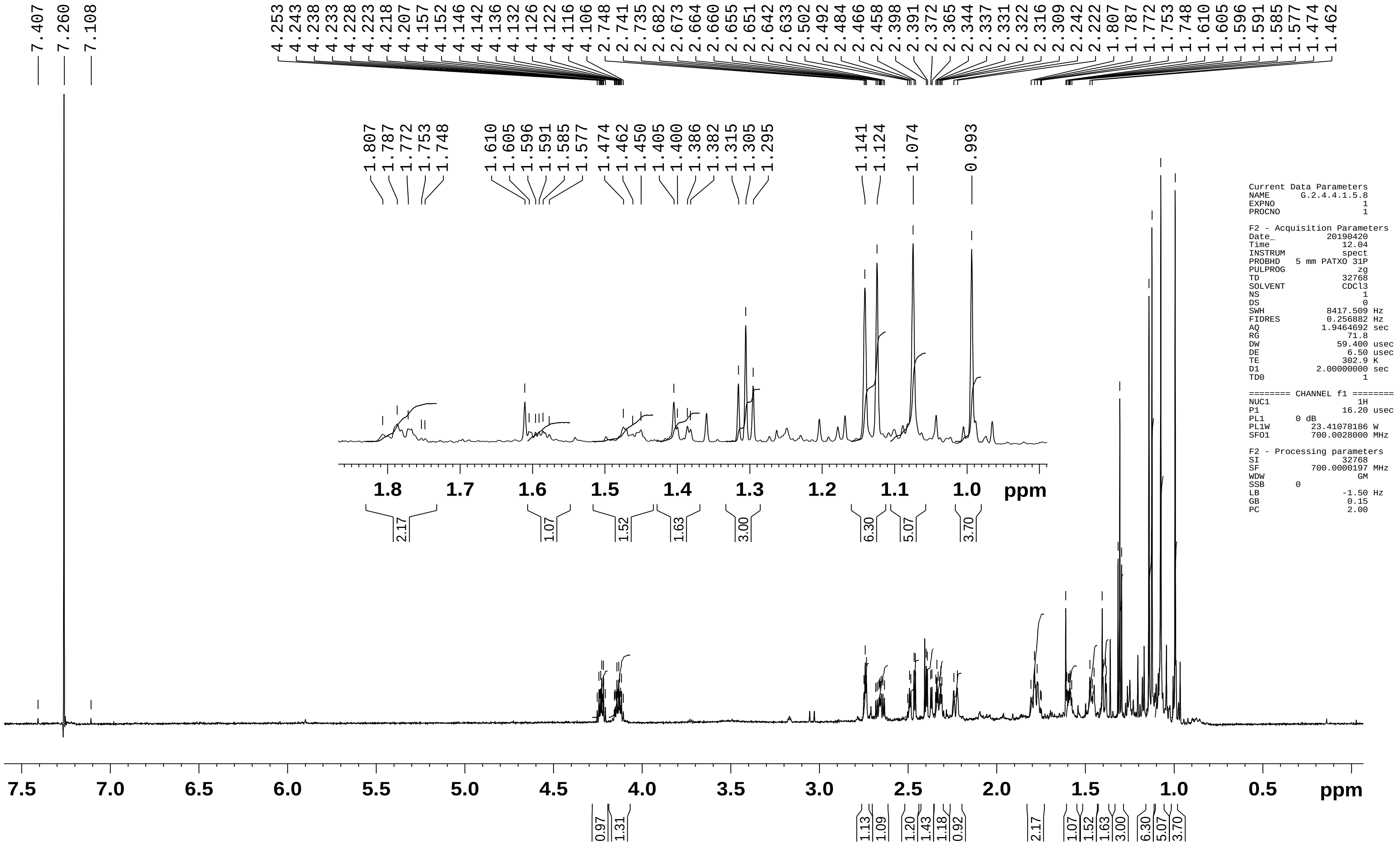

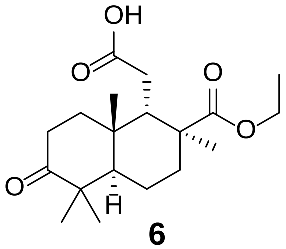

6

S47 ^{13}C NMR Spectrum of Stellettin V (**6**) in $CDCl_3$ (176 MHz)

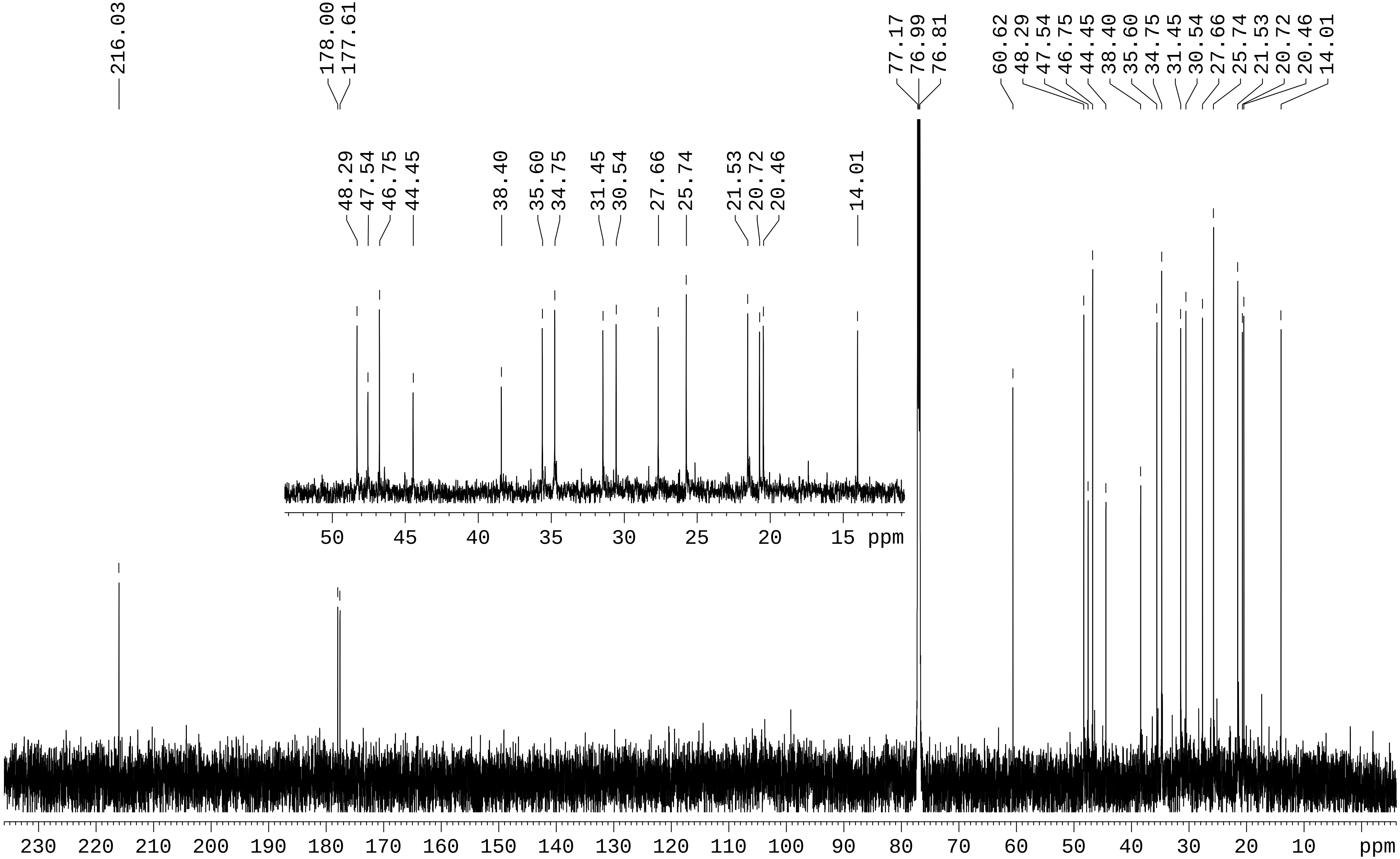

S48 DEPT Spectrum of Stellettin V (**6**) in $CDCl_3$ (176 MHz)

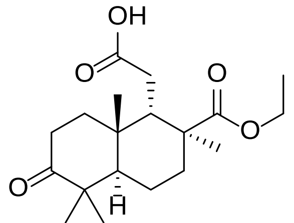

6

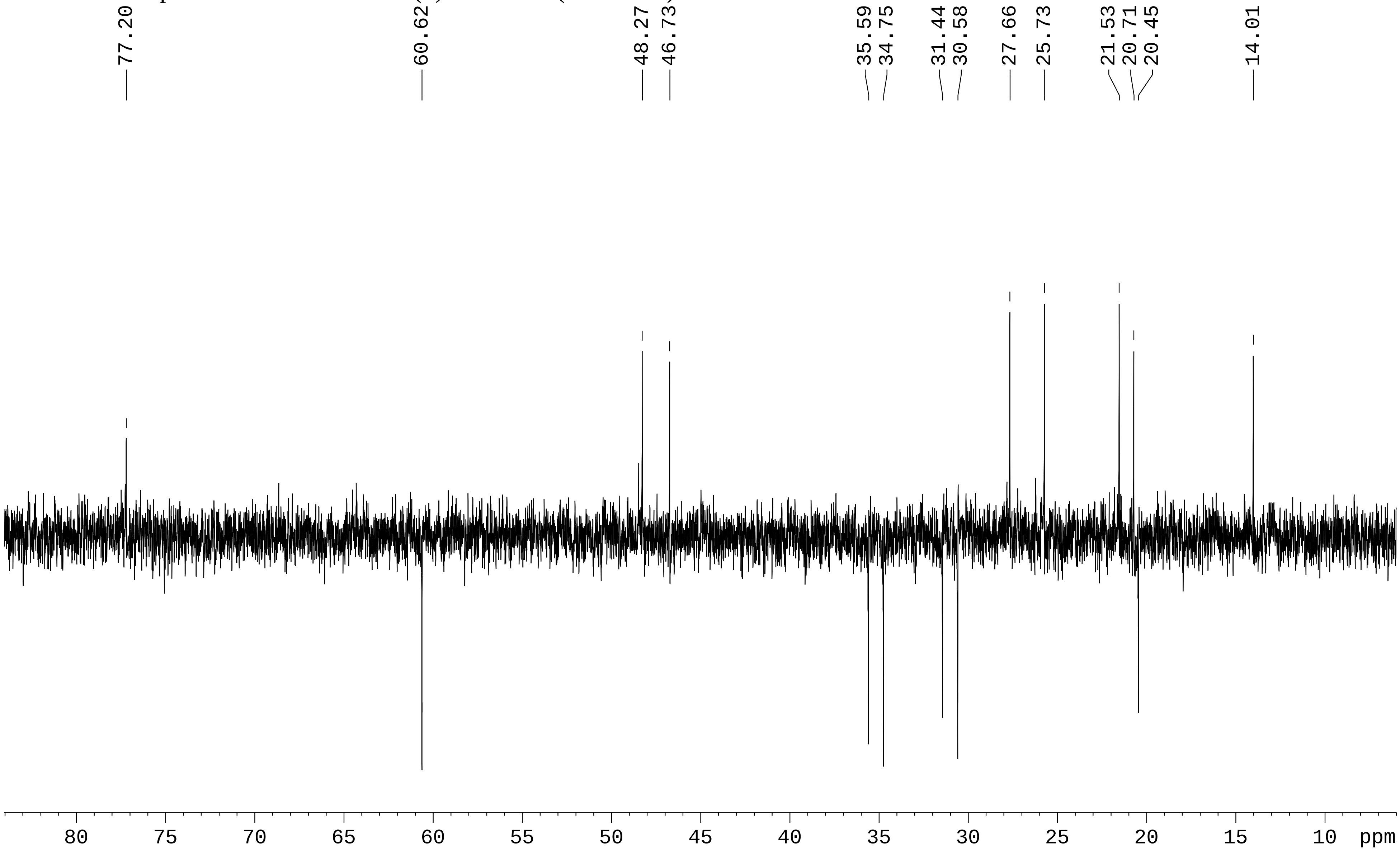

S49 HSQC Spectrum of Stellettin V (**6**) in $CDCl_3$ (700 MHz)

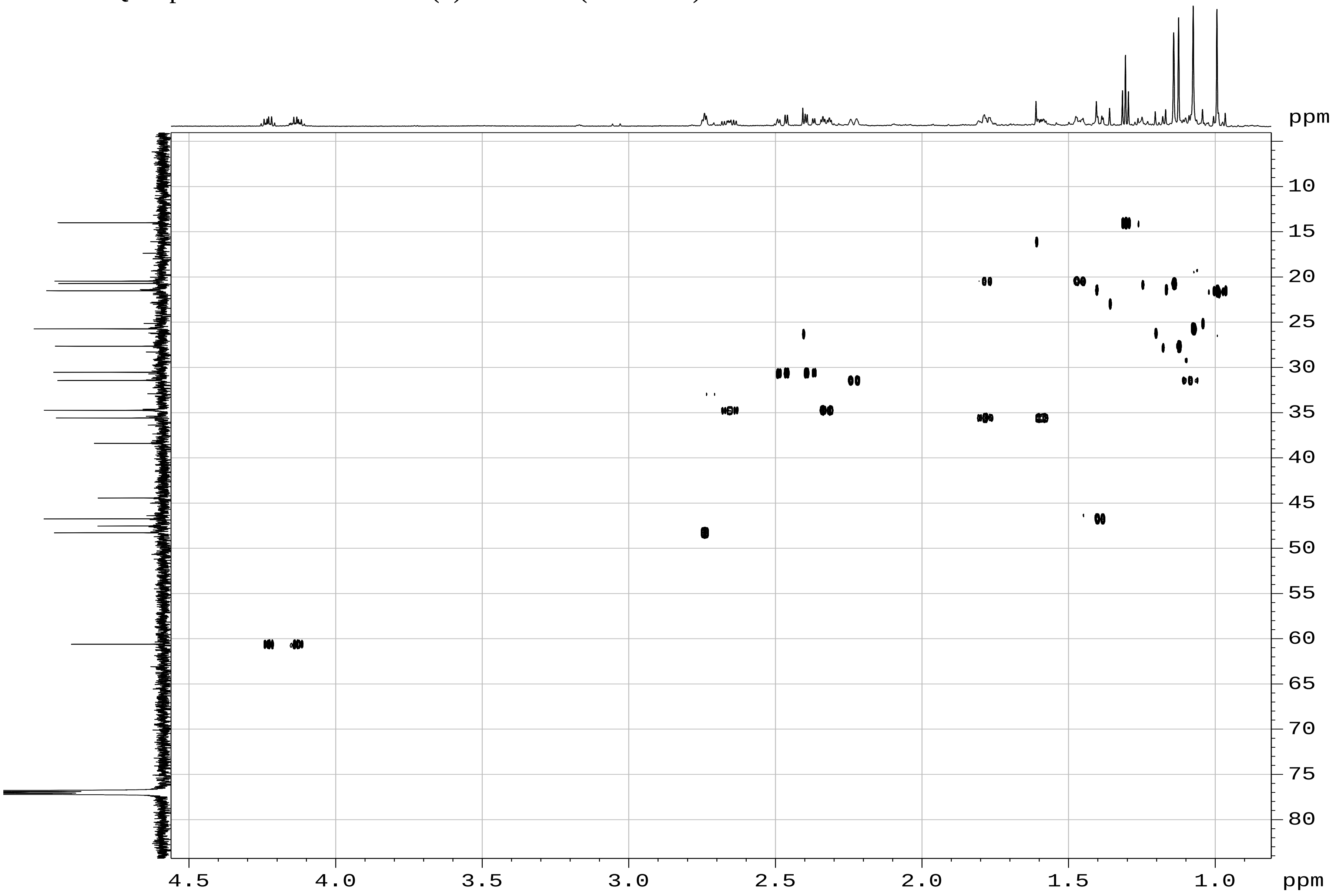

S50 HMBC Spectrum of Stellettin V (**6**) in $CDCl_3$ (700 MHz)

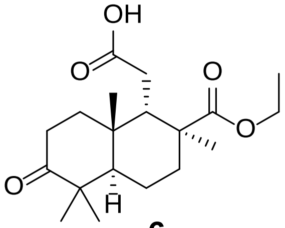

6

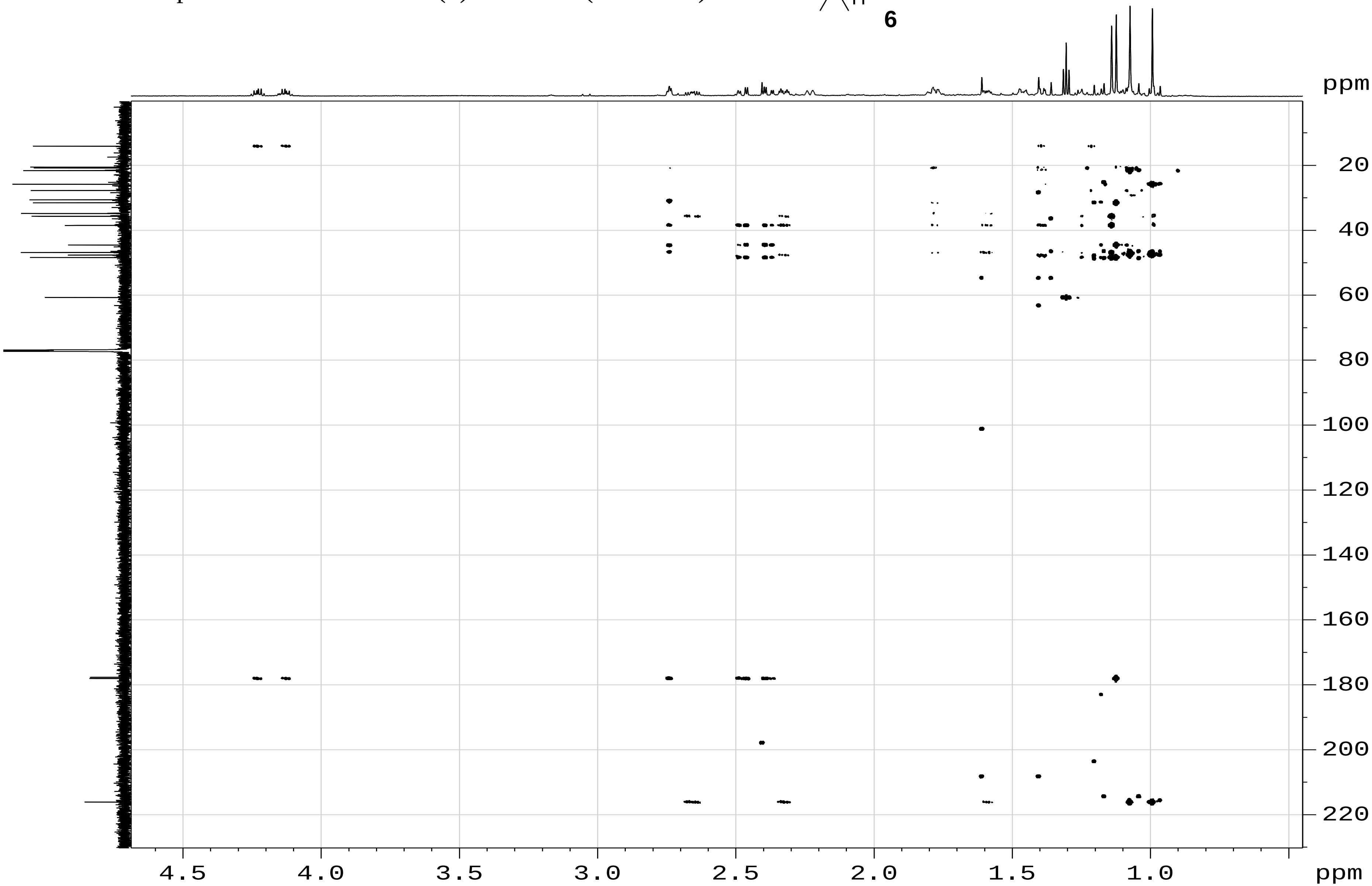

S51 COSY Spectrum of Stelletin V (**6**) in $CDCl_3$ (700 MHz)

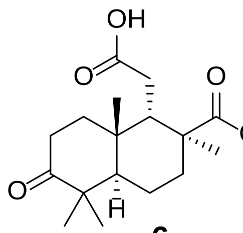

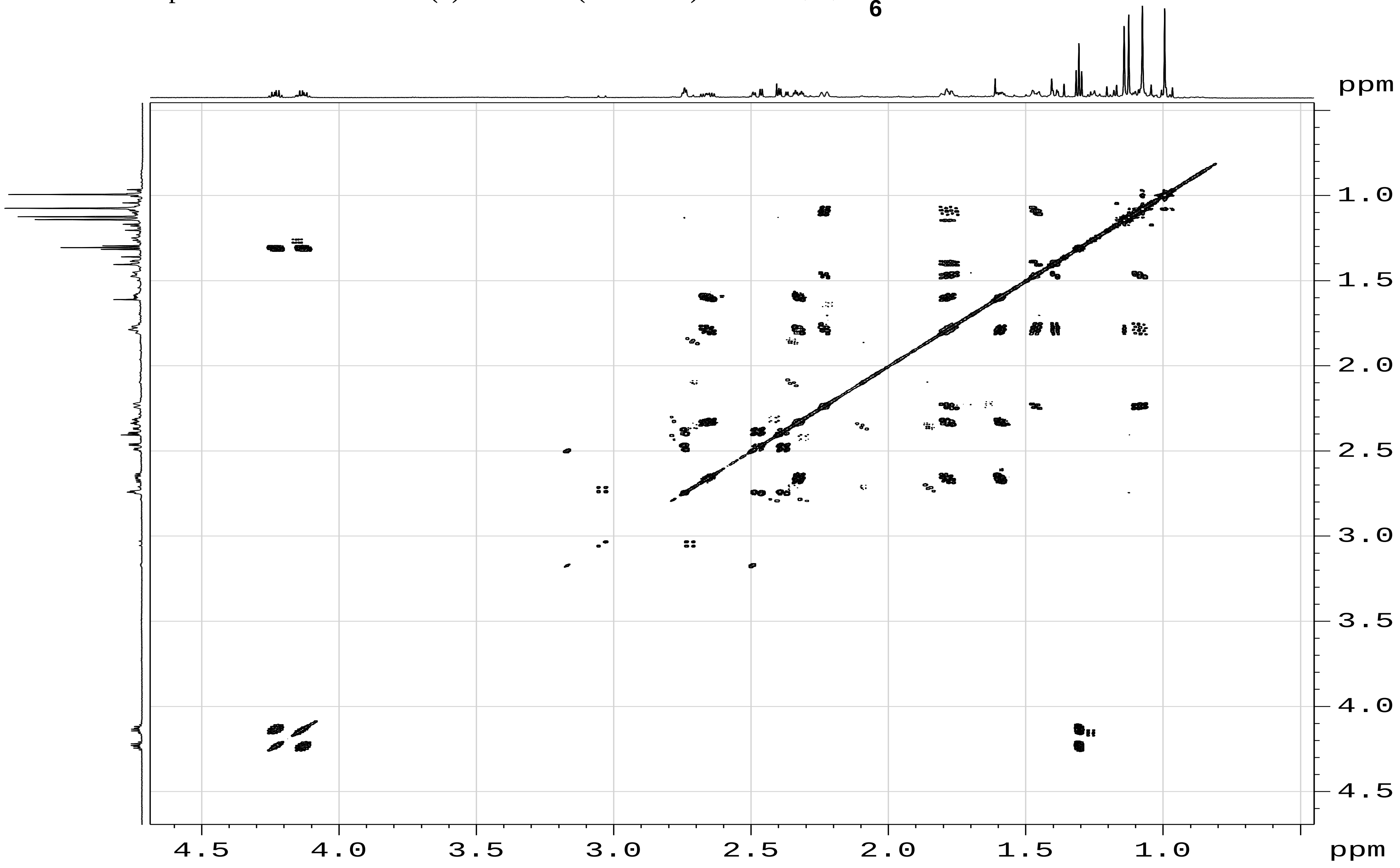

S52 ROESY Spectrum of Stellettin V (**6**) in $CDCl_3$ (700 MHz)

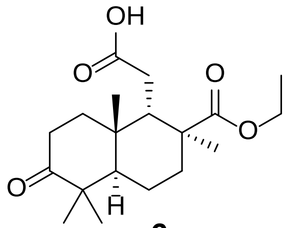

6

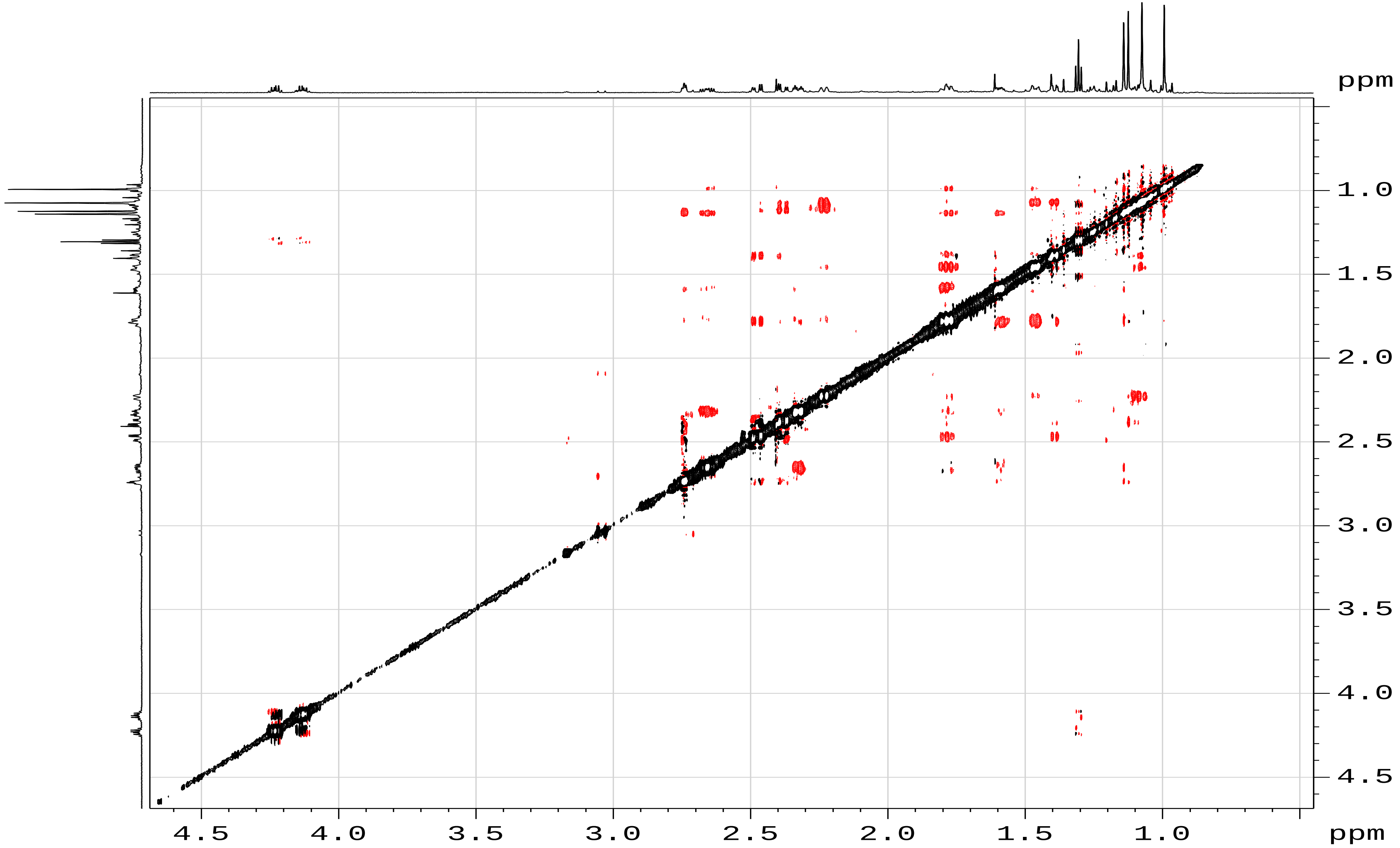

S53 ECD Spectrum of Stellettin V (**6**) in EtOH

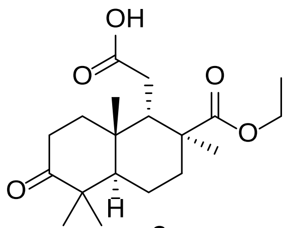

6

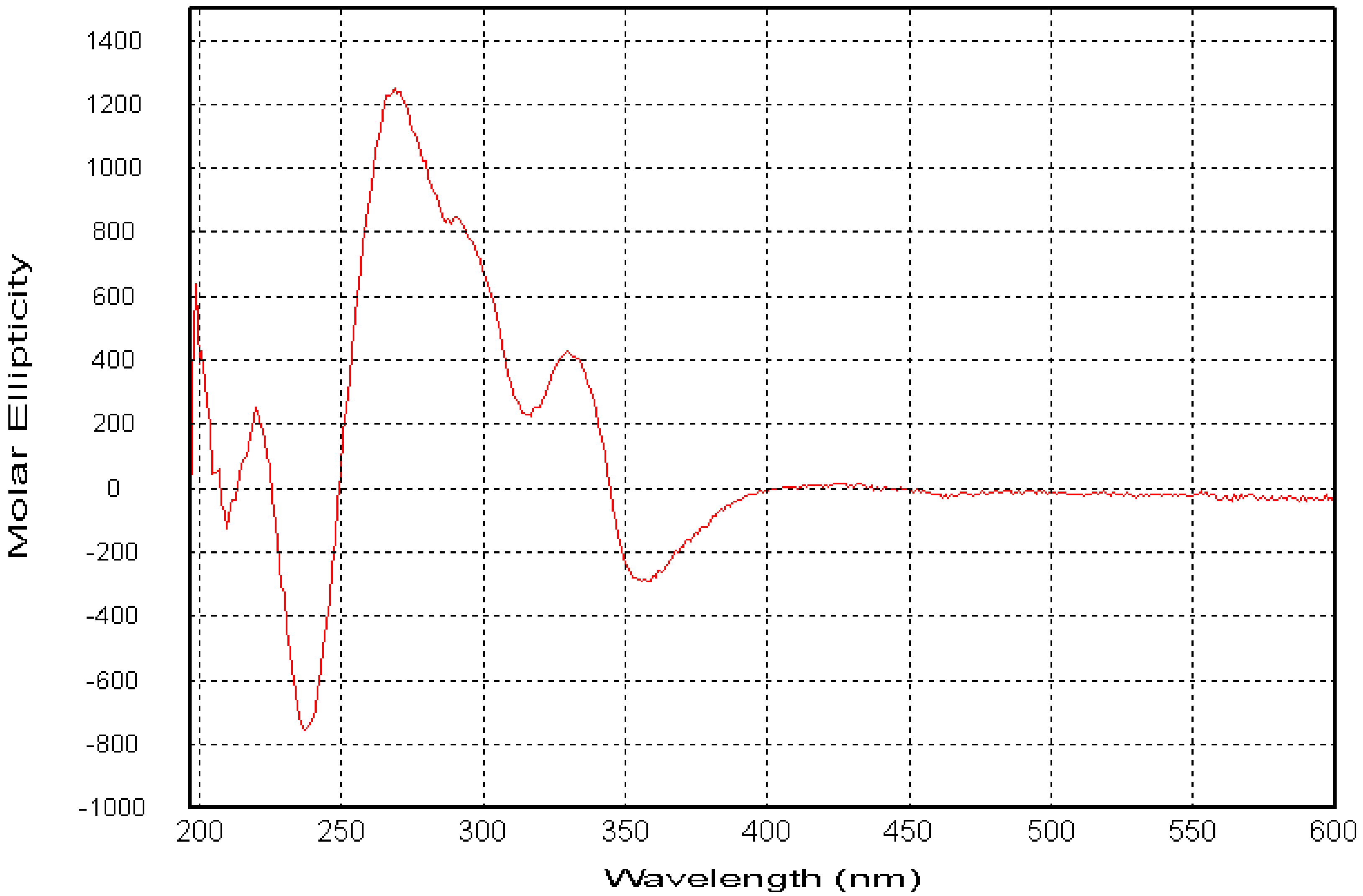

S54 HRESIMS and MS/MS Spectra (Negative Ion Mode) of Globostelletin K from *Stelletta* sp.

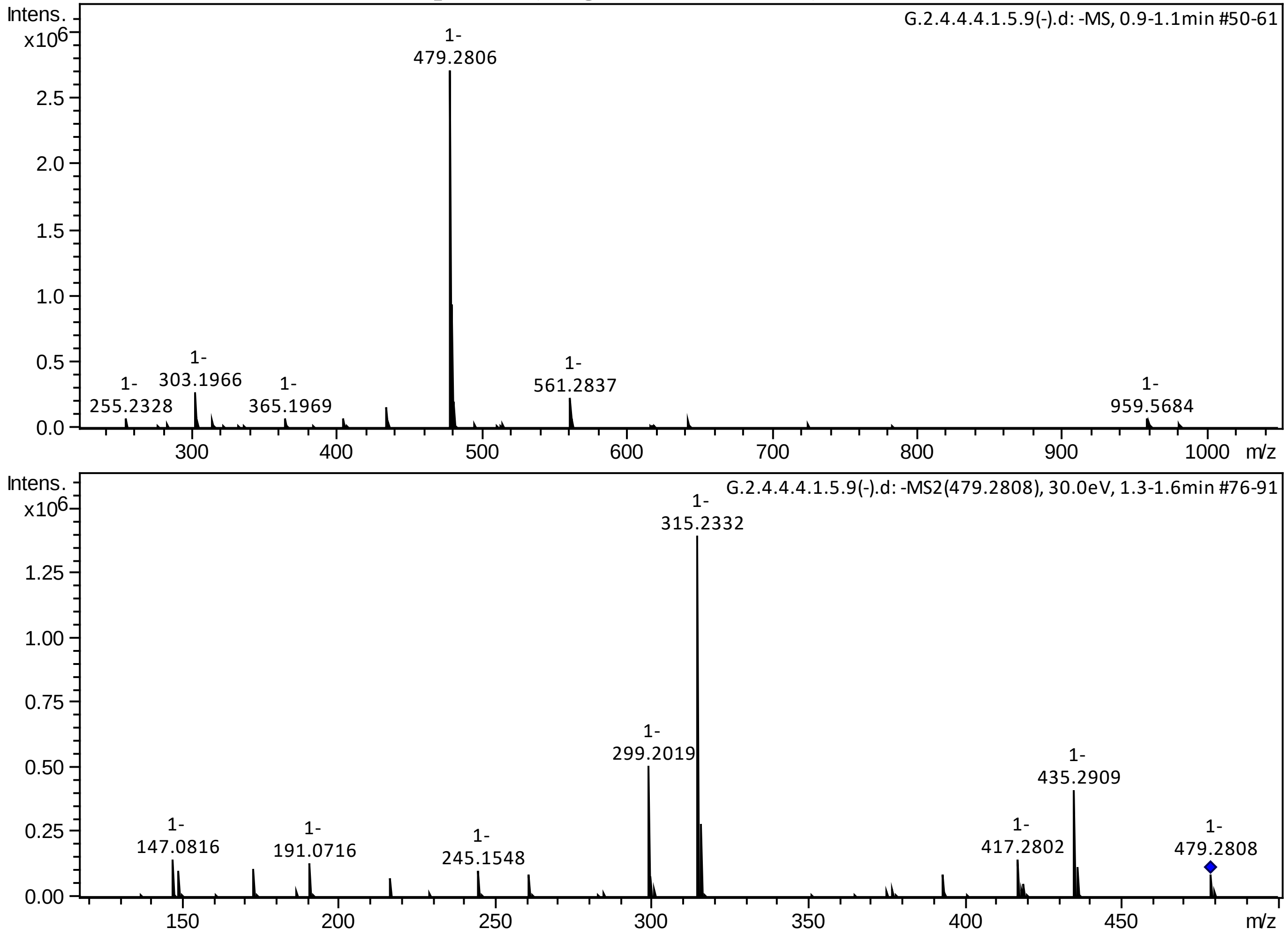

S55 ^{1}H NMR Spectrum of Globostelletin K from *Stelletta* sp. (700 MHz, $CDCl_3$)

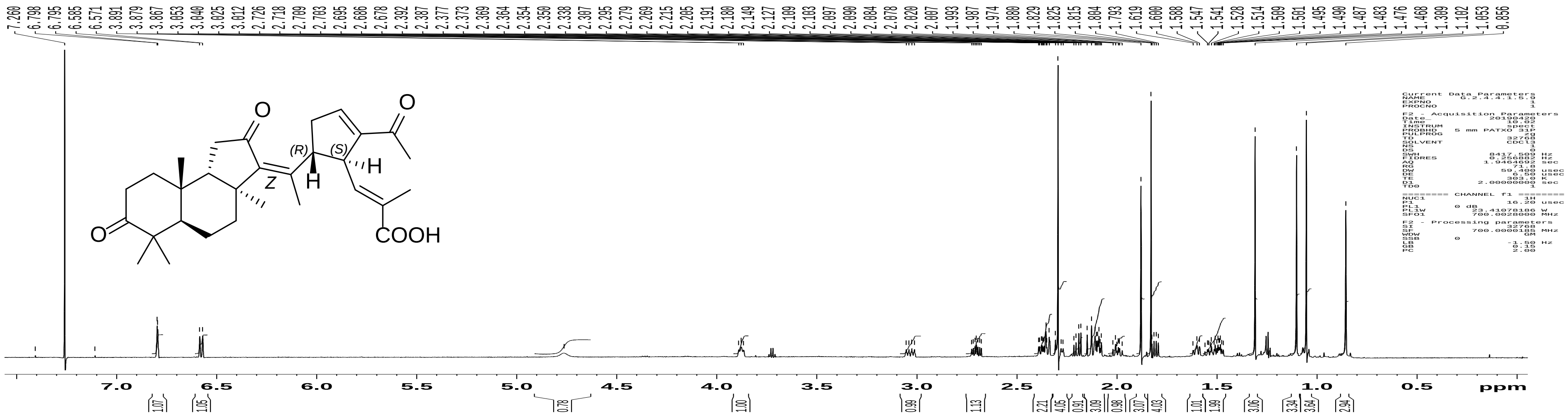

^{1}H NMR Spectrum of Globostelletin K from *Rhabdastrella globostellata*. (500 MHz, $CDCl_3$) [1][*]

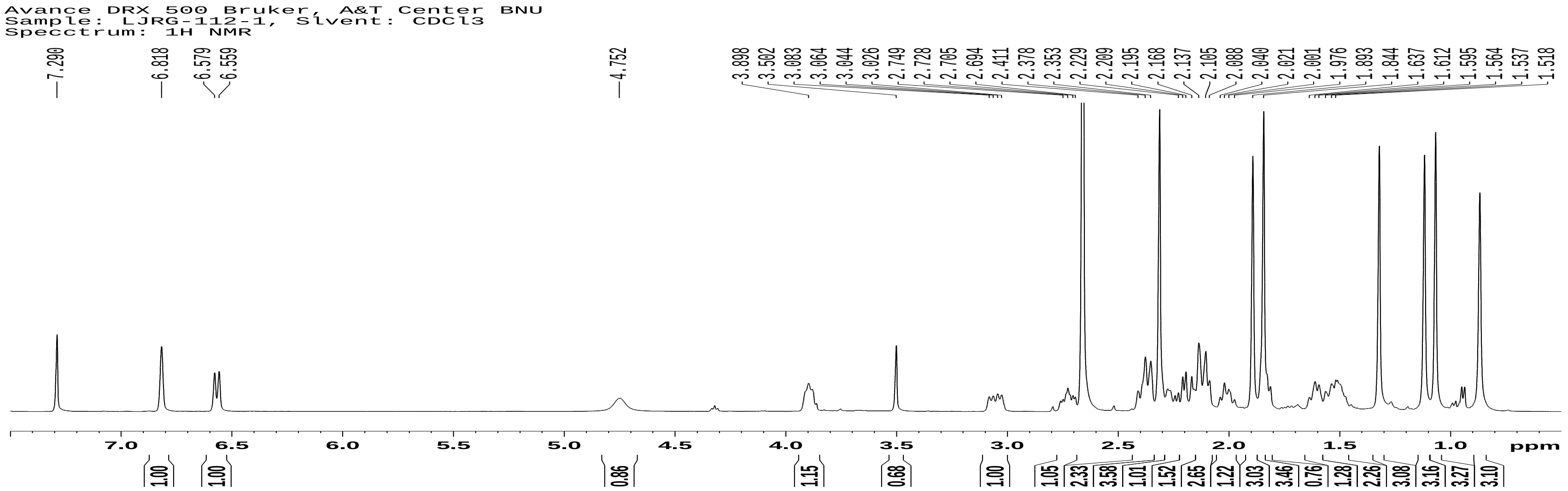

[*] [1] J. Li, H. Zhu, J. Ren, Z. Deng, N.J. de Voogd, P. Proksch, W. Lin Tetrahedron. 68 (2012) 559–565 (Supplementary data).

S56 ^{13}C NMR Spectrum of Globostelletin K from *Stelletta* sp. (176 MHz, $CDCl_3$)

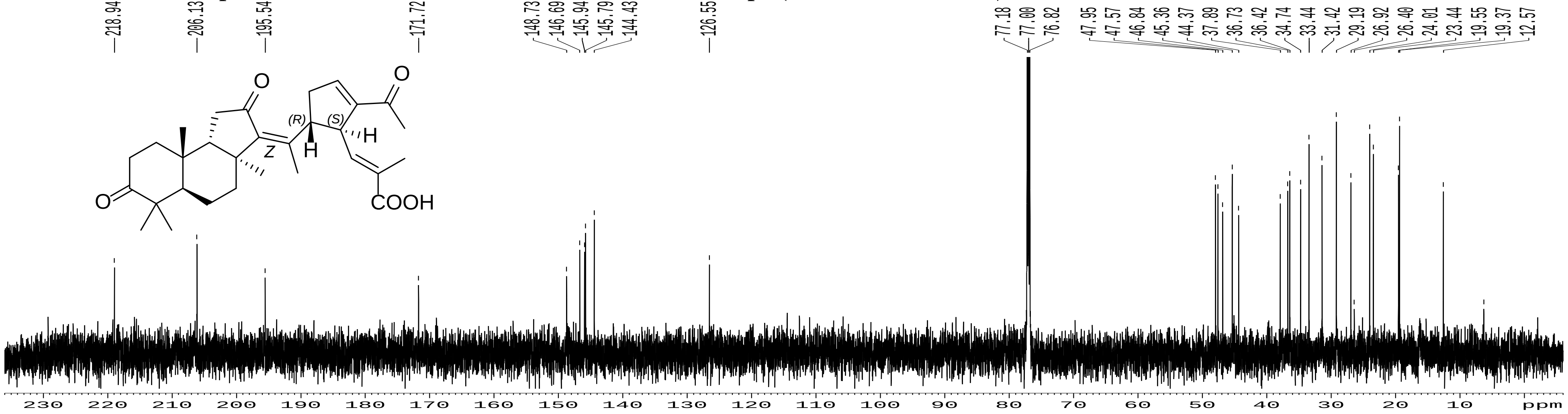

^{13}C NMR Spectrum of Globostelletin K from *Rhabdastrella globostellata*. (125 MHz, $CDCl_3$) [1]

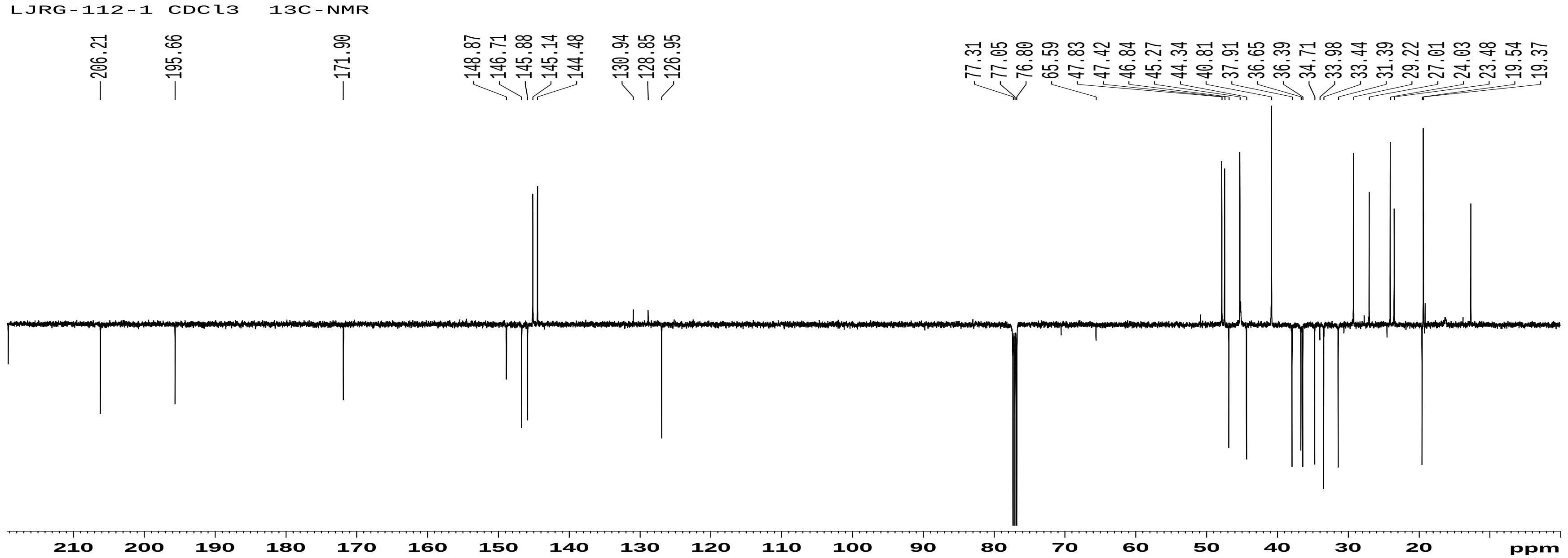

S57 ECD Spectrum of Globostelletin K from *Stelletta* sp. (EtOH)

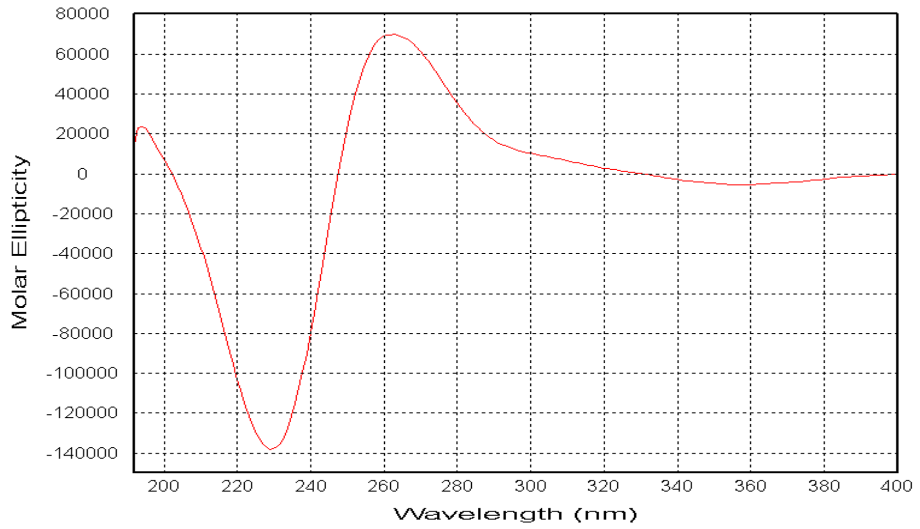

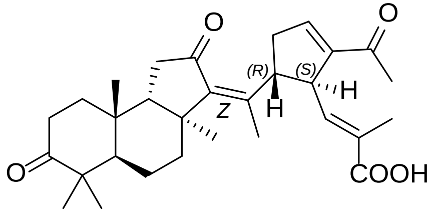

ECD Spectrum of Globostelletin K from *Rhabdastrella globostellata* [1]

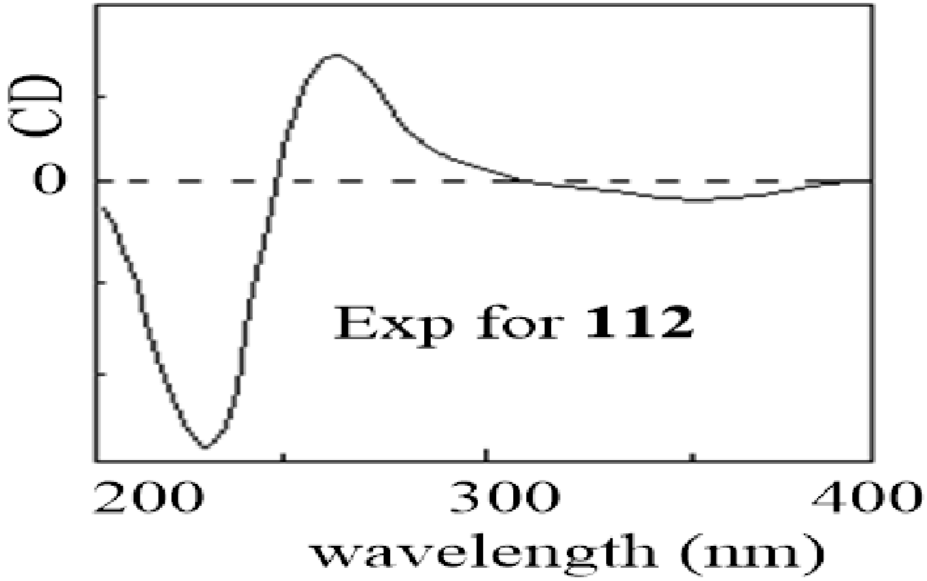

S58 HRESIMS and MS/MS Spectra (Negative Ion Mode) of Globostelletin M from *Stelletta* sp.

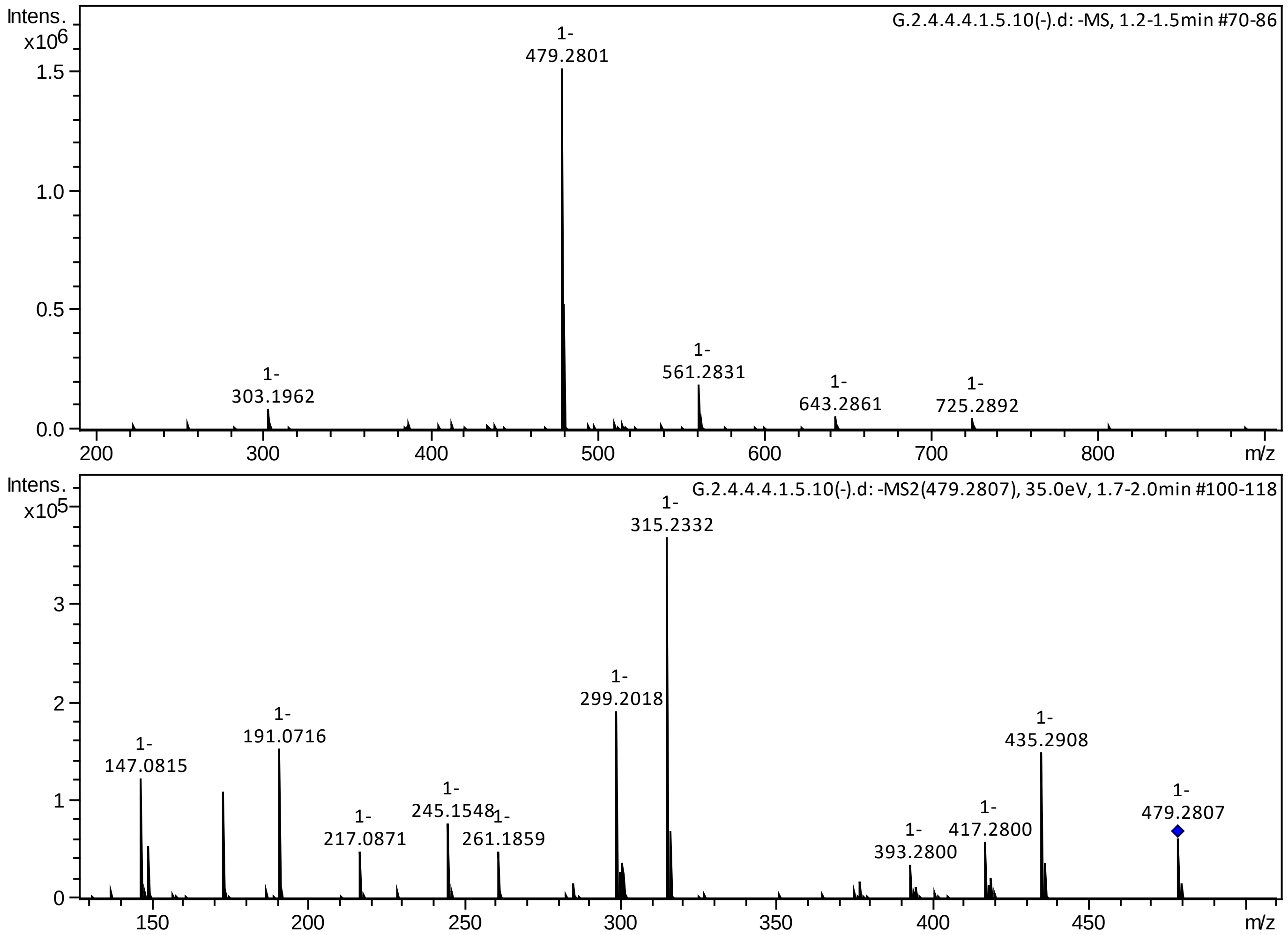

S59 ^{1}H NMR Spectrum of Globostelletin M from *Stelletta* sp. (700 MHz, $CDCl_3$)

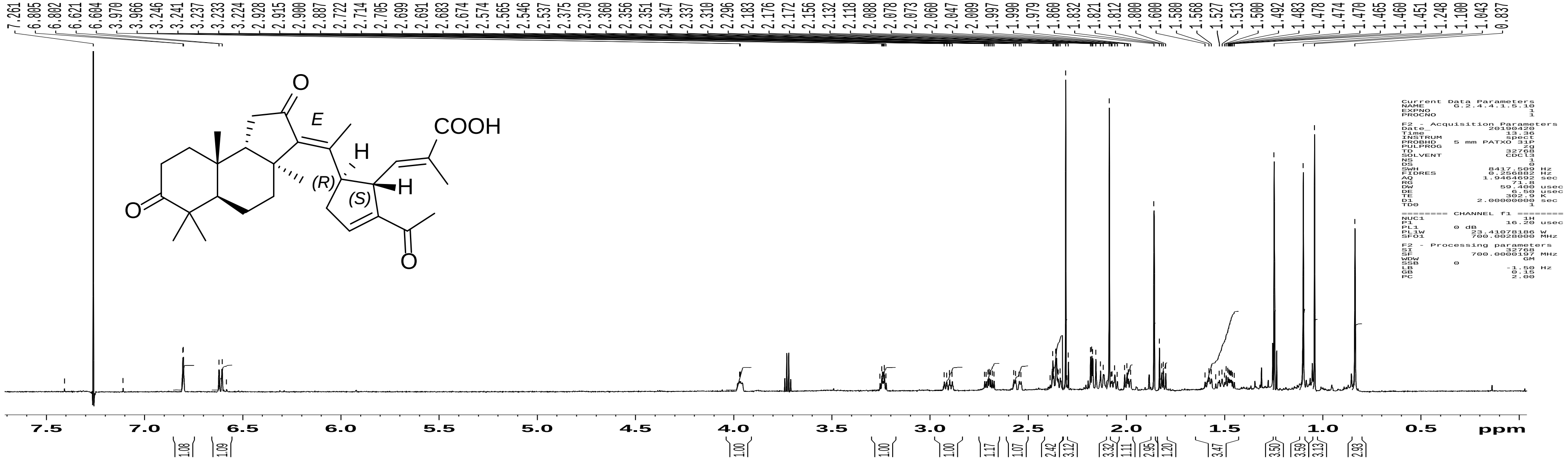

^{1}H NMR Spectrum of Globostelletin M from *Rhabdastrella globostellata*. (500 MHz, $CDCl_3$) [1]

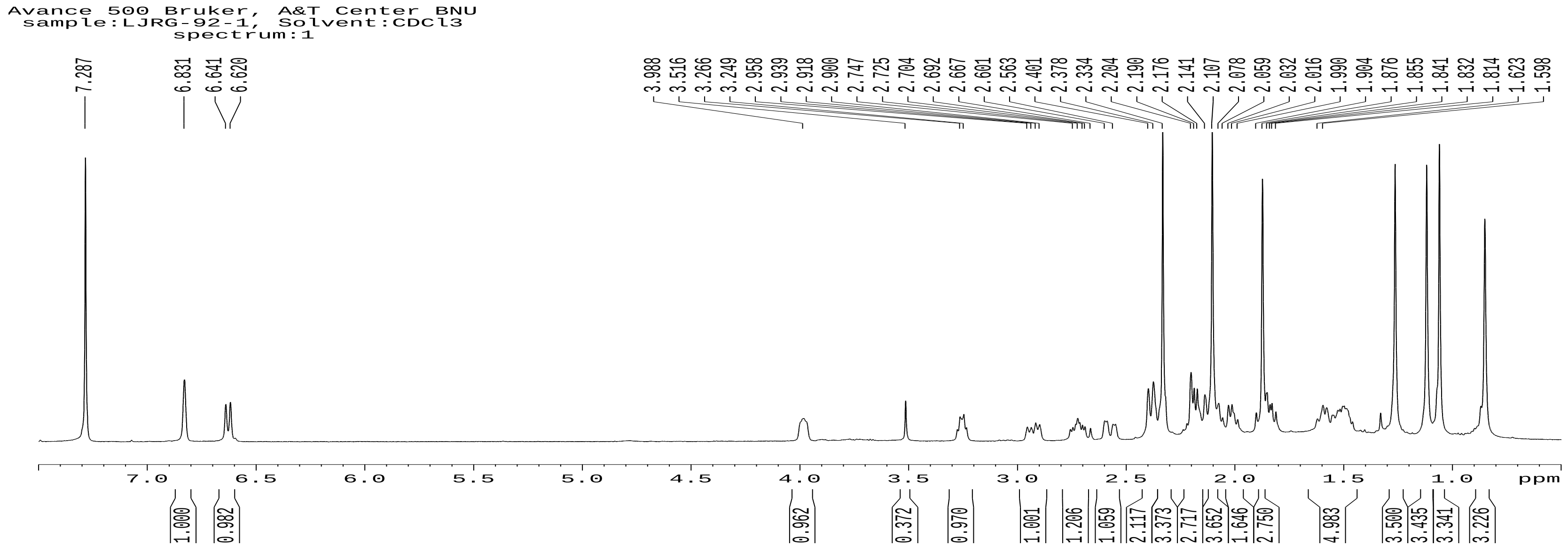

S60 ^{13}C NMR Spectrum of Globostelletin M from *Stelletta* sp. (176 MHz, $CDCl_3$)

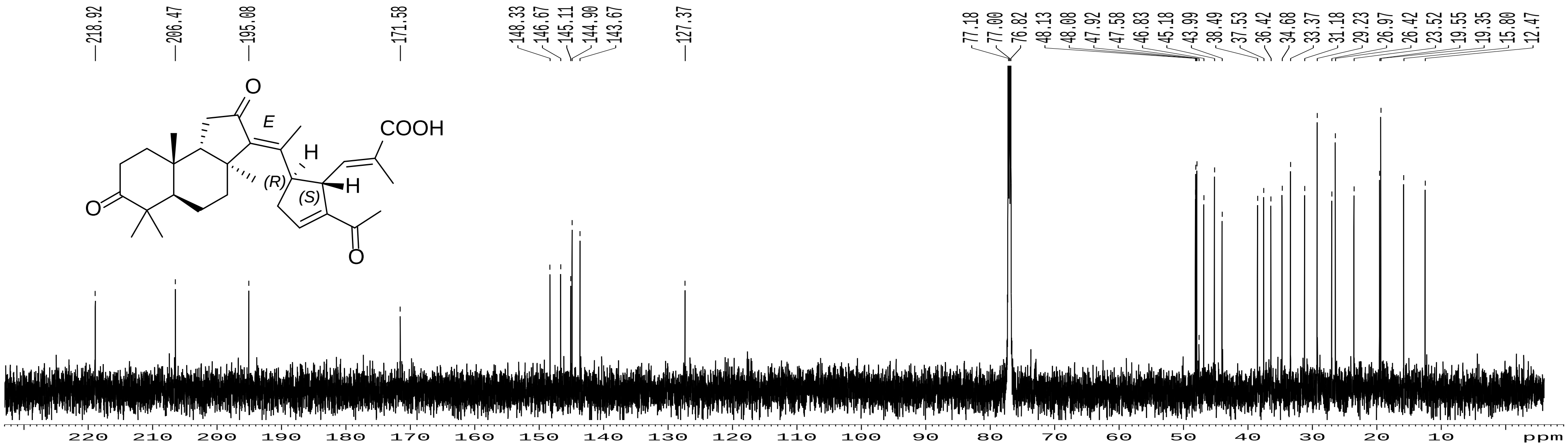

^{13}C NMR Spectrum of Globostelletin M from *Rhabdastrella globostellata* (125 MHz, $CDCl_3$) [1]

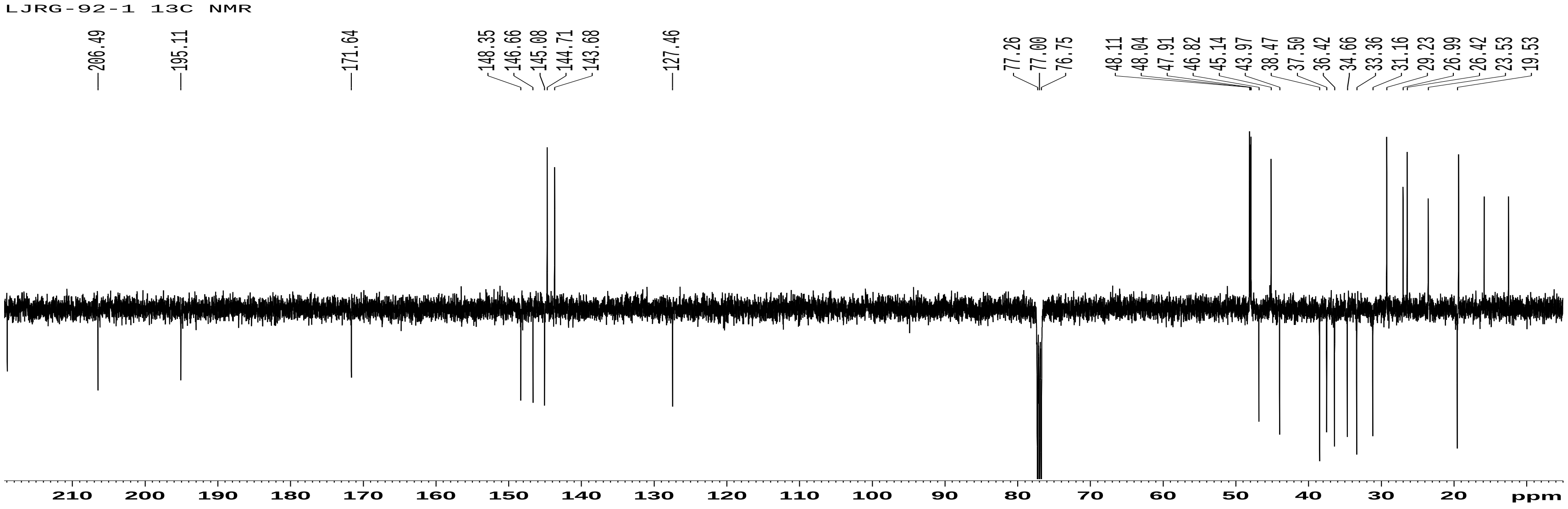

S61 ECD Spectrum of Globostelletin M from *Stelletta* sp. (EtOH)

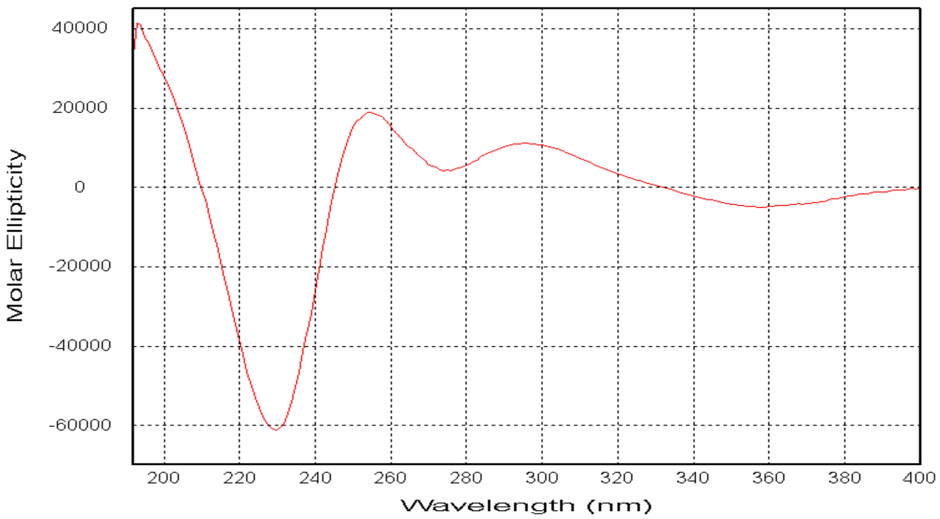

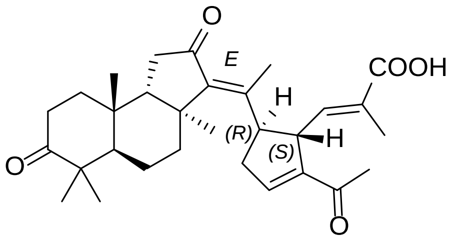

ECD Spectrum of Globostelletin M from *Rhabdastrella globostellata* [1]

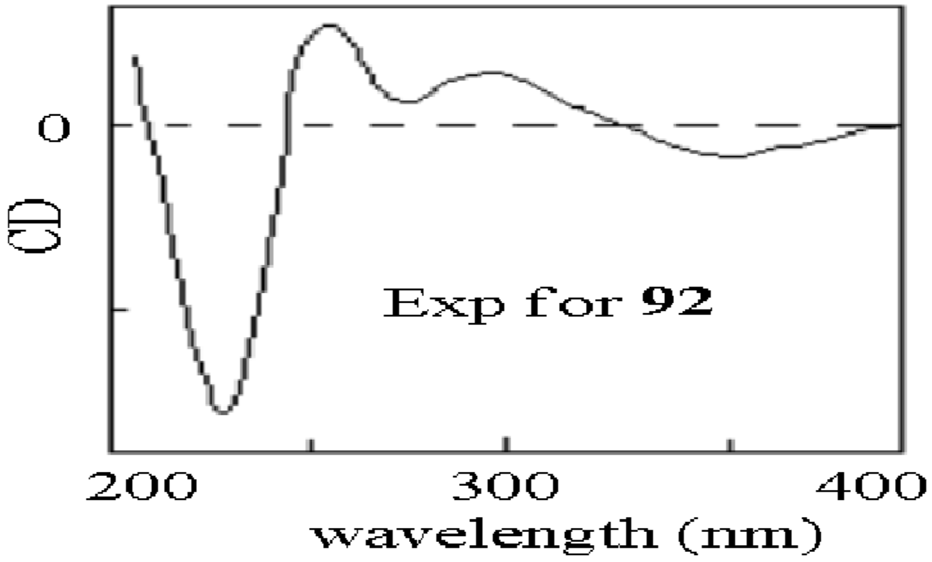

S62 HRESIMS and MS/MS Spectra (Negative Ion Mode) of Globostelletin N from *Stelletta* sp.

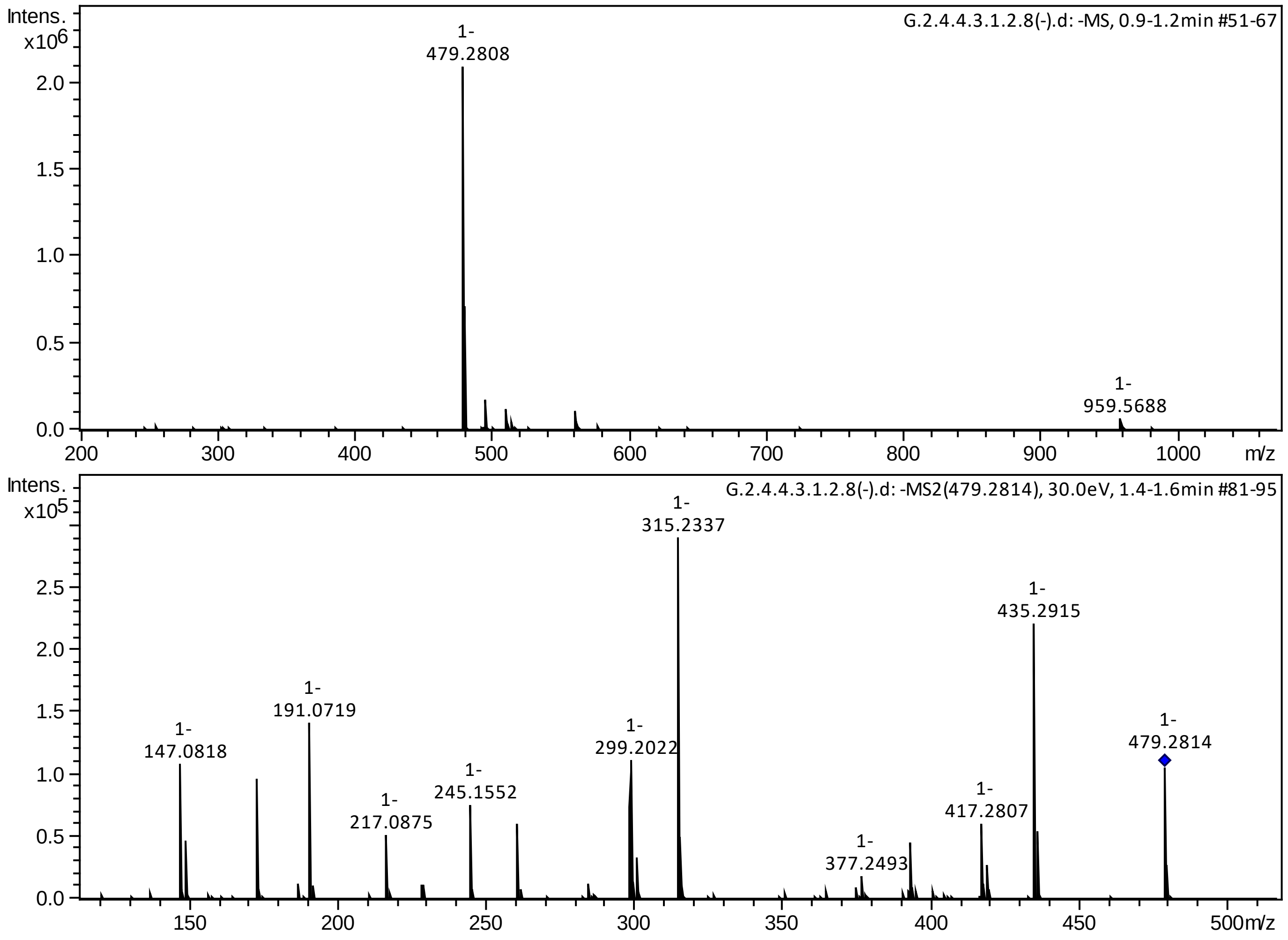

S63 ^{1}H NMR Spectrum of Globostelletin N from *Stelletta* sp. (700 MHz, $CDCl_3$)

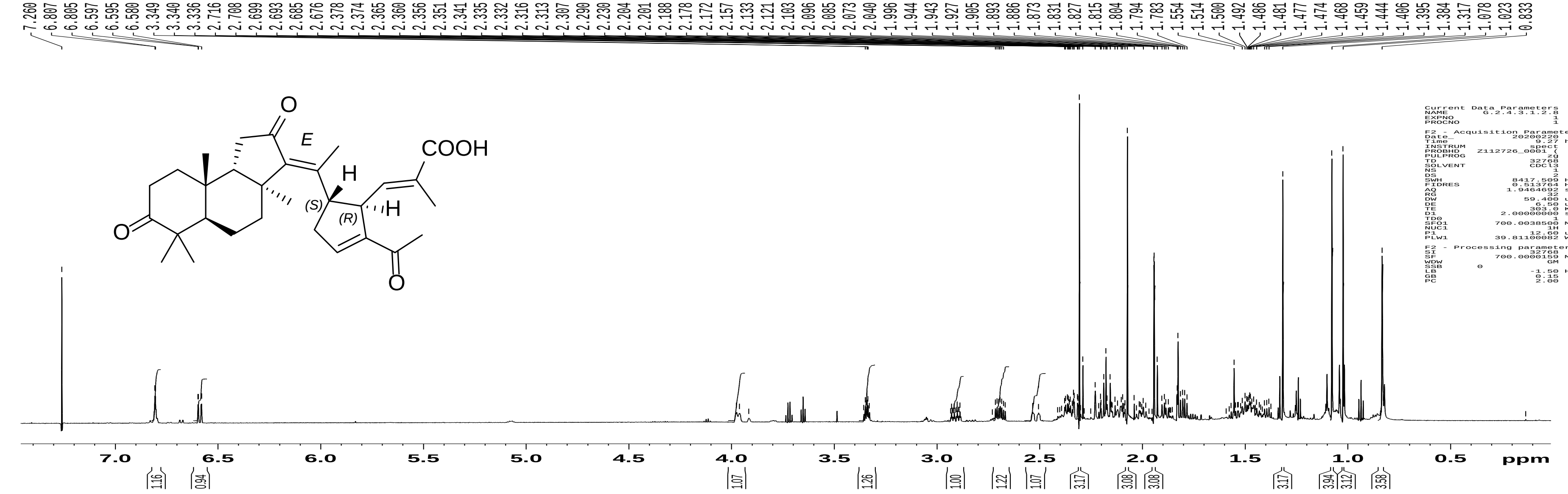

^{1}H NMR Spectrum of Globostelletin N from *Rhabdastrella globostellata*. (500 MHz, $CDCl_3$) [1]

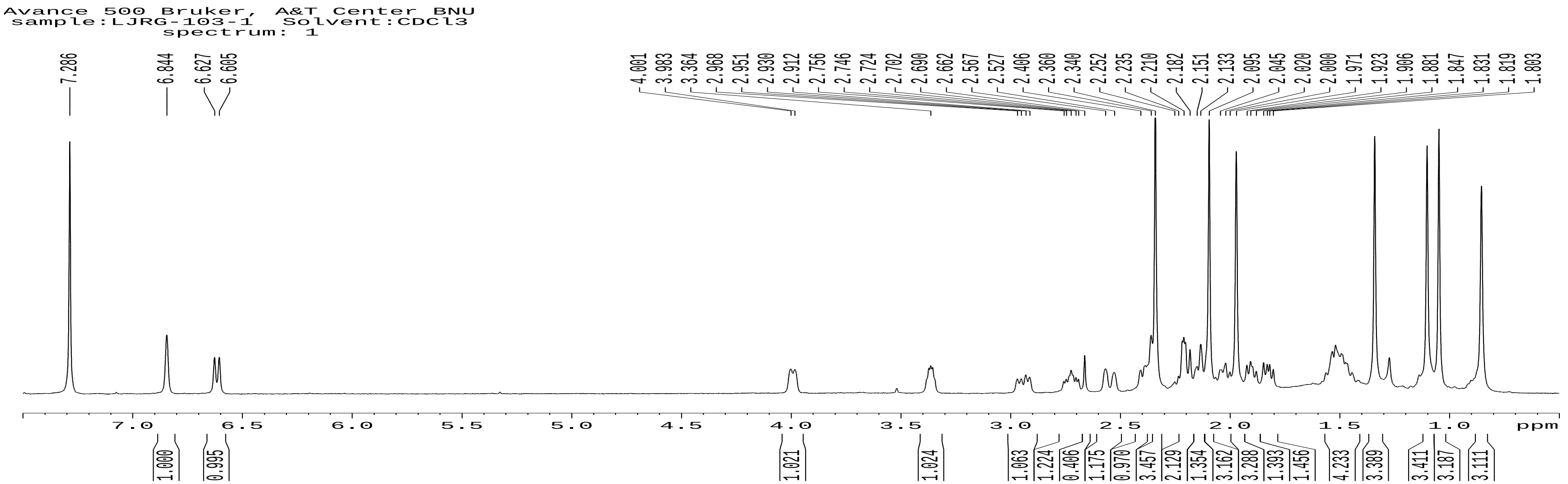

S64 ^{13}C NMR Spectrum of Globostelletin N from *Stelletta* sp. (176 MHz, $CDCl_3$)

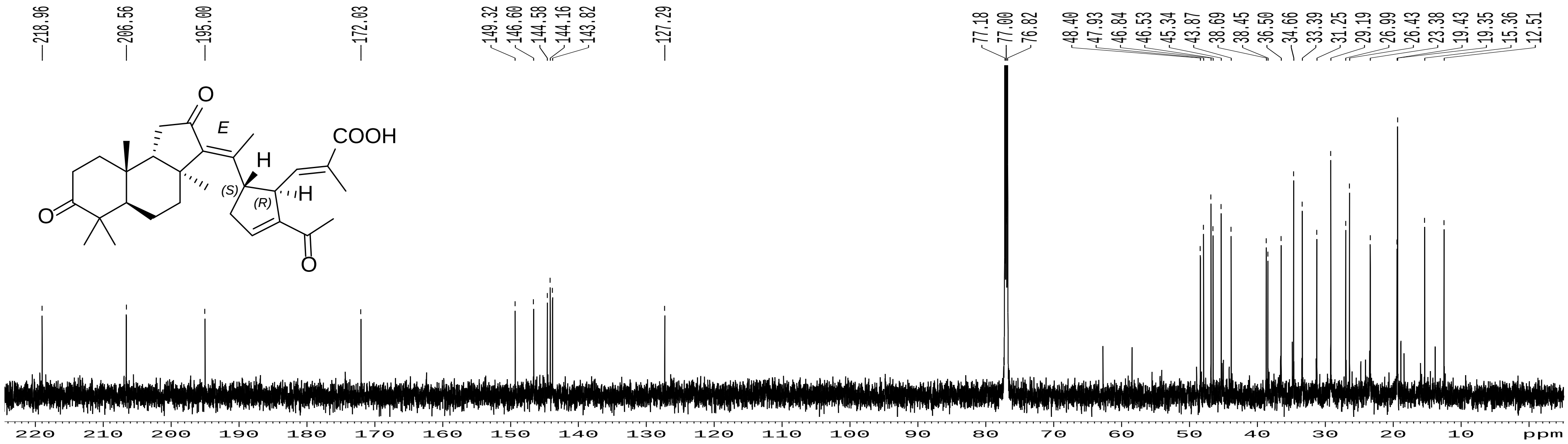

^{13}C NMR Spectrum of Globostelletin N from *Rhabdastrella globostellata* (125 MHz, $CDCl_3$) [1]

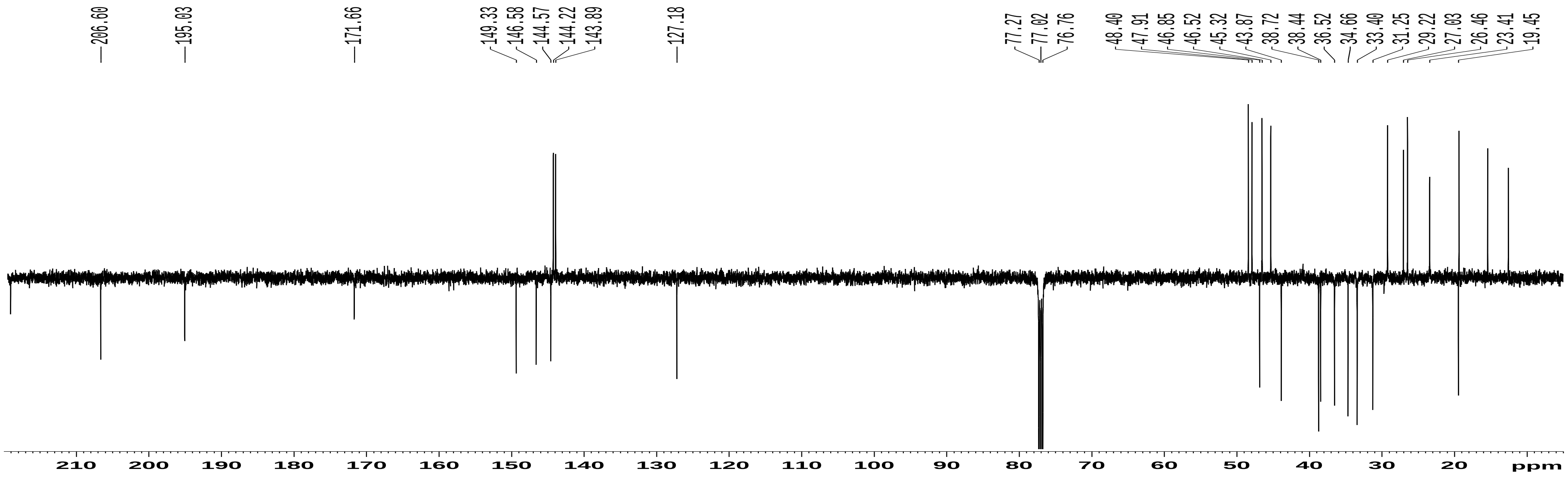

S65 ECD Spectrum of Globostelletin N from *Stelletta* sp. (EtOH)

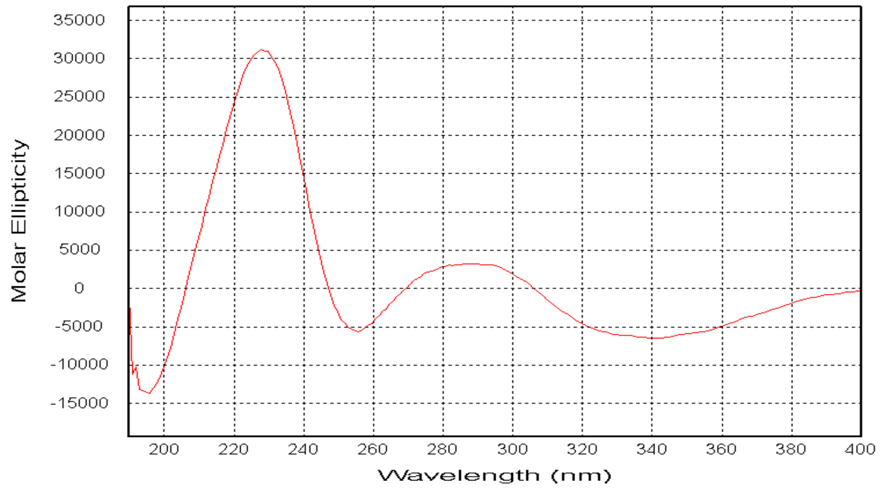

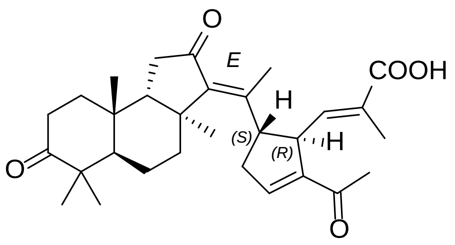

ECD Spectrum of Globostelletin N from *Rhabdastrella globostellata* [1]

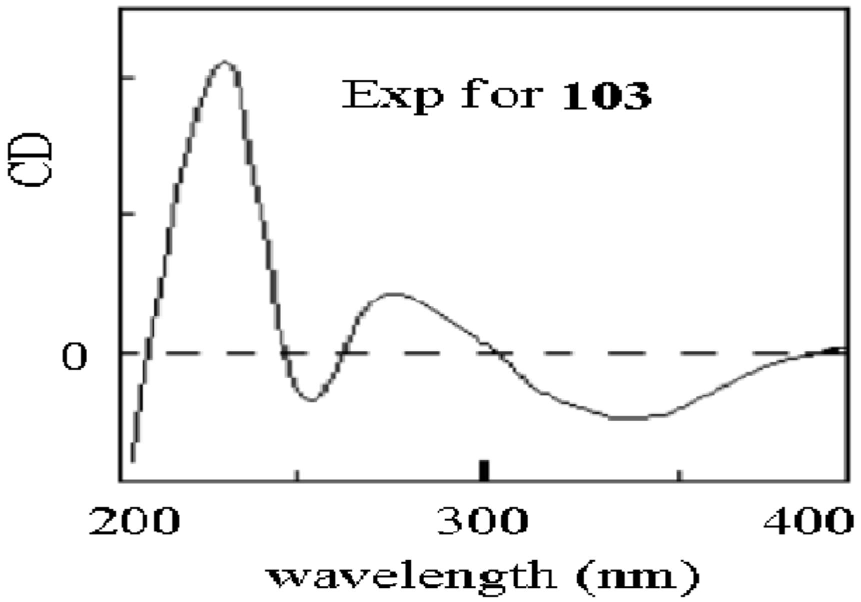

S66 Theoretical Modeling

The quantum-chemical calculations in C_2H_5OH solvent were done using density functional theory (DFT) with the nonlocal exchange-correlation functional B3LYP, the polarization continuum model (PCM) and split-valence basis sets 6-31G(d), implemented in the Gaussian 16 package of programs. The molecular cavity was modeled according to unified force field (*radii=UFF*). Chemical shifts (δ_H and δ_C) were calculated as: B3LYP/6-311G(d)_PCM//B3LYP/6-311G(d)_PCM in $CDCl_3$. Theoretical $\delta_{C,calc}$ values, calculated for all carbon atoms of compound **5** and **6**, are slightly overestimated – the linear regression analysis gave the next relations: $\delta_{C,calc}$ = 5.980 + 1.016·$\delta_{C,exp}$, ppm (**5**) and $\delta_{C,calc}$ = 4.020 + 1.006·$\delta_{C,exp}$, ppm (**6**), correspondingly.

Calculation of ECD spectra for chosen stereoisomer were done according general scheme. First, the conformational analysis was done and the statistical weights (g_{im}) of different conformations were obtained according to equation:

$$g_{im} = e^{-\Delta E_{im}/RT} \Big/ \sum_i e^{-\Delta E_{im}/RT} \qquad (1),$$

where the summation was done over all possible conformations of stereoisomer under study; the subscript "m" denotes conformation, for which *E* is minimal. Conformations, for which electronic energies are in the range $0 \le \Delta E_{im} \le 3$ kcal · mol^{-1}, were chosen for further calculation of ECD spectra. The excitation energies and the rotatory strengths were calculated using time-dependent density functional theory (TDDFT). Each individual transition from electronic ground state to the *i*-th calculated excited electronic state ($1 \le i \le 35$) was simulated as a Gauss-type function. For all compounds the same value ζ = 0.24 eV for the bandwidths at 1/*e* peak heights was used. The UV shift $\Delta\lambda$ = -10 nm was used. The total theoretical ECD spectrum was obtained after statistical averaging over all selected conformations:

$$\Delta\varepsilon_{calc}(\lambda) = \sum_i g_i \cdot \Delta\varepsilon_{i,calc}(\lambda) \qquad (2),$$

where *i* – denotes different conformations of stereoisomer under study.

The scaled theoretical and experimental ECD spectra were obtained according to equation:

$$\Delta\varepsilon_{scaled}(\lambda) = \Delta\varepsilon(\lambda) \Big/ \left|\Delta\varepsilon(\lambda_{peak})\right| \qquad (3),$$

where the denominator $|\Delta\varepsilon(\lambda_{peak})|$ is a modulo of the peak value for the chosen characteristic band in corresponding ECD spectrum.

S67 Optimized geometries and statistical weights of main and minor conformations of Stellettin Q (**1**)

Main conformations

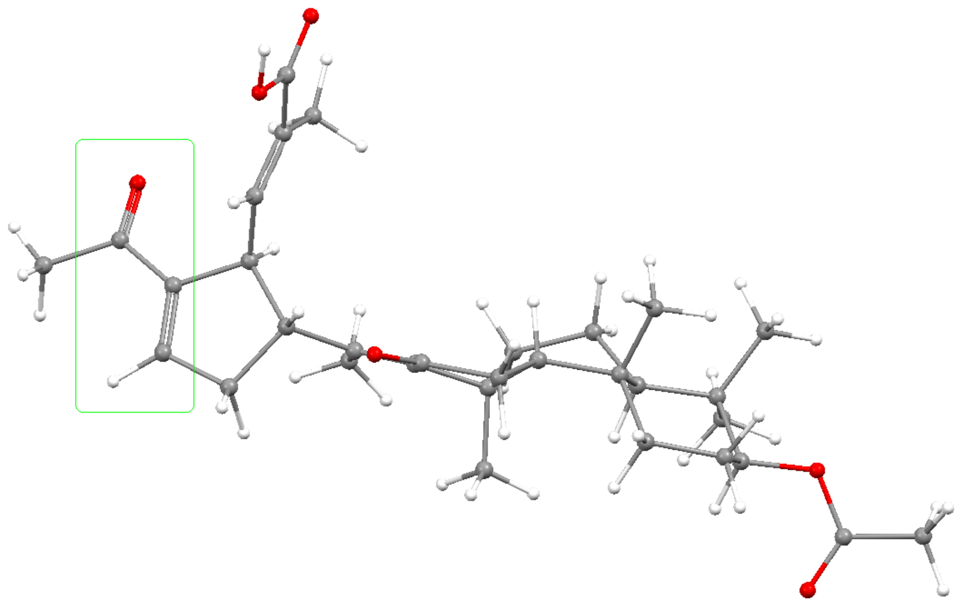

[94%]

Minor conformations

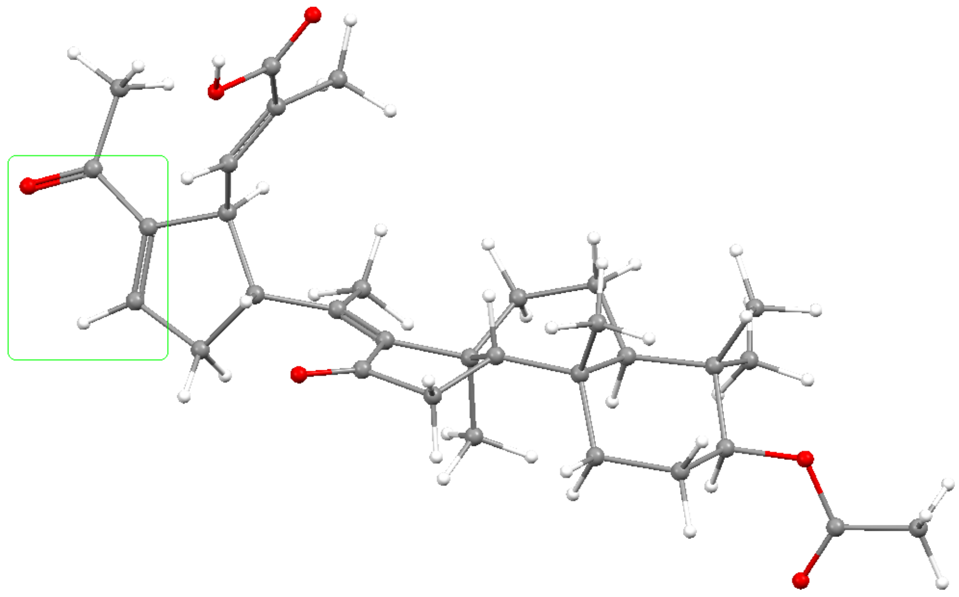

[< 6%]

S68 Optimized geometries and statistical weights of main and minor conformations of Stellettin R (**2**)

Main conformations

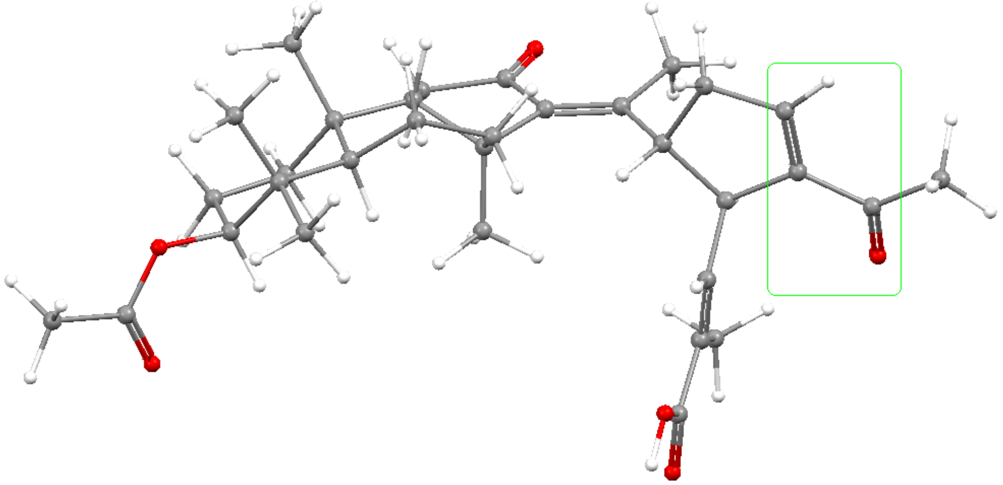

[93%]

Minor conformations

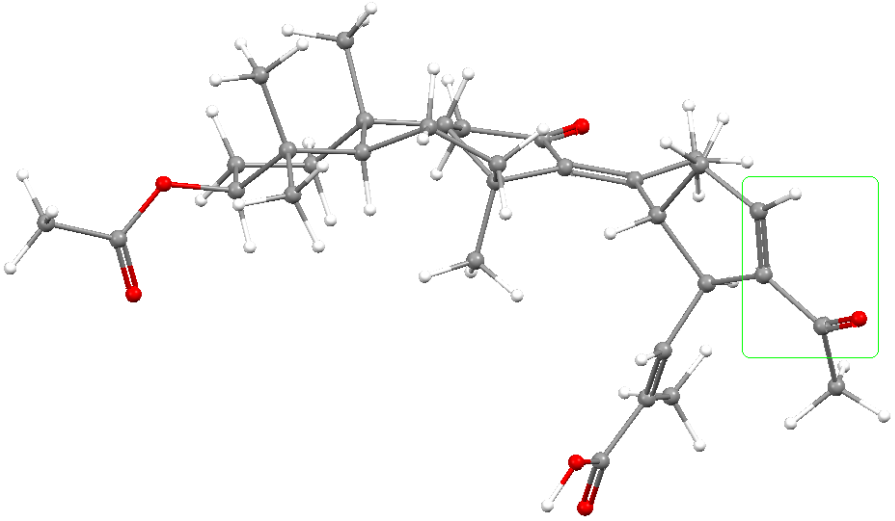

[7%]

S69 The computational ECD results for Globostelletin K

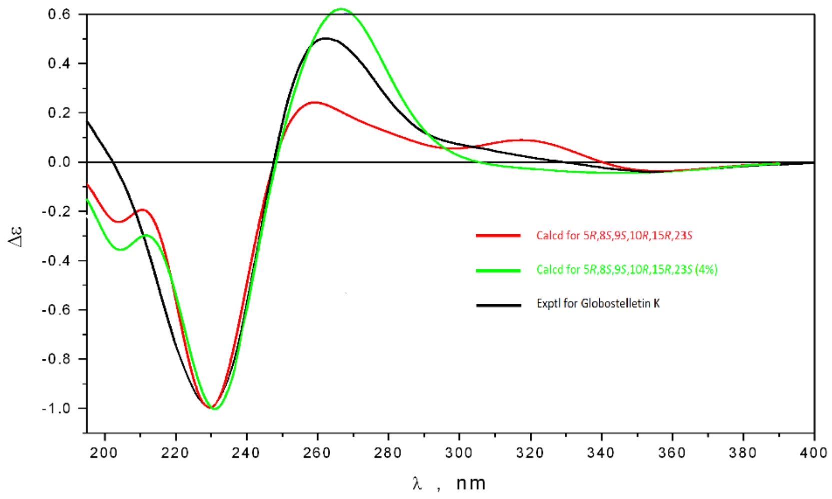

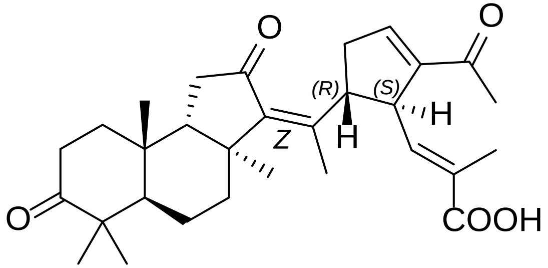

Optimized geometries and statistical weights of main and minor conformations of Globostelletin K.

Main conformations

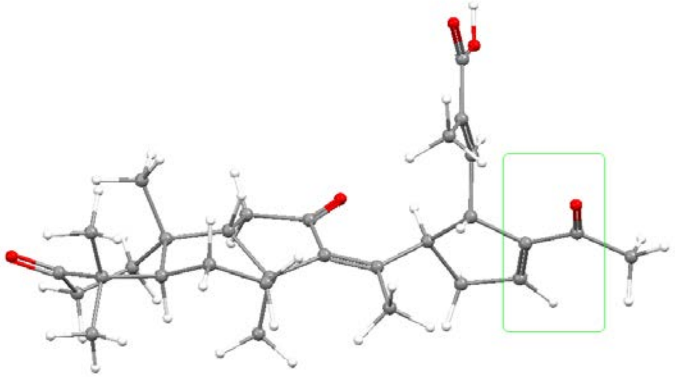

[96%]

Minor conformations

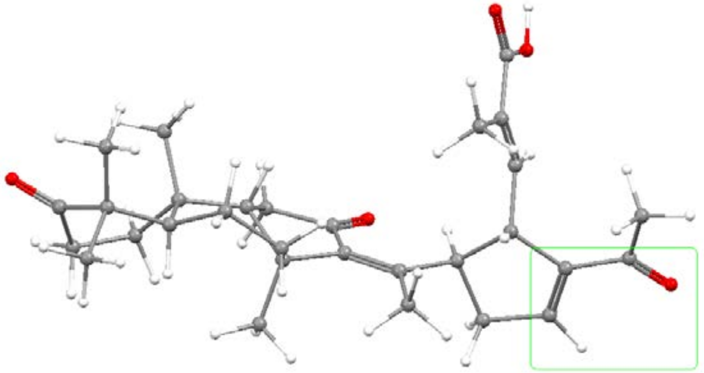

[4%]

S70 The computational ECD results for Globostelletin M

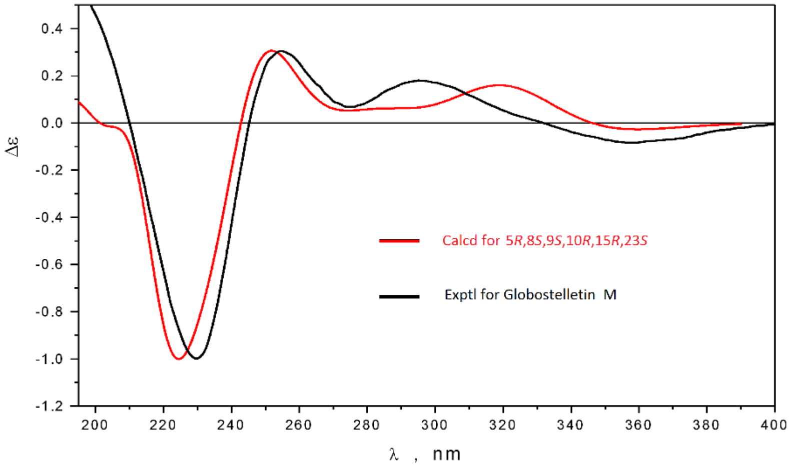

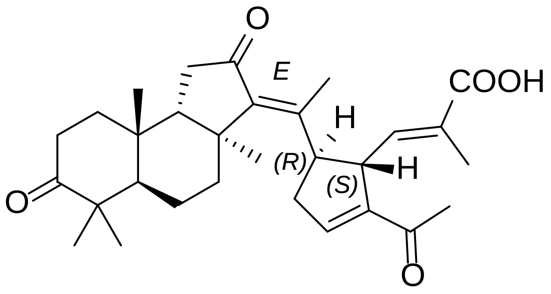

Optimized geometries and statistical weights of main and minor conformations of Globostelletin M.

Main conformations

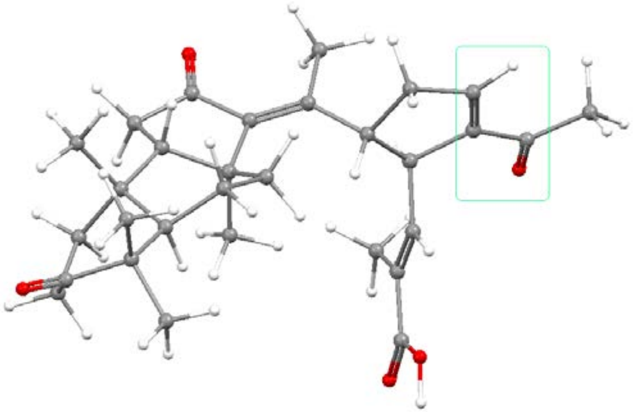

[94%]

Minor conformations

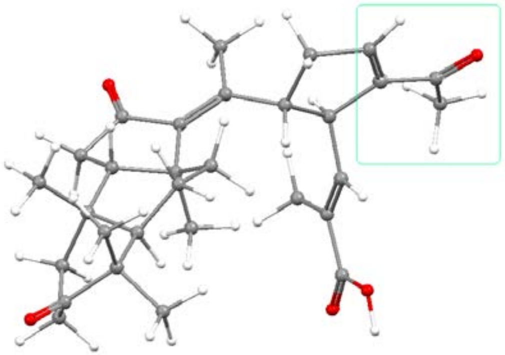

[6%]

S71 The computational ECD results for Globostelletin N

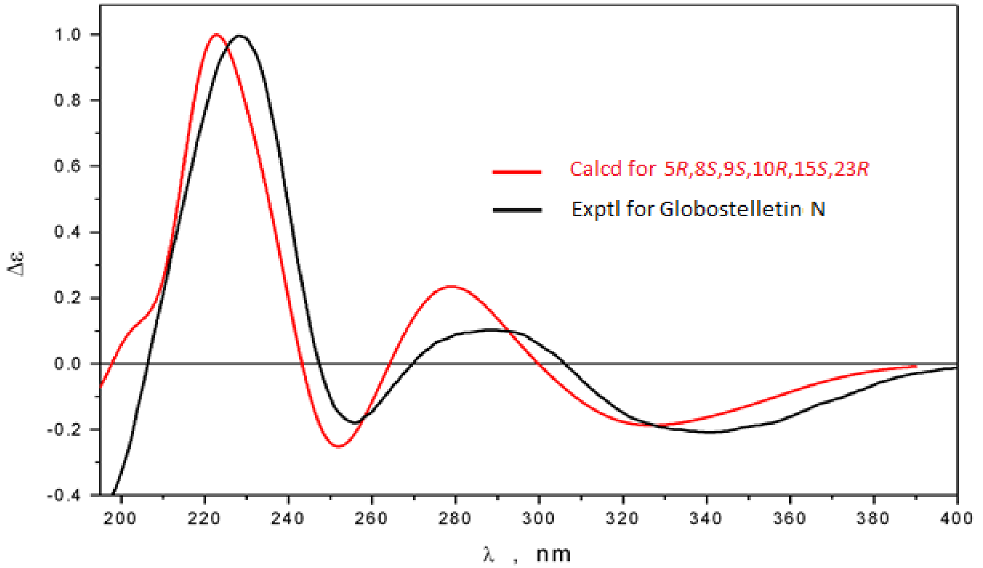

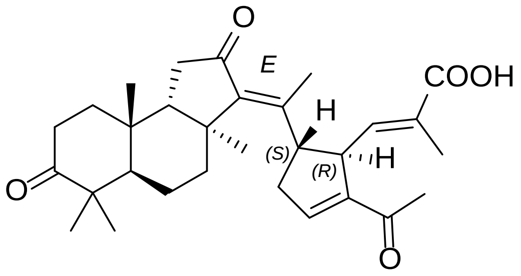

Optimized geometries and statistical weights of main and minor conformations of Globostelletin N.

Main conformations

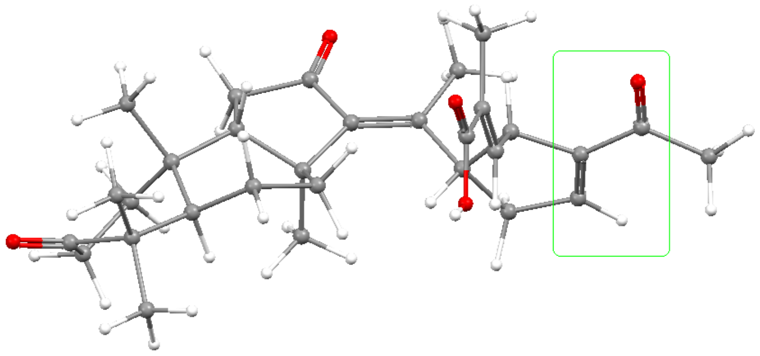

[98%]

Minor conformations

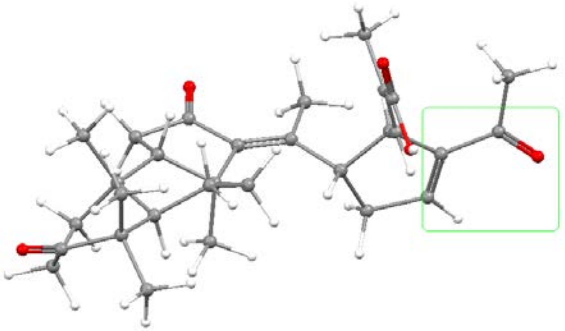

[2%]

S72 The computational ECD results calculated for all possible 15,23-stereoisomers of studied Globostelletins K, M, N, and new Stellettins Q (**1**) and R (**2**)

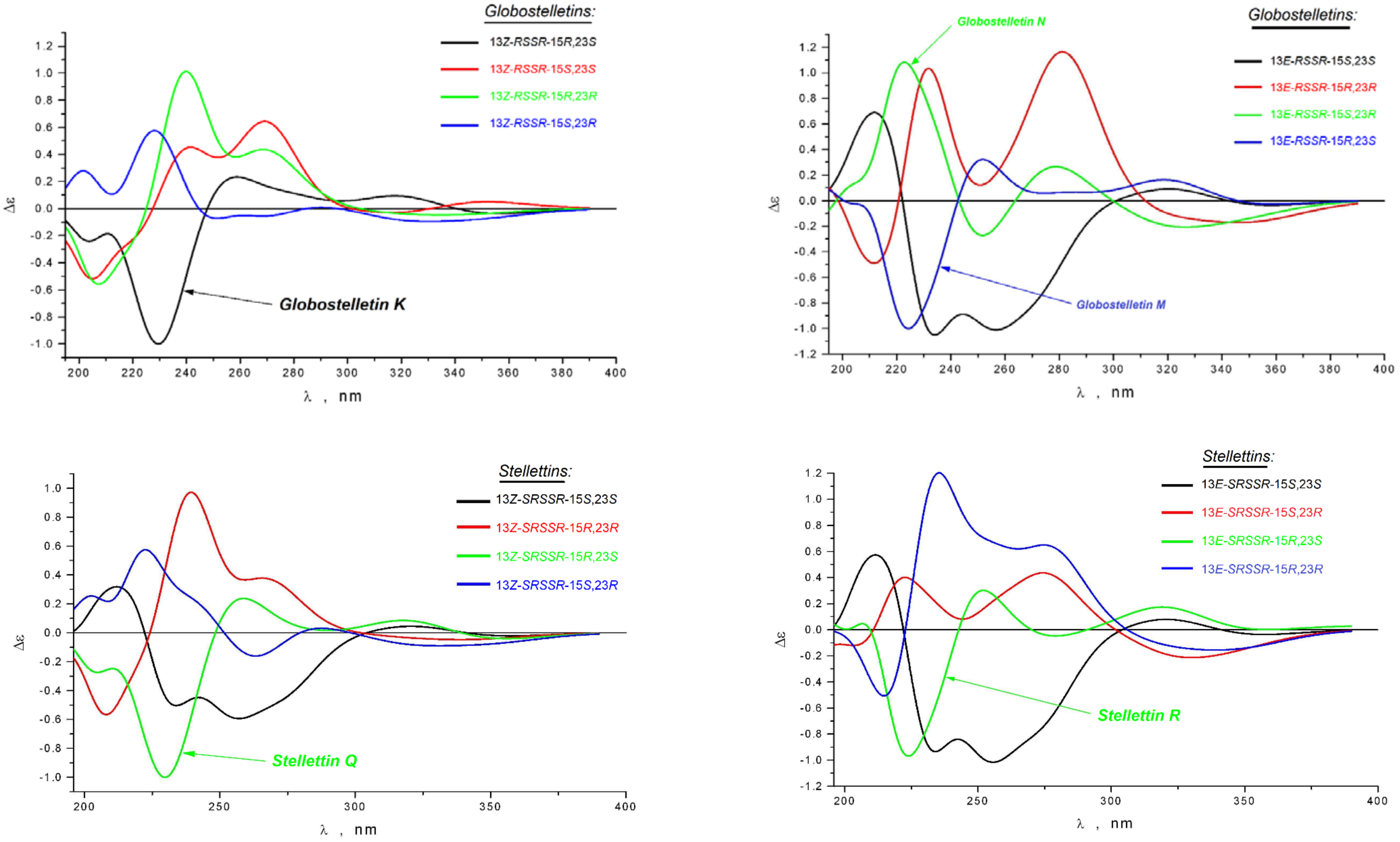

S73 The Isolation Scheme

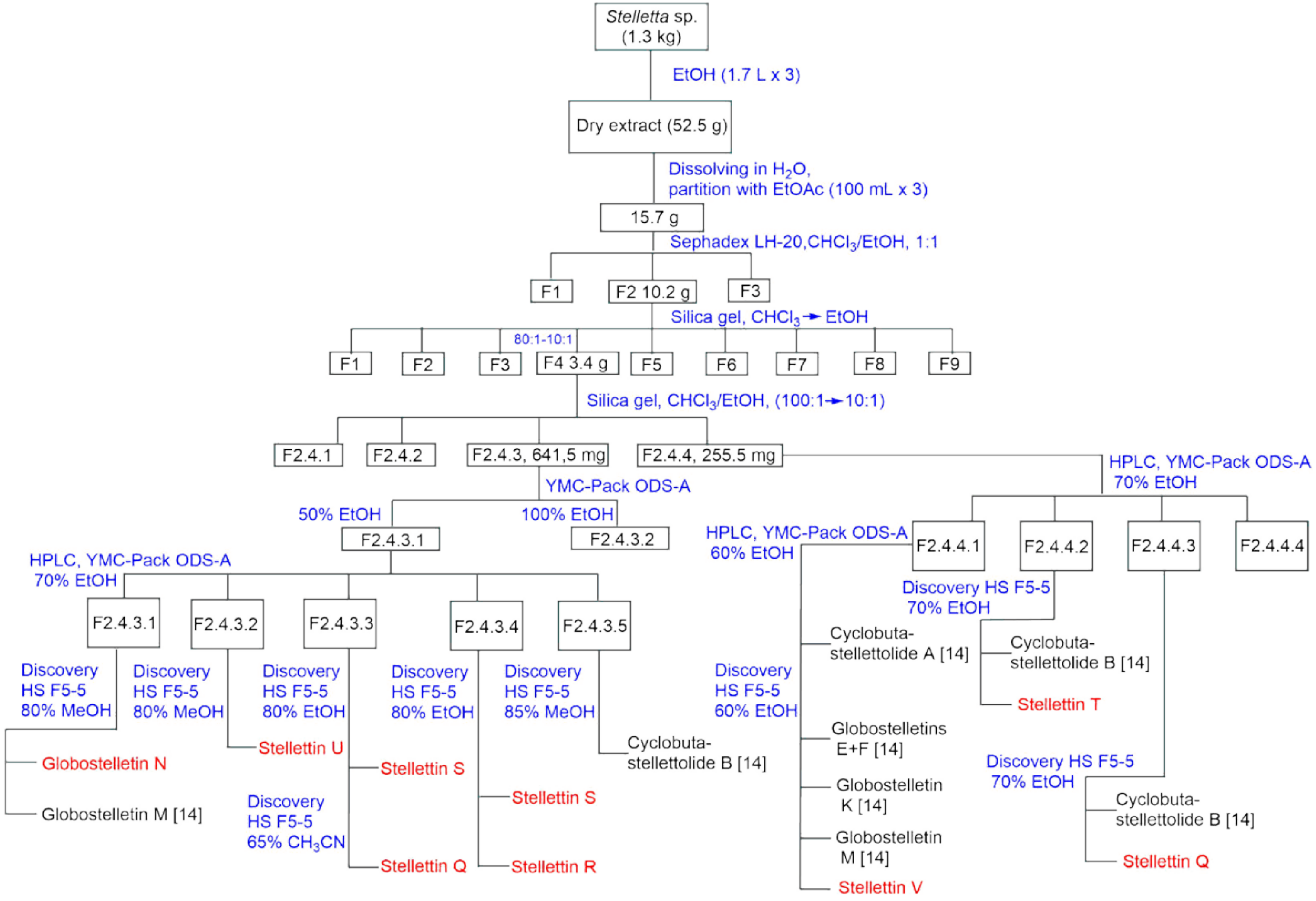

Two Cyclobutastellettolides A and B together with known Globostelletins M, K, E, F were reported in our previous paper [14].

[14] Kolesnikova, S.A.; Lyakhova, E.G.; Kalinovsky, A.I.; Berdyshev, D.V.; Pislyagin, E.A.; Popov, R.S.; Grebnev, B.B.; Makarieva, T.N.; Minh, C.V.; Stonik, V.A. Cyclobutastellettolides A and B, C_{19} norterpenoids from a *Stelletta* sp. marine sponge. *J. Nat. Prod*. **2019**, 82, 3196–3200.
